# Supplementary material for: Development of 18F-Labeled Bispyridyl Tetrazines for In Vivo Pretargeted PET Imaging
Source: Pharmaceuticals (Basel). 2022 Feb 18;15(2):245. doi: 10.3390/ph15020245 (PMC8879724; doi:10.3390/ph15020245)

Supporting Information

Development of ^{18}F-Labeled Bispyridyl Tetrazines for In Vivo Pretargeted PET Imaging

Rocío García-Vázquez [1,2,†], Jesper Tranekjær Jørgensen [3,†], Klas Erik Bratteby [1,2,4], Vladimir Shalgunov [1,2], Lars Hvass [3], Matthias M. Herth[1,2,*], Andreas Kjær [3,*] and Umberto Maria Battisti [1,*]

1 Department of Drug Design and Pharmacology, Faculty of Health and Medical Sciences, University of Copenhagen, Universitetsparken 2, 2100 Copenhagen, Denmark; rociogv@sund.ku.dk (R.G.-V.); klas.bratteby@sund.ku.dk (K.B.); vladimir.shalgunov@sund.ku.dk (V.S)

2 Department of Clinical Physiology, Nuclear Medicine & PET, Rigshospitalet, Blegdamsvej 9, 2100 Copenhagen, Denmark

3 Cluster for Molecular Imaging, Department of Biomedical Sciences, University of Copenhagen, Blegdamsvej 3, 2100 Copenhagen, Denmark; jespertj@sund.ku.dk (J.T.J.); lars.hvass@sund.ku.dk (L.H.)

4 Department of Radiation Physics, Skåne University Hospital, Barngatan 3, 22242 Lund, Sweden

* Correspondence: matthias.herth@sund.ku.dk (M.M.H.); akjaer@sund.ku.dk (A.K.); umberto.battisti@sund.ku.dk (U.M.B.)

† These authors contributed equally to the work.

*2-Fluoroethyl 4-(1,2,4,5-tetrazin-3-yl)benzoate (**3**)*

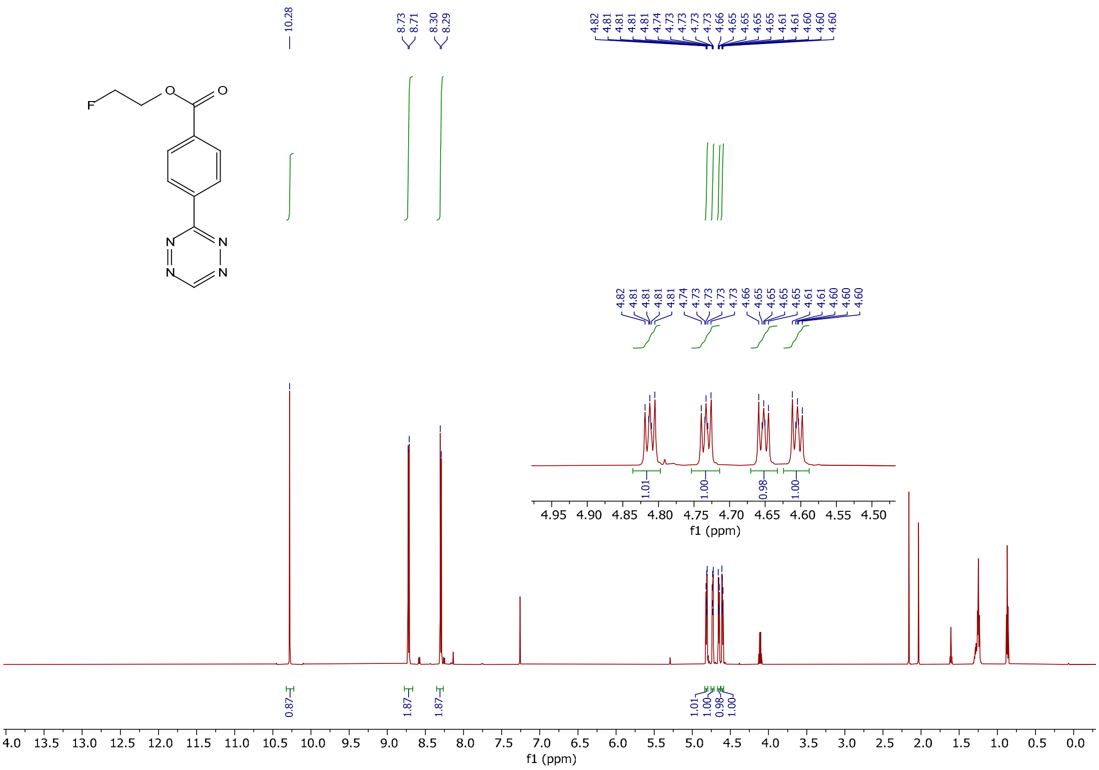

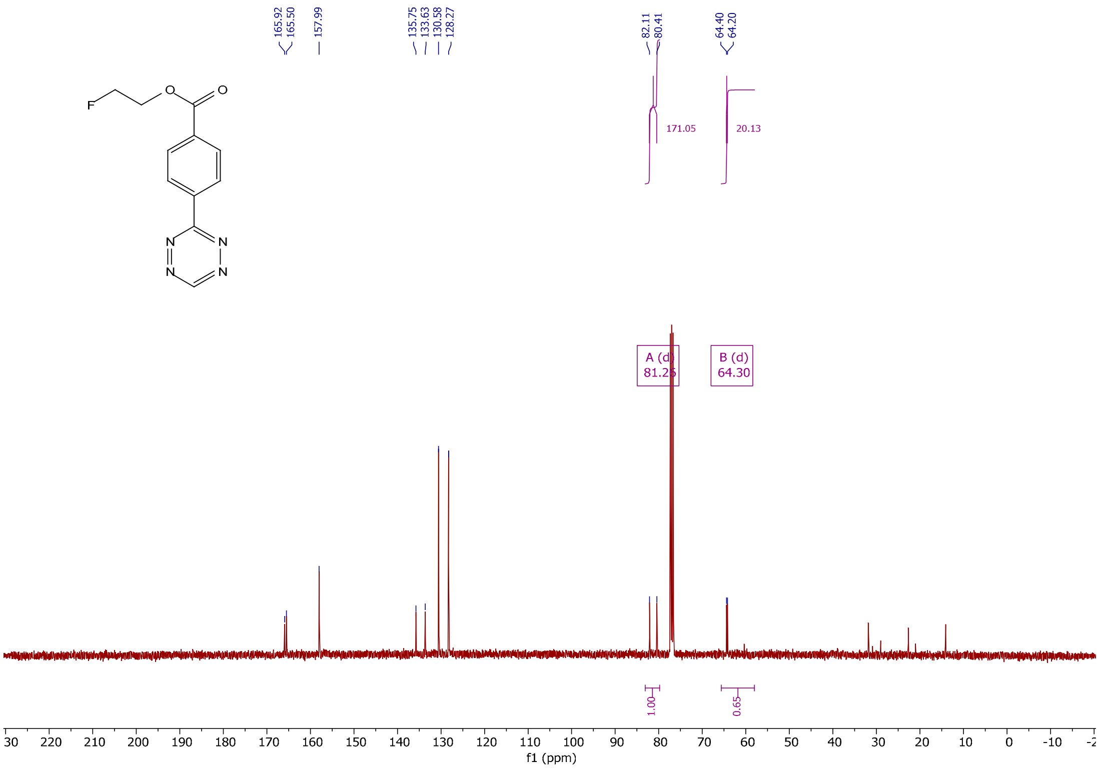

*2-(((4-Nitrophenyl)sulfonyl)oxy)ethyl 4-(1,2,4,5-tetrazin-3-yl)benzoate (**3a**)*

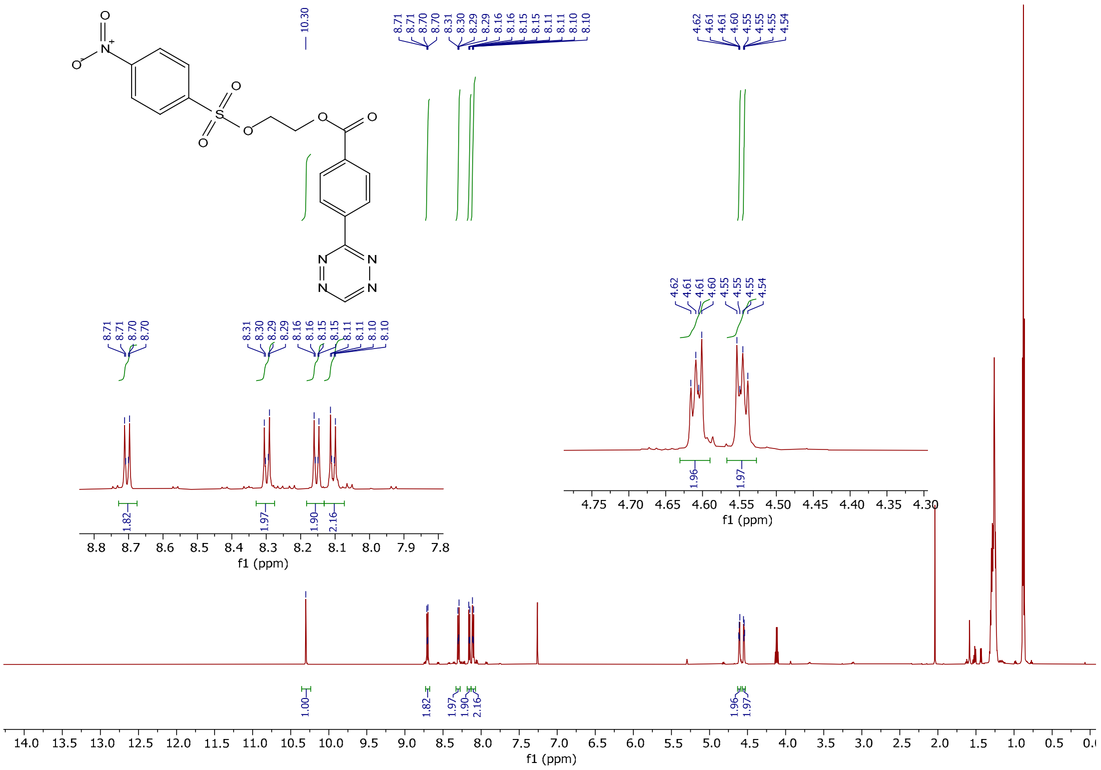

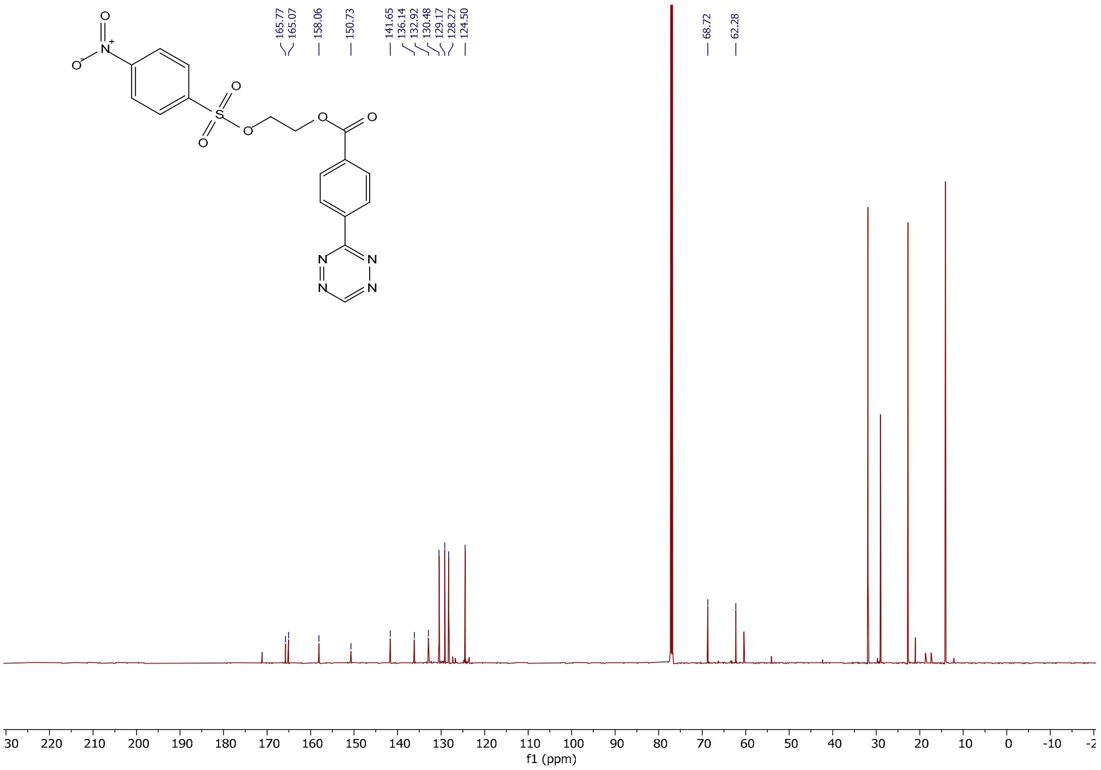

*N-(2-fluoroethyl)-4-(1,2,4,5-tetrazin-3-yl)benzamide (**4**)*

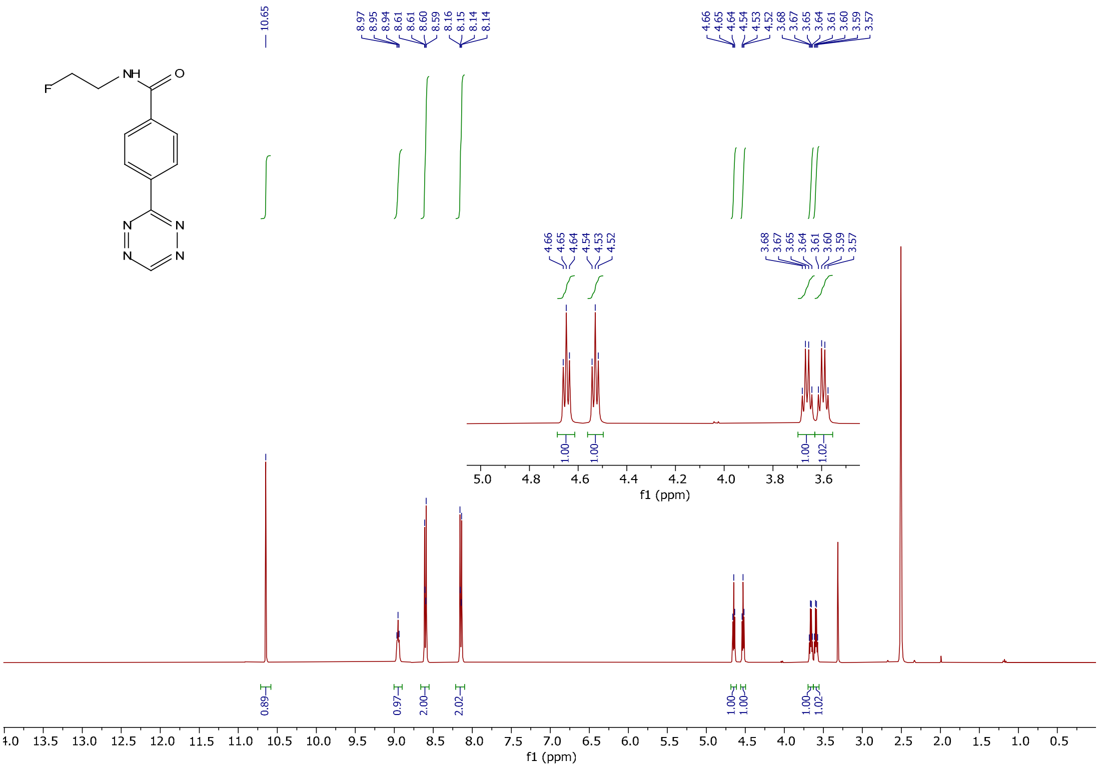

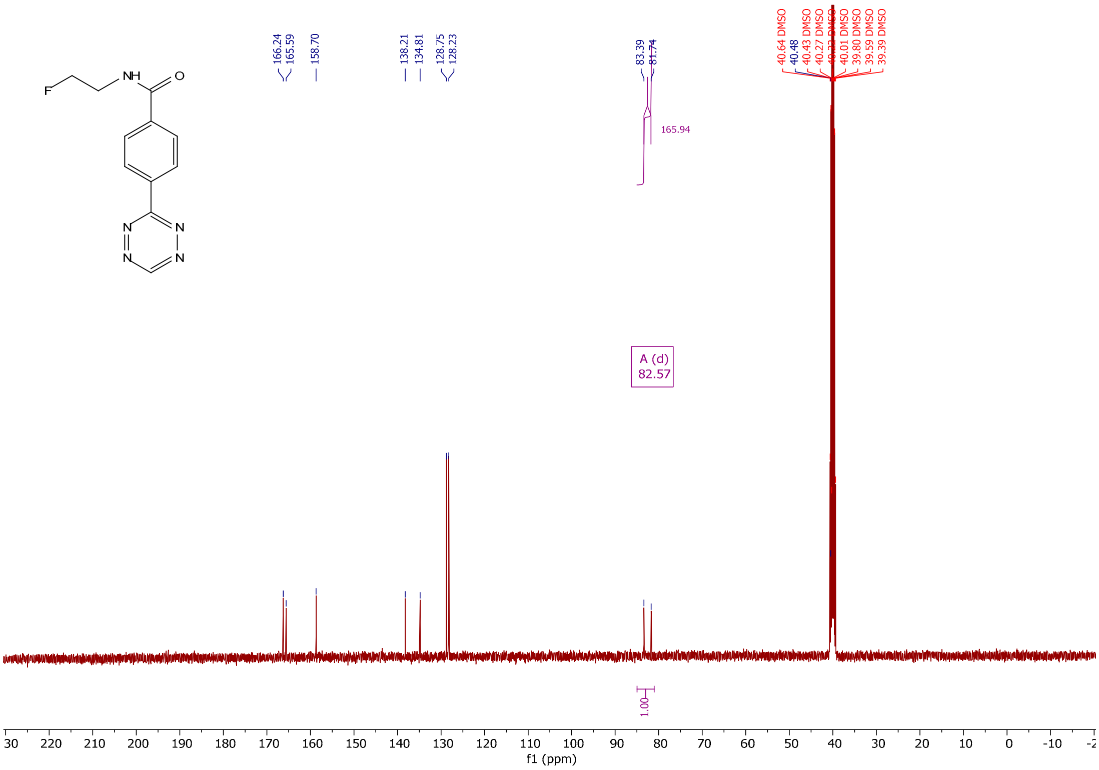

*2-Fluoroethyl 2-(4-(1,2,4,5-tetrazin-3-yl)phenyl)acetate (**5**)*

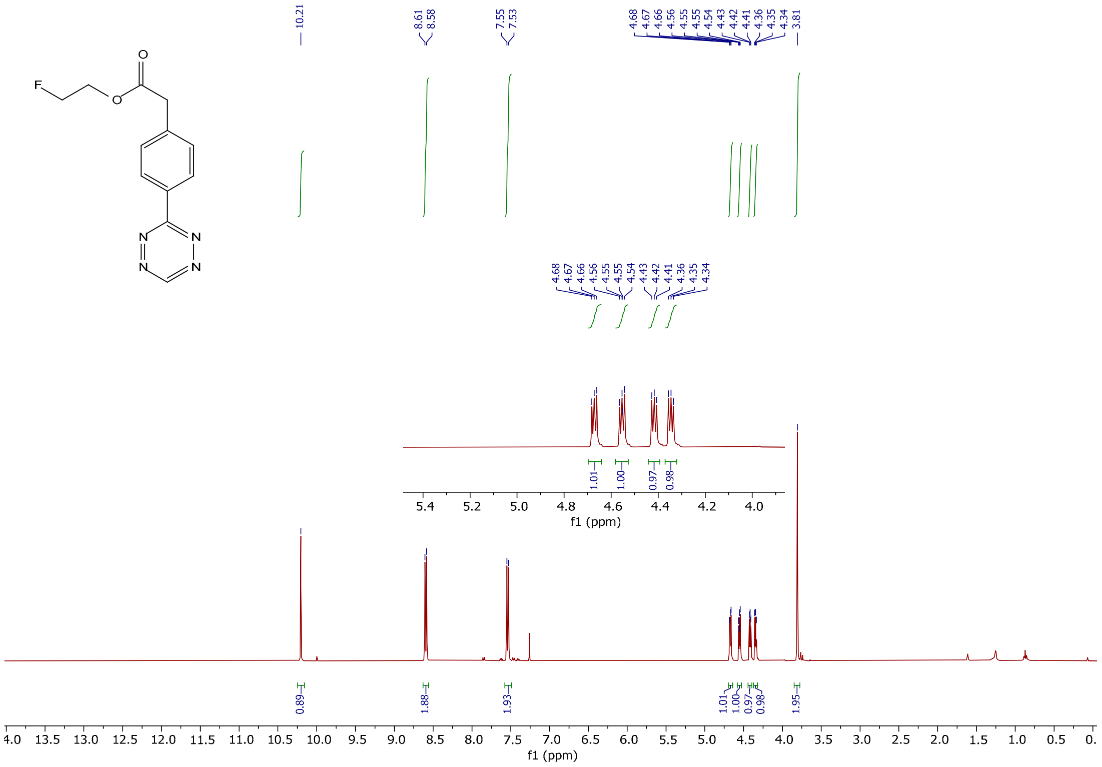

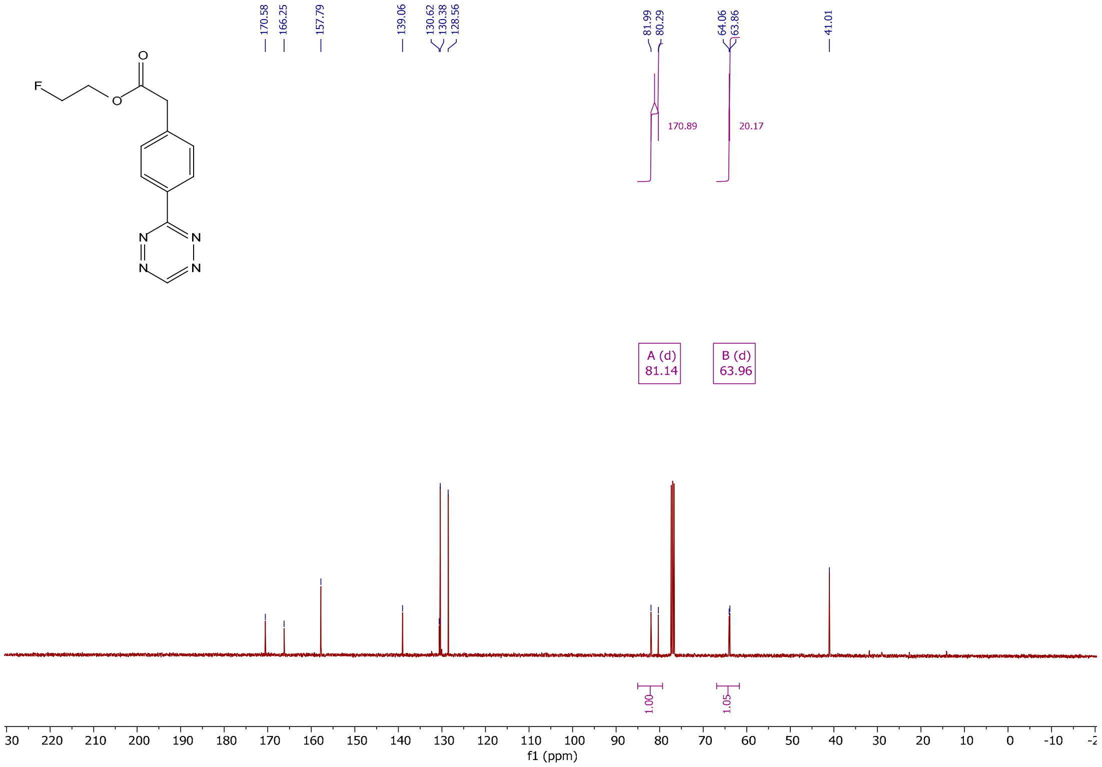

*2-(((4-Nitrophenyl)sulfonyl)oxy)ethyl 2-(4-(1,2,4,5-tetrazin-3-yl)phenyl)acetate (**5a**)*

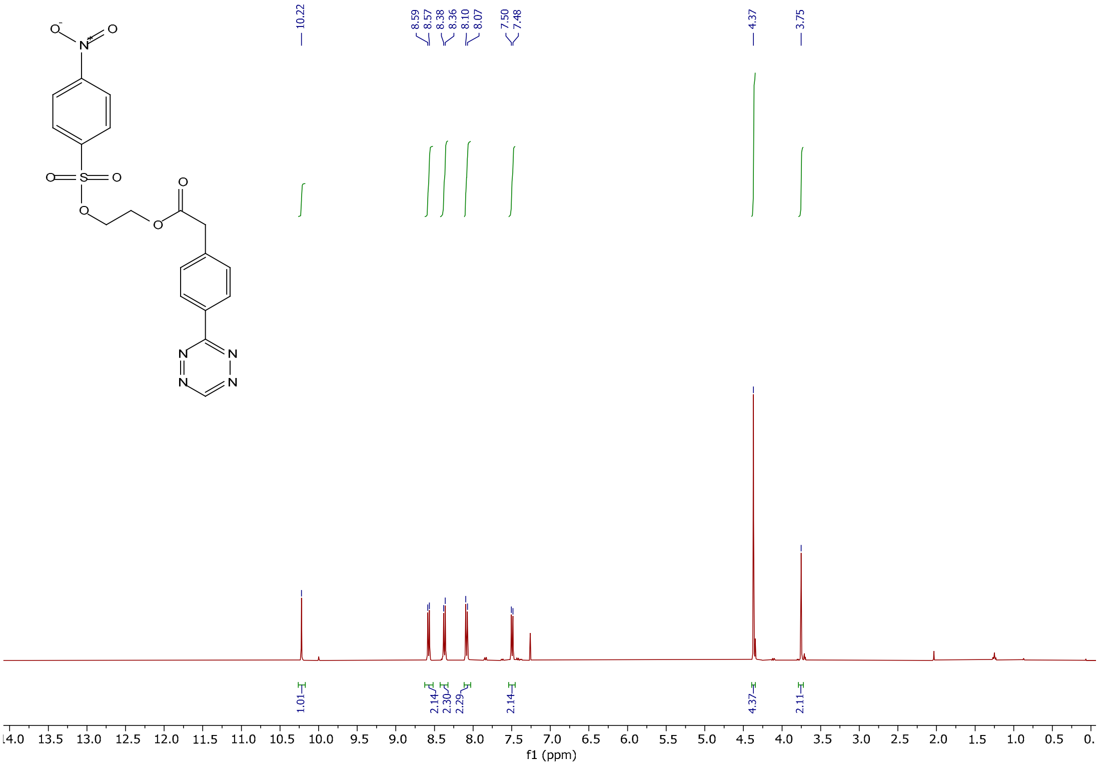

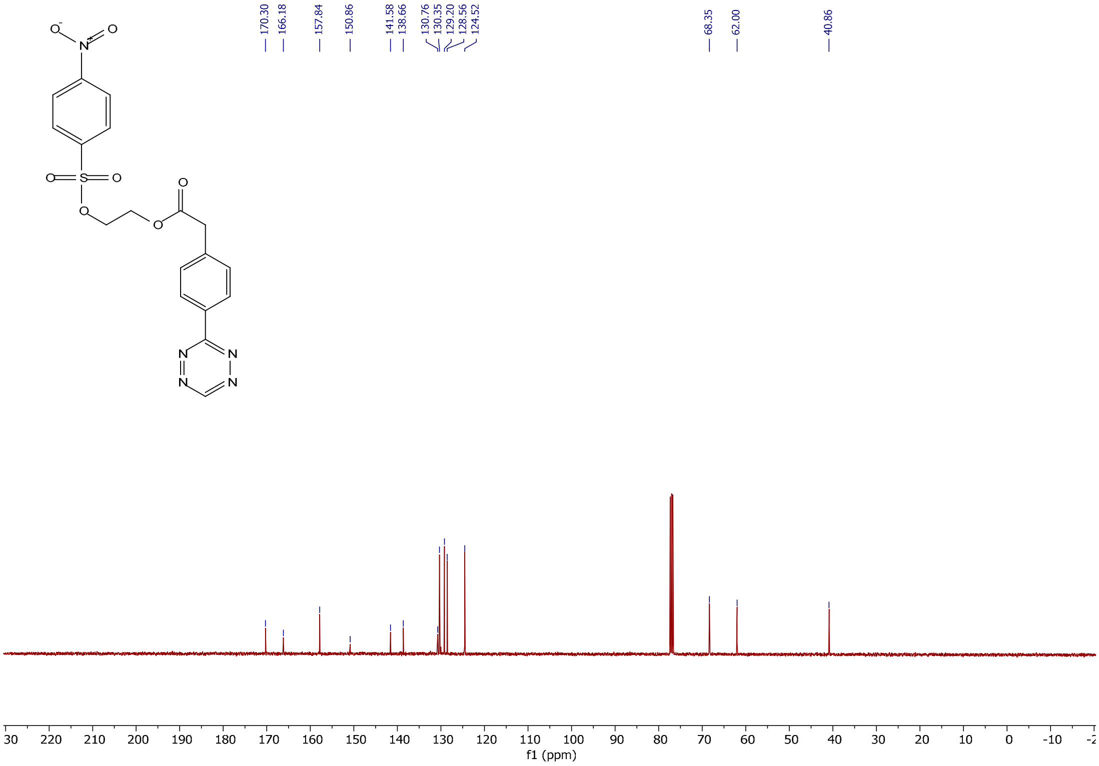

*2-(4-(1,2,4,5-Tetrazin-3-yl)phenyl)-N-(2-fluoroethyl)acetamide (**6**)*

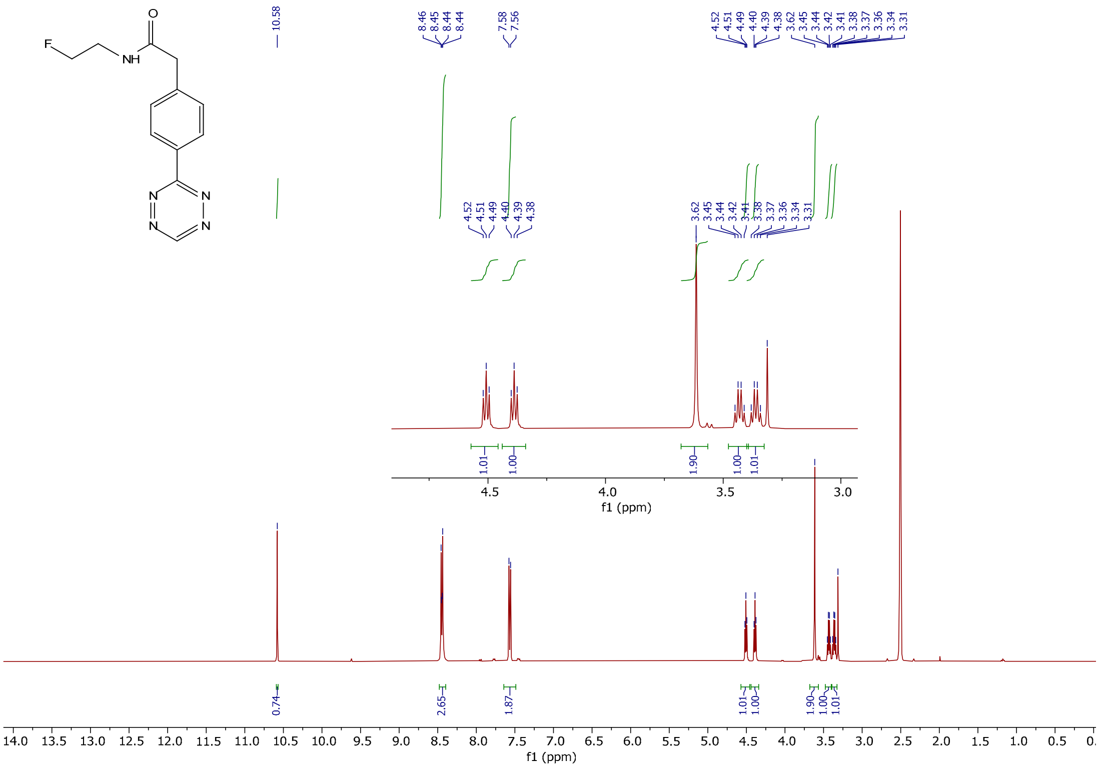

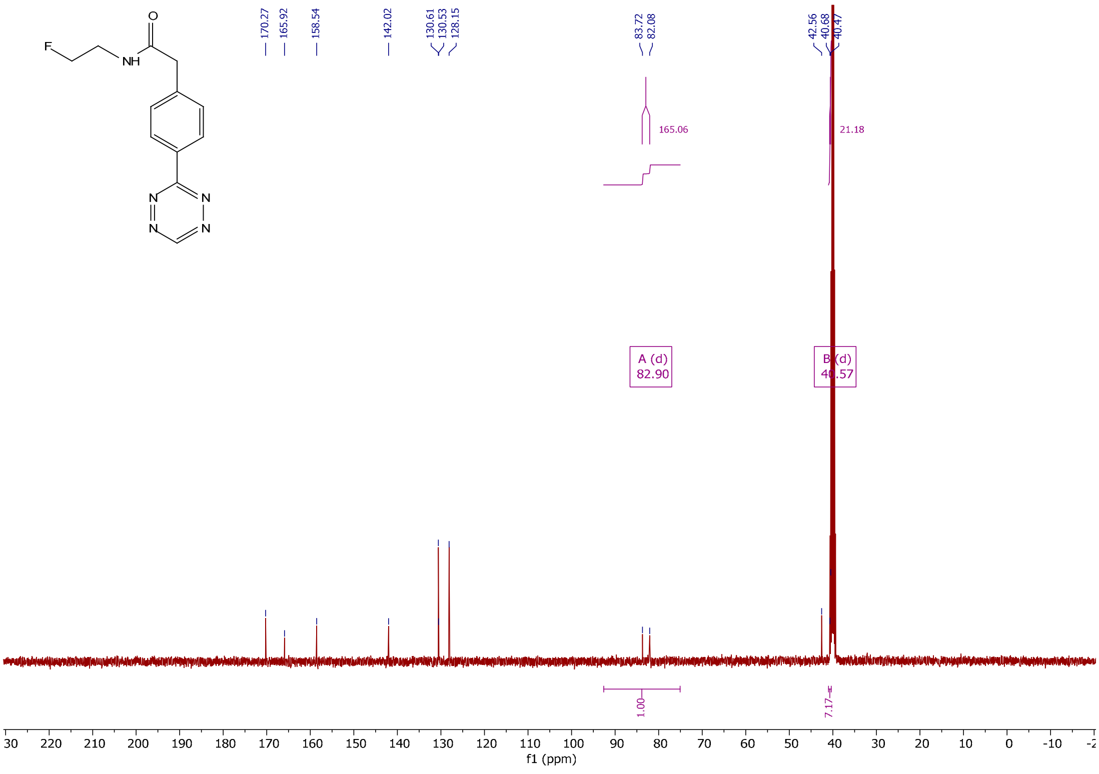

*2-(2-(4-(1,2,4,5-Tetrazin-3-yl)phenyl)acetamido)ethyl 4-nitrobenzenesulfonate (**6a**)*

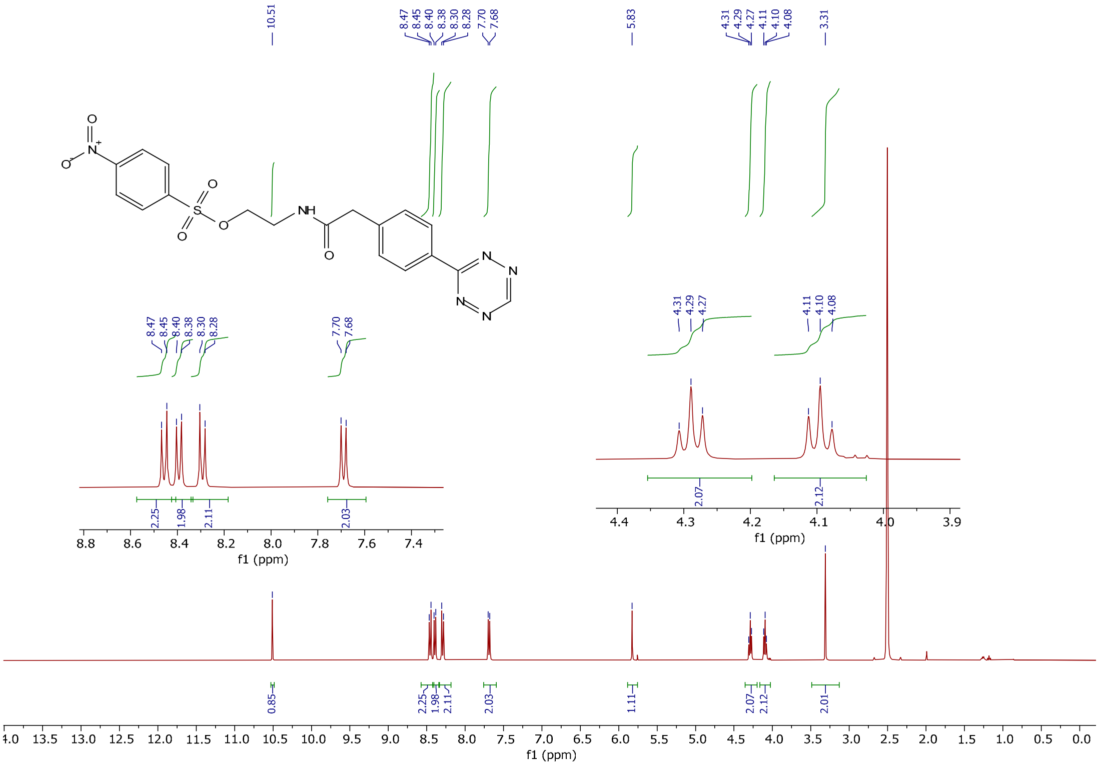

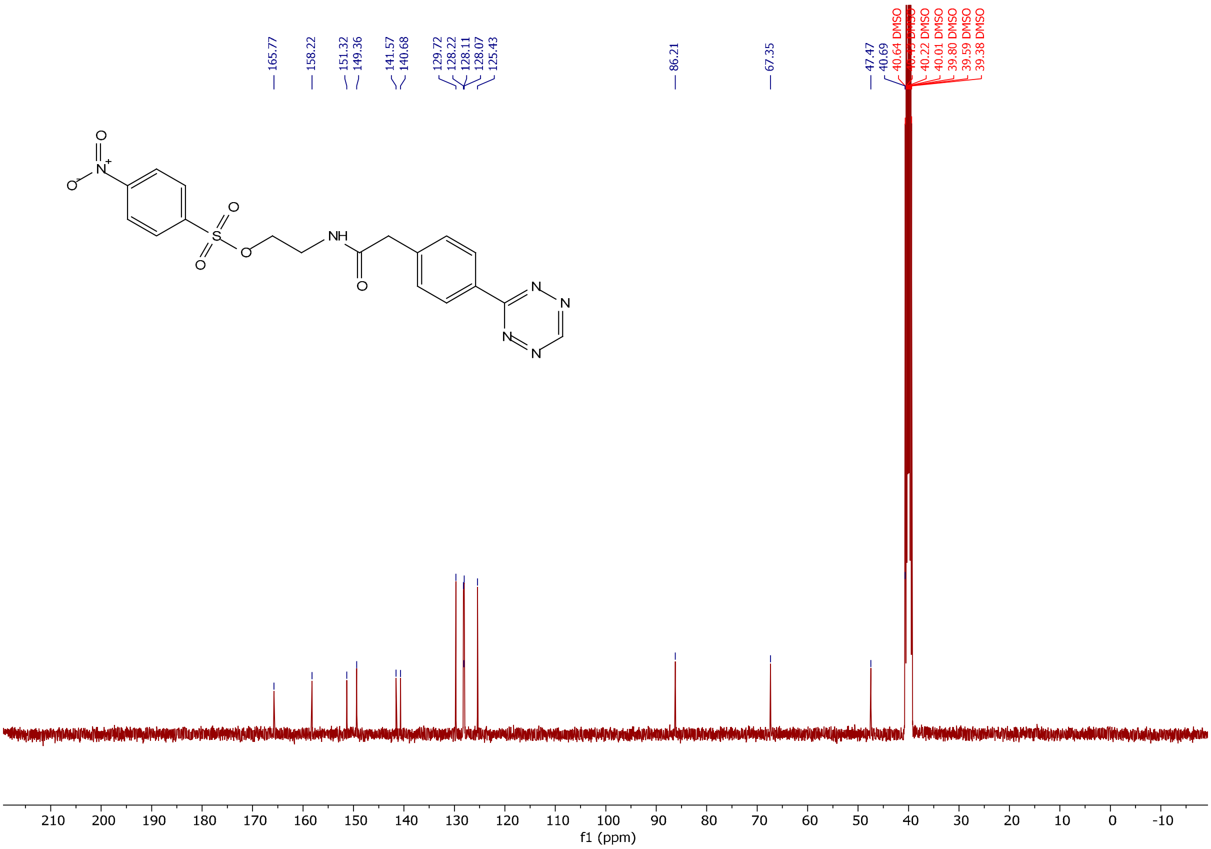

3-(4-(2-Fluoroethoxy)phenyl)-1,2,4,5-tetrazine (7)

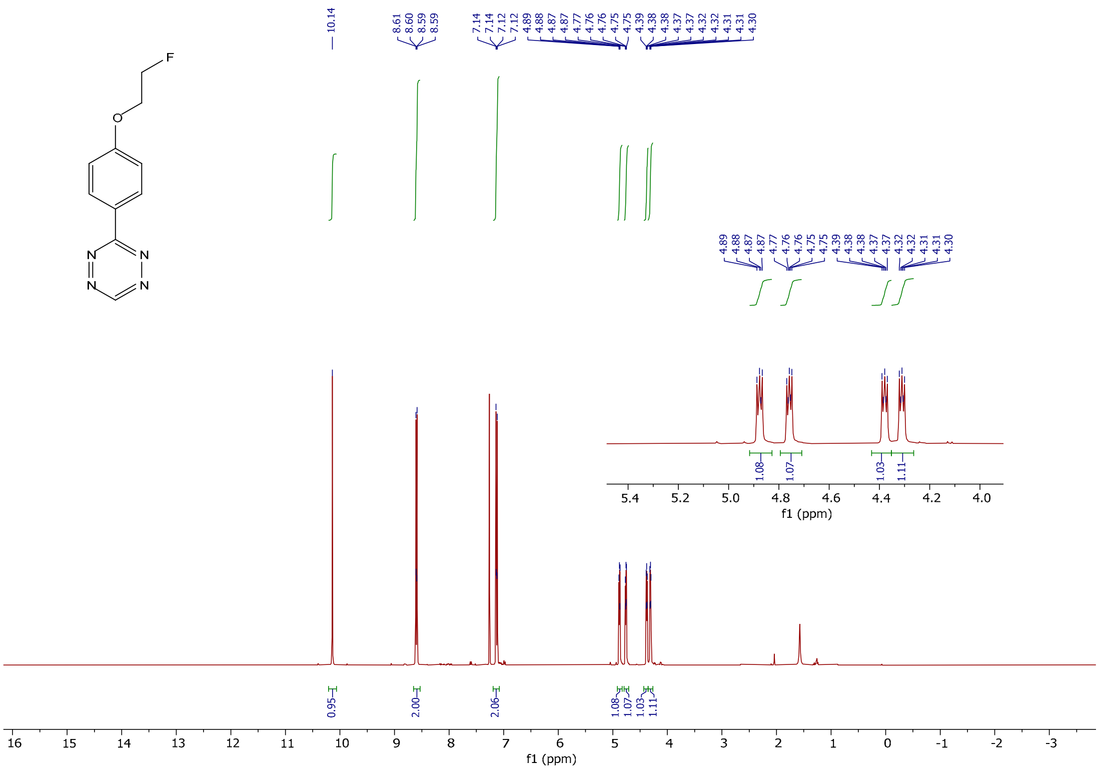

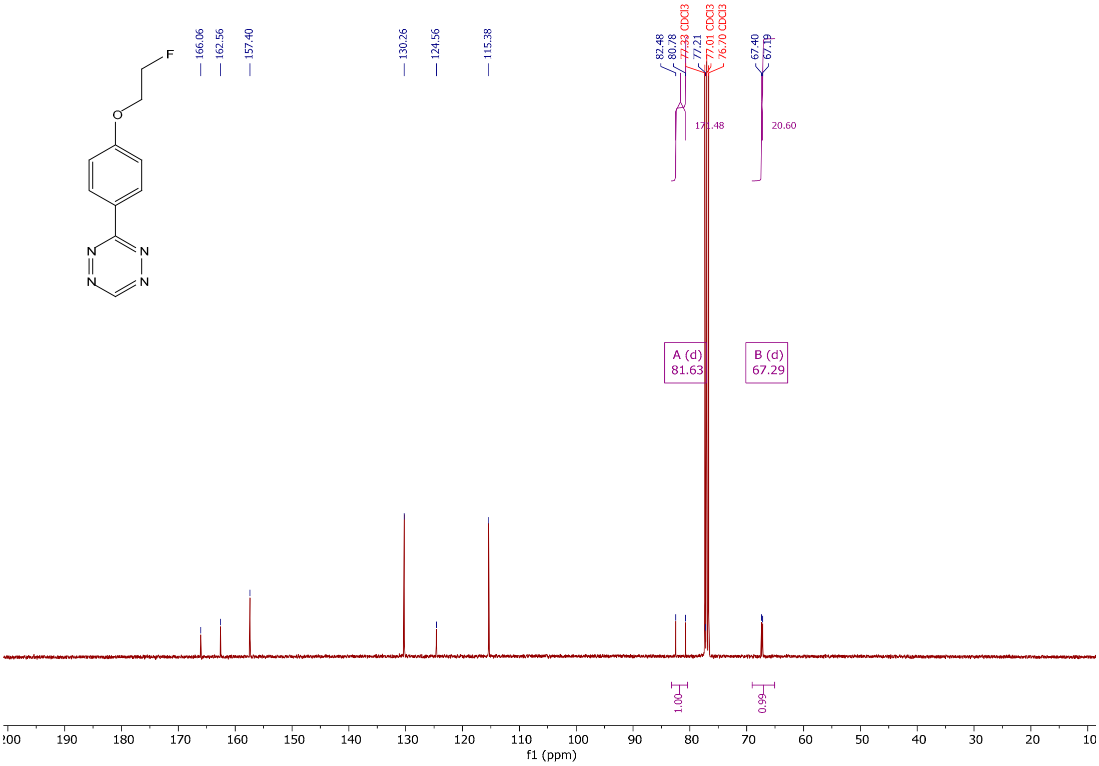

*2-(4-(1,2,4,5-Tetrazin-3-yl)phenoxy)ethyl 4-nitrobenzenesulfonate (7**a**)*

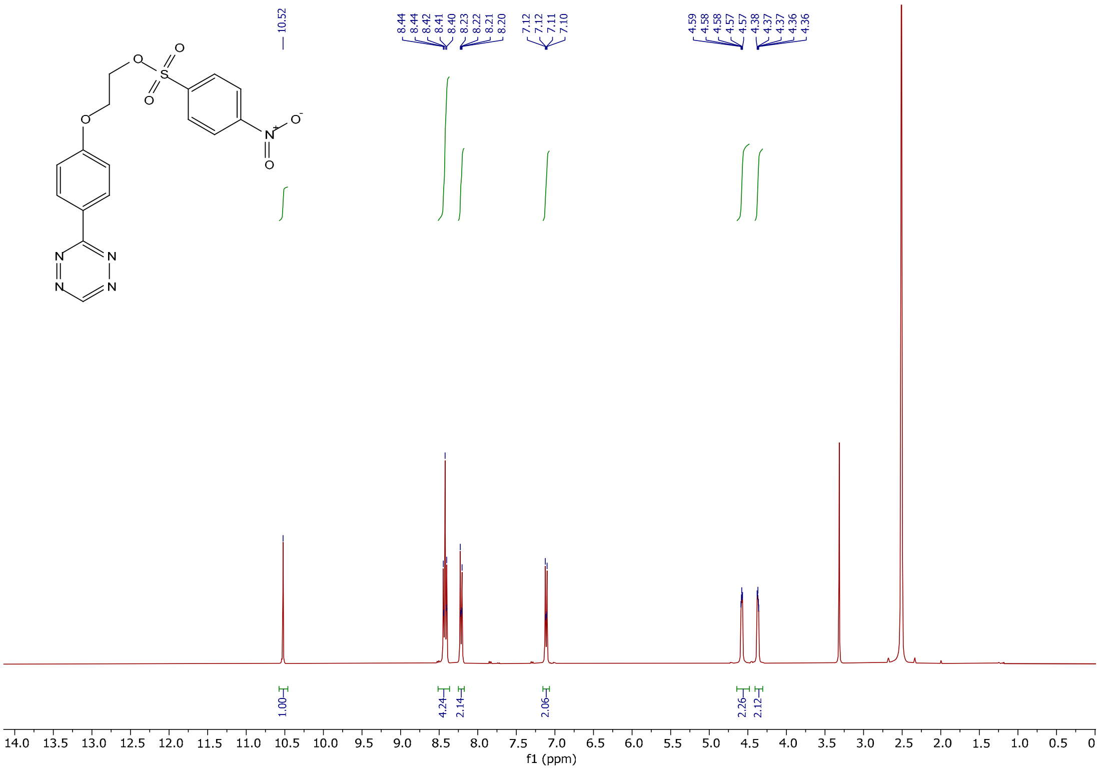

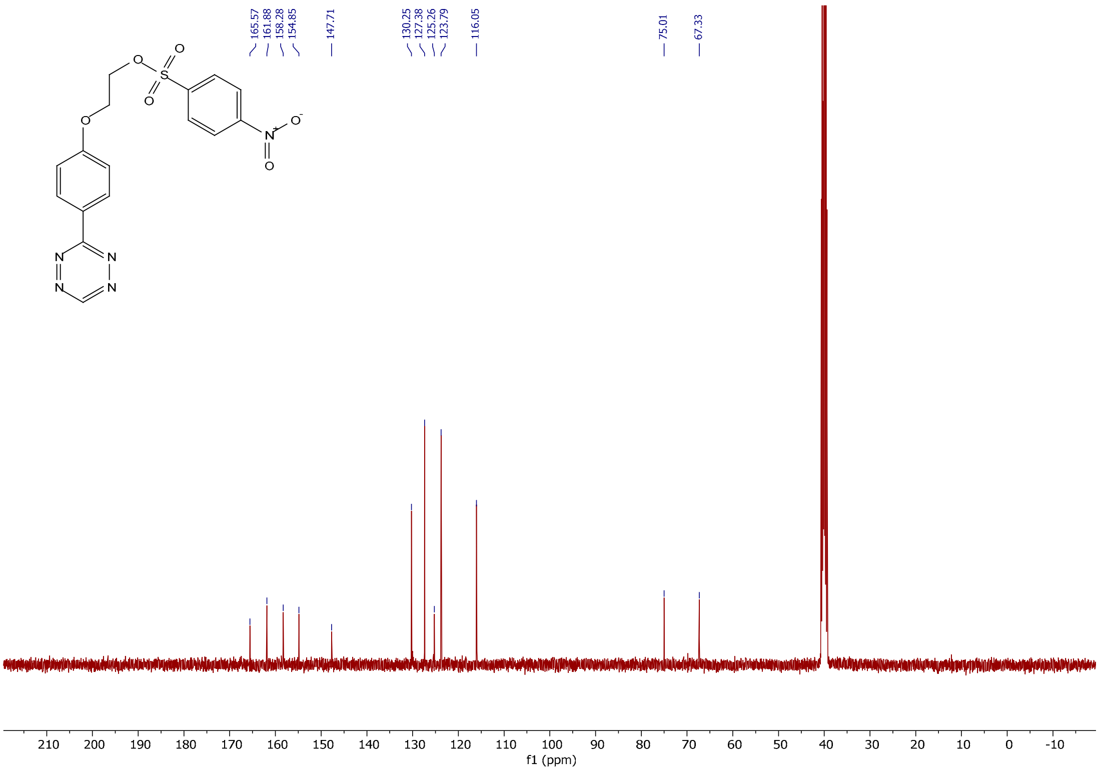

*N-(4-(1,2,4,5-tetrazin-3-yl)benzyl)-2-fluoroethan-1-amine (**9**)*

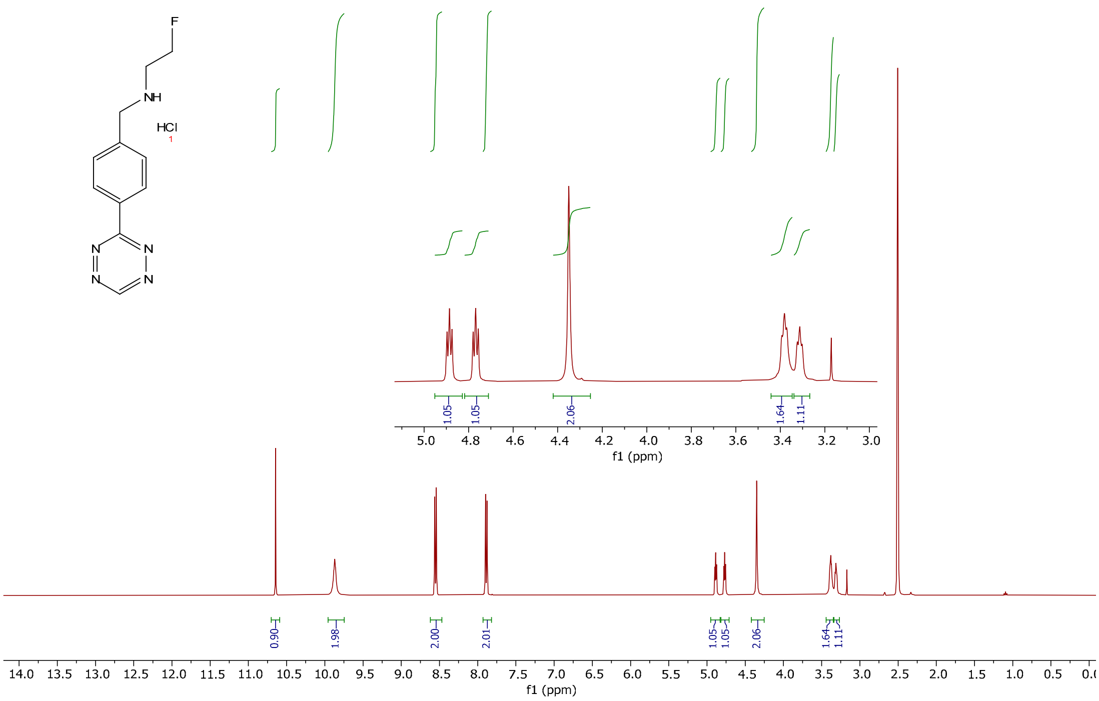

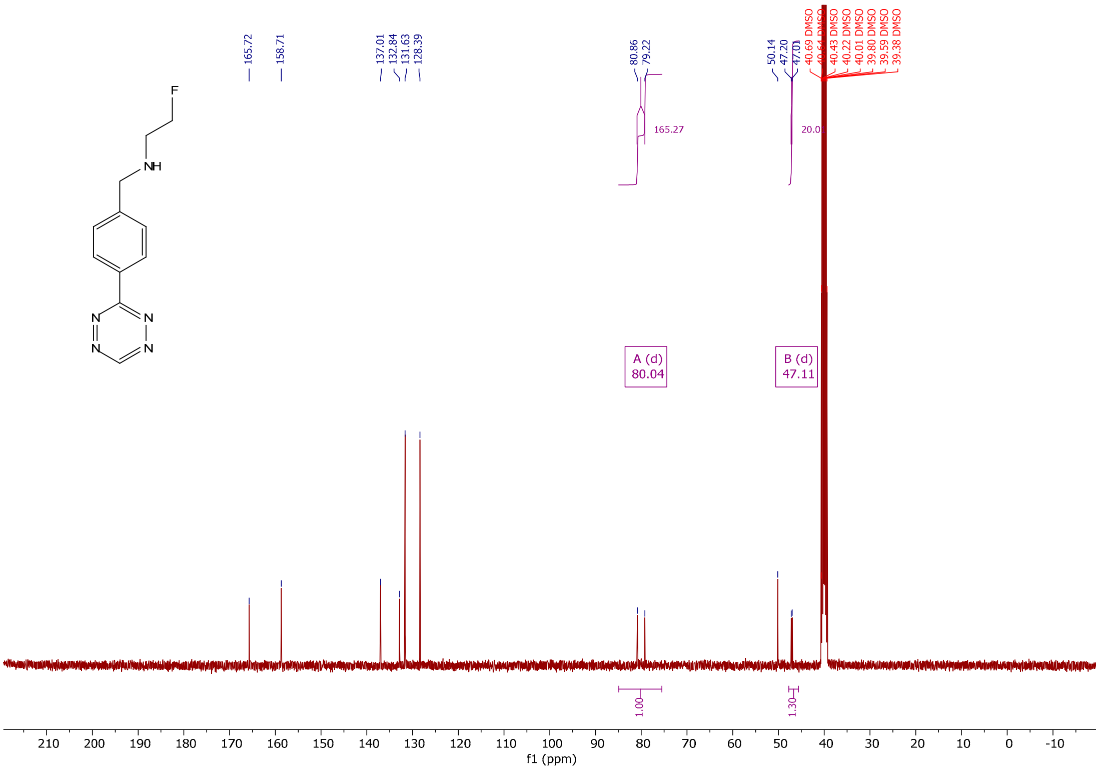

*2-((4-(1,2,4,5-Tetrazin-3-yl)benzyl)(trityl)amino)ethyl 4-nitrobenzenesulfonate (**9b**)*

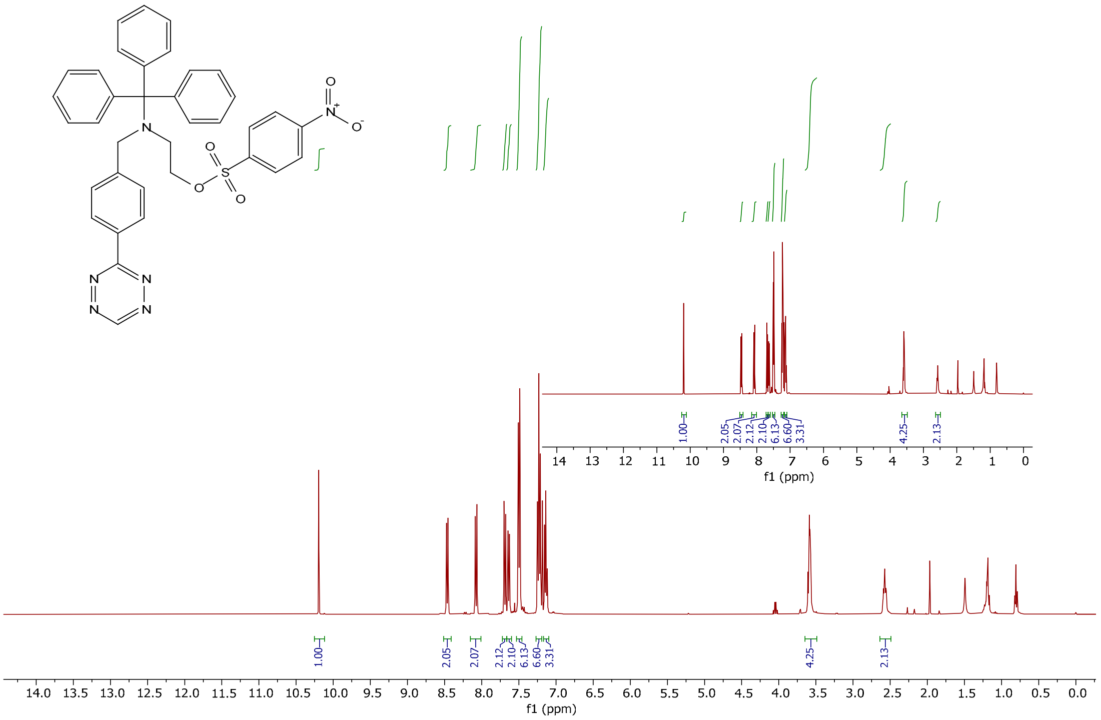

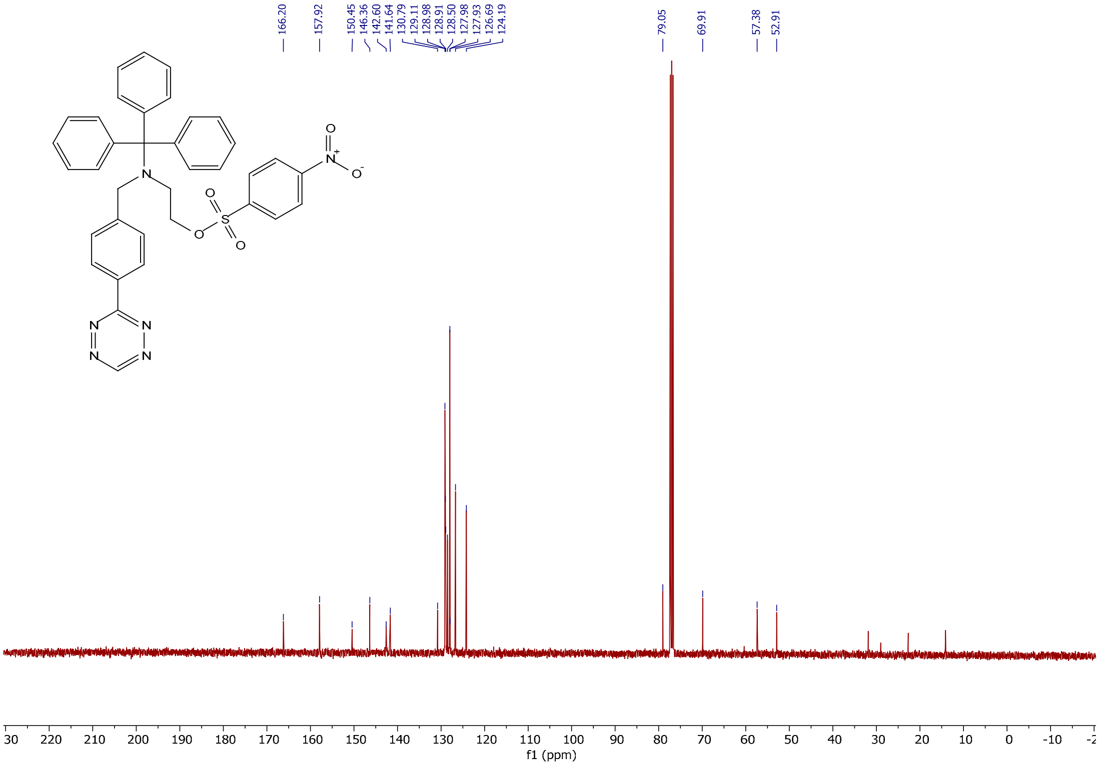

*4-(1,2,4,5-tetrazin-3-yl)benzoic acid (**10**)*

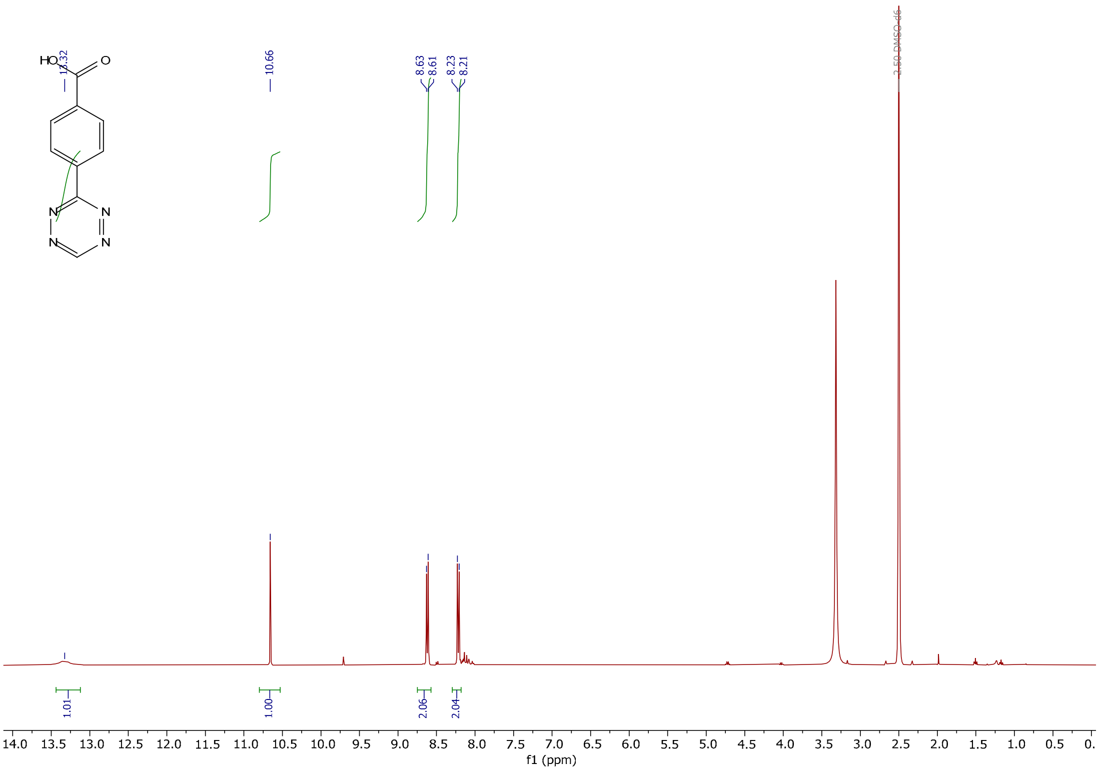

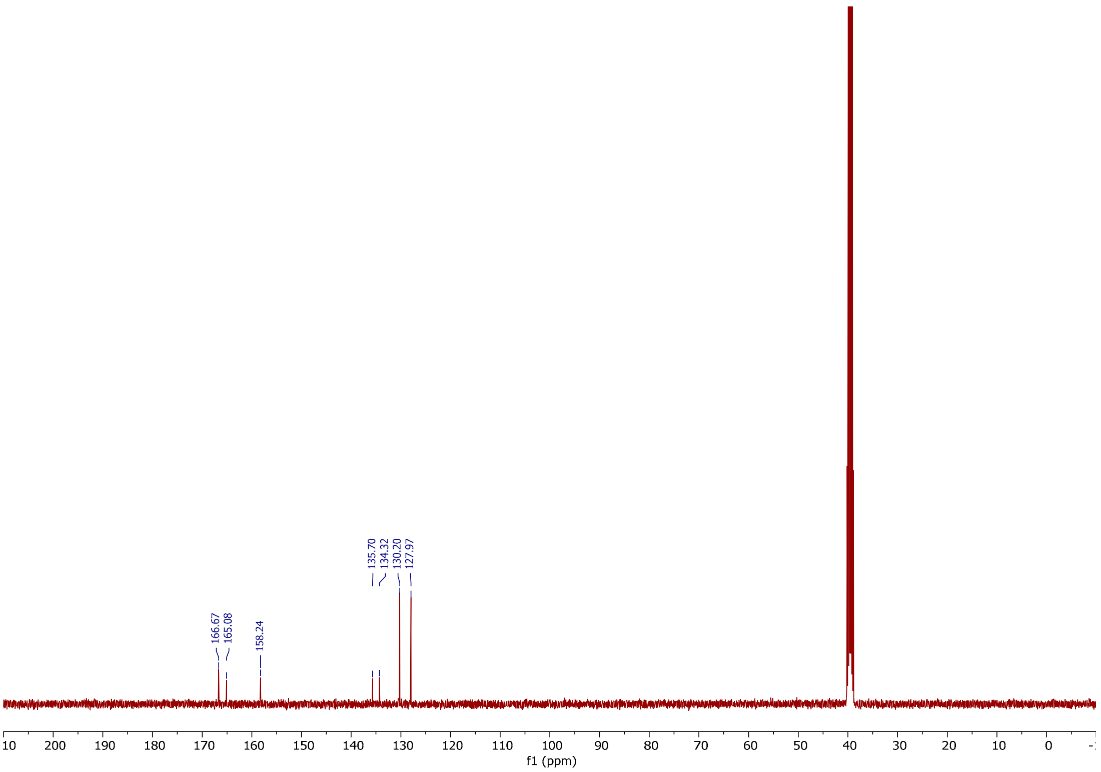

2-Hydroxyethyl 4-(1,2,4,5-tetrazin-3-yl)benzoate ***(11)***

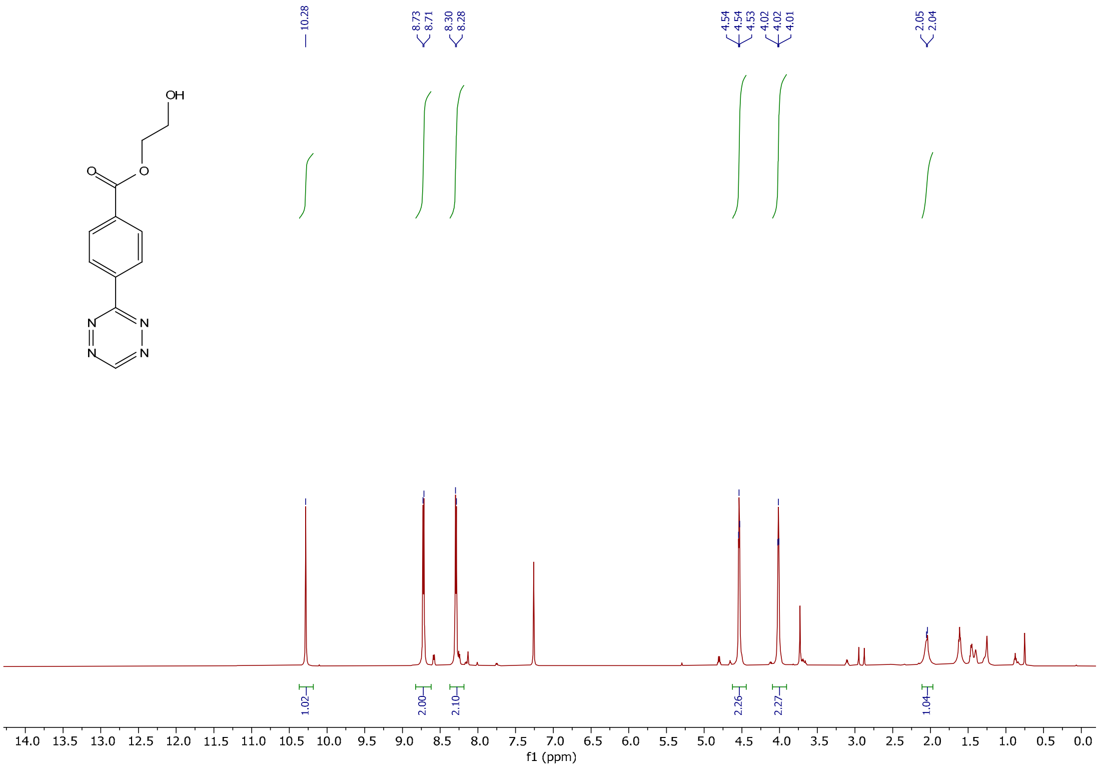

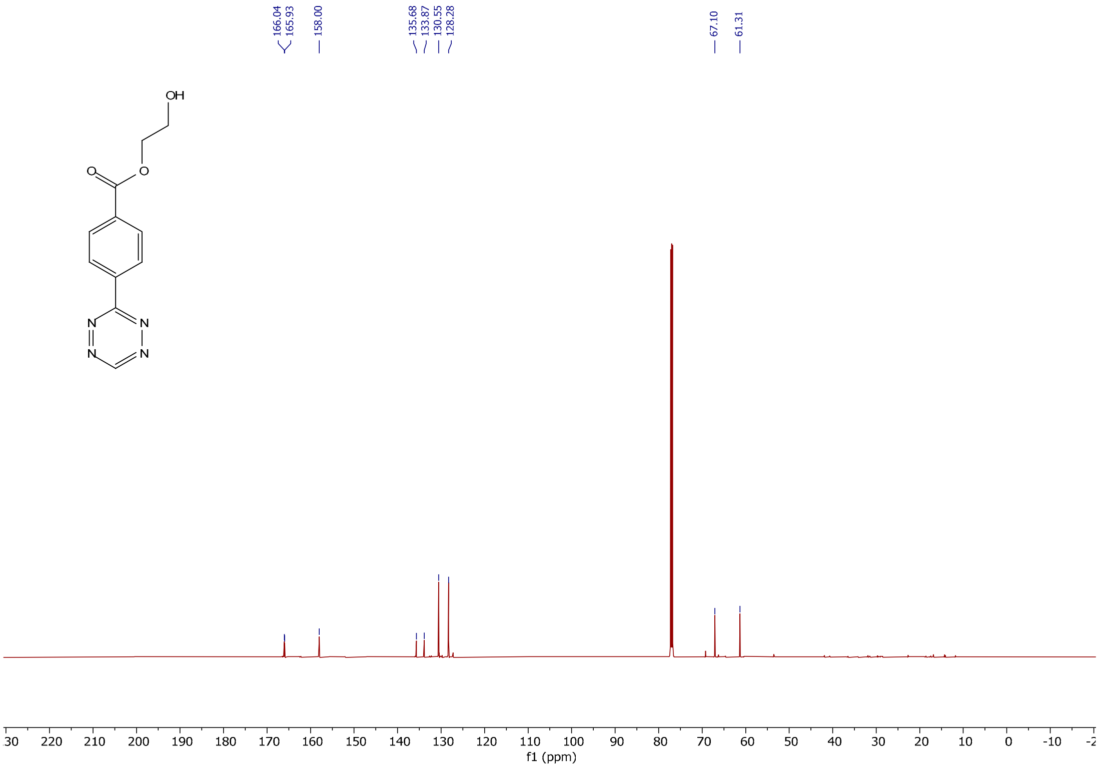

*4-Cyano-N-(2-fluoroethyl)benzamide (**12**)*

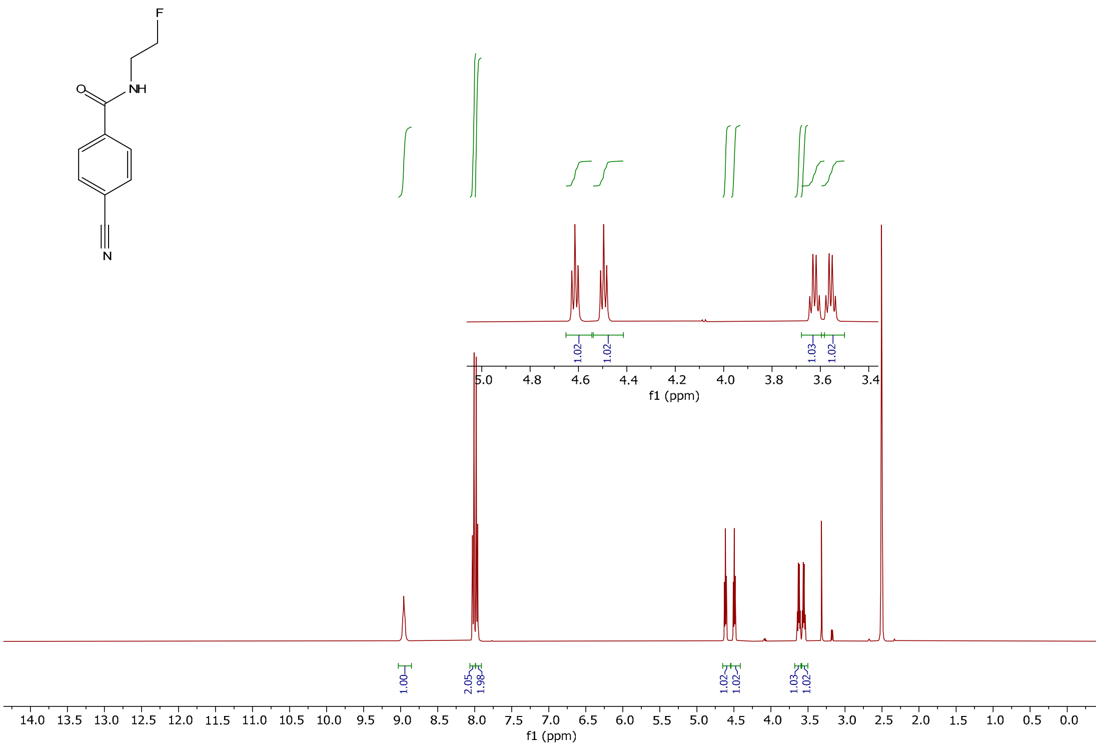

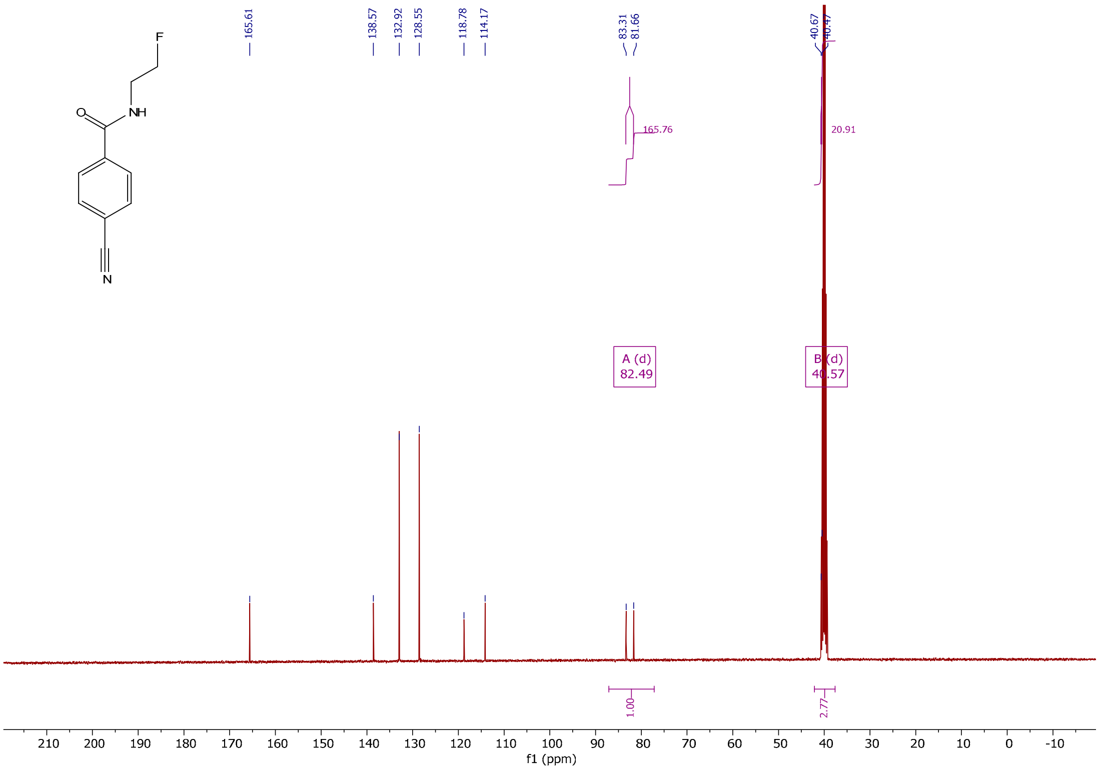

*4-Cyano-N-(2-hydroxyethyl)benzamide (**13**)*

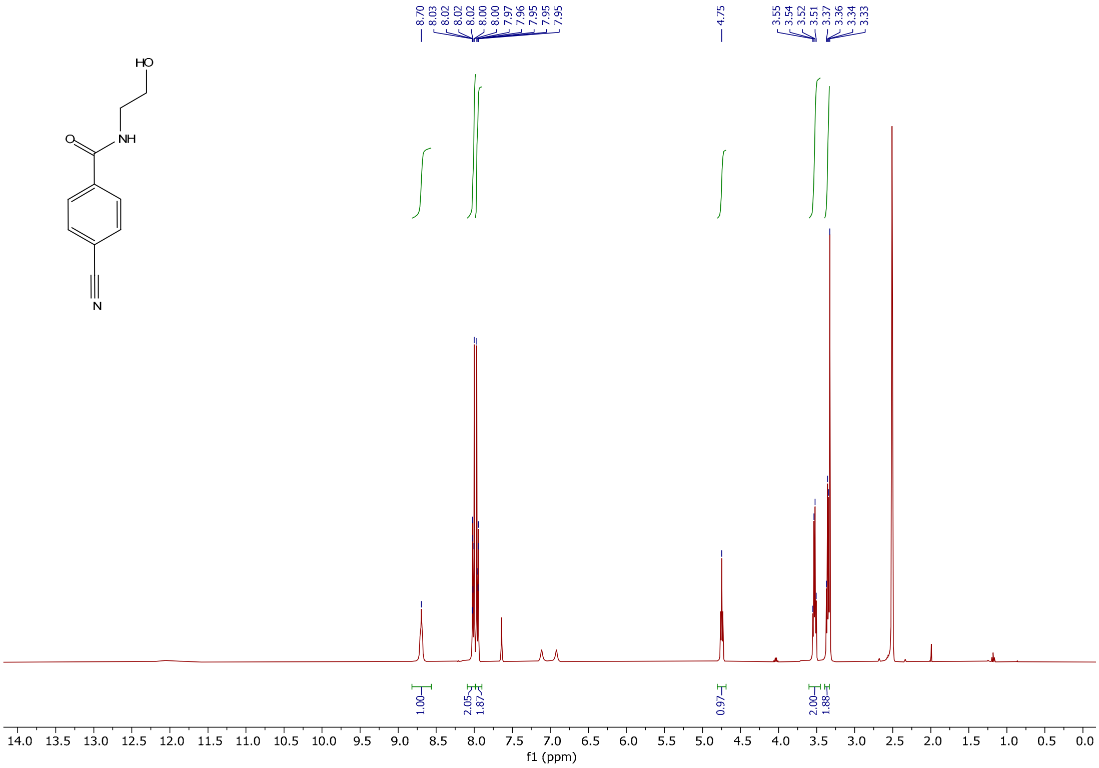

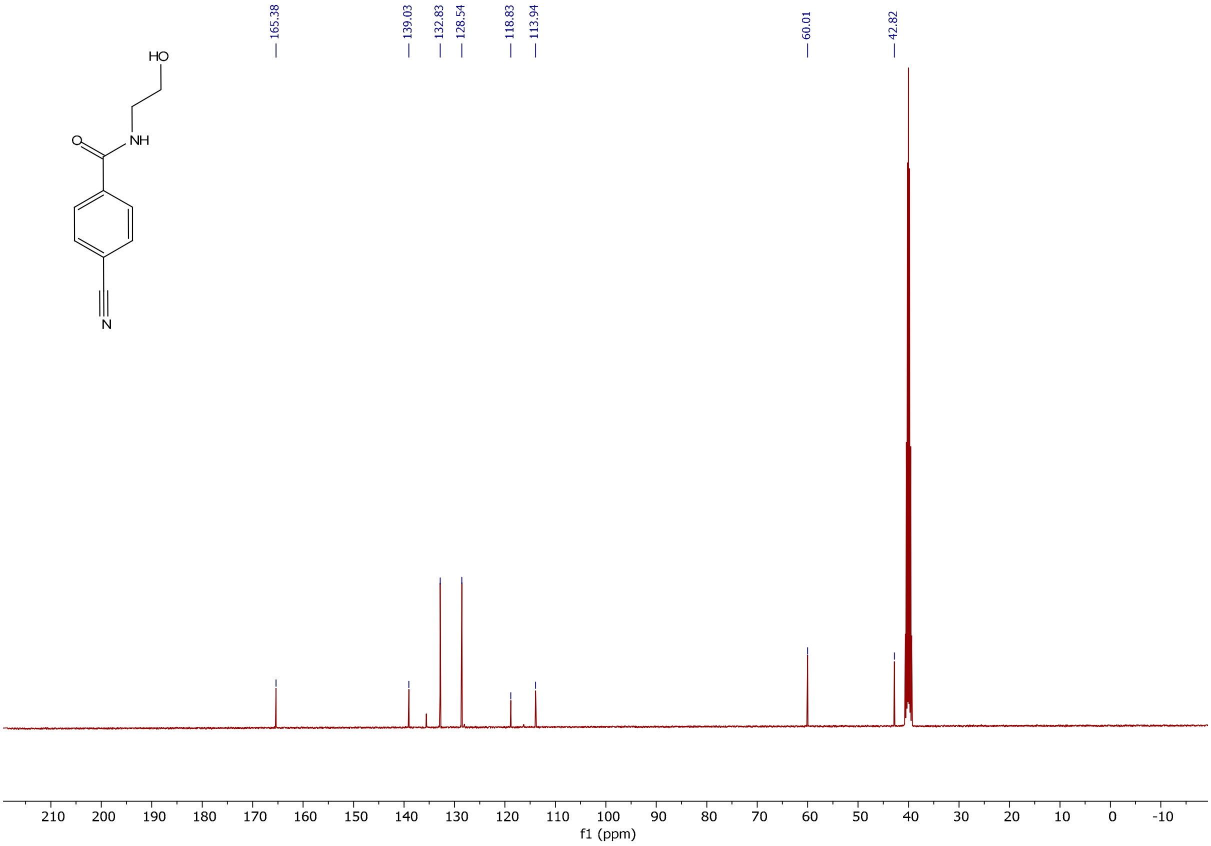

*N-(2-hydroxyethyl)-4-(1,2,4,5-tetrazin-3-yl)benzamide (**14**)*

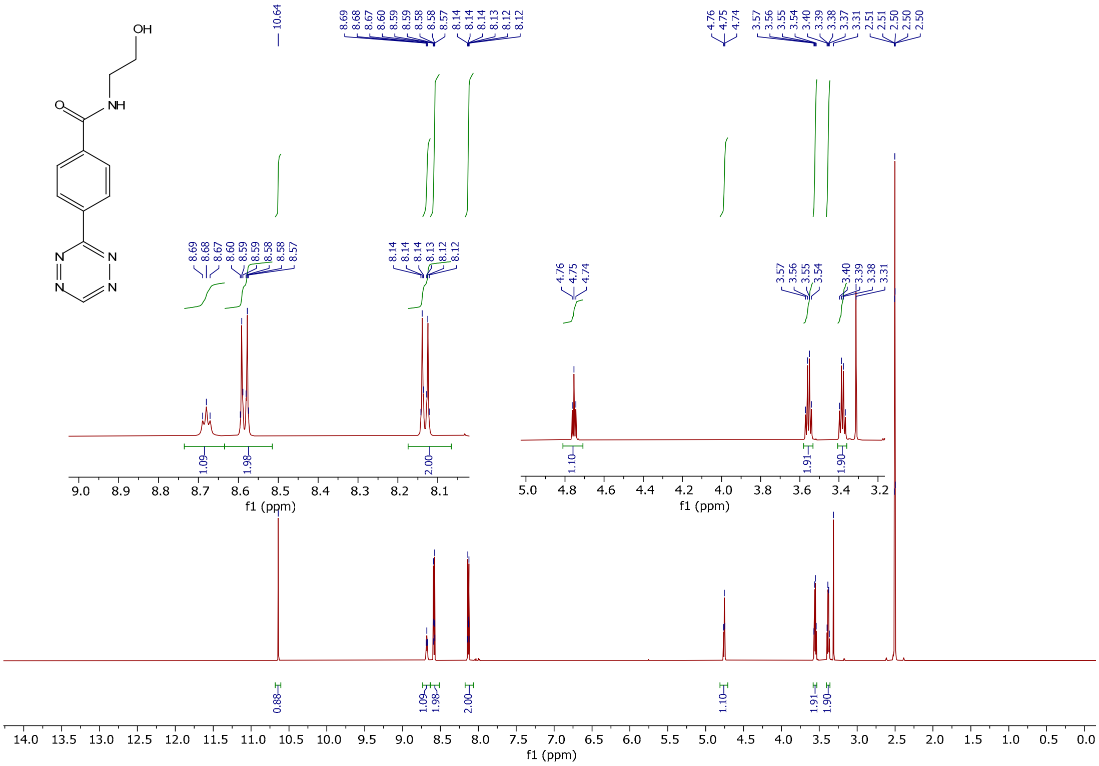

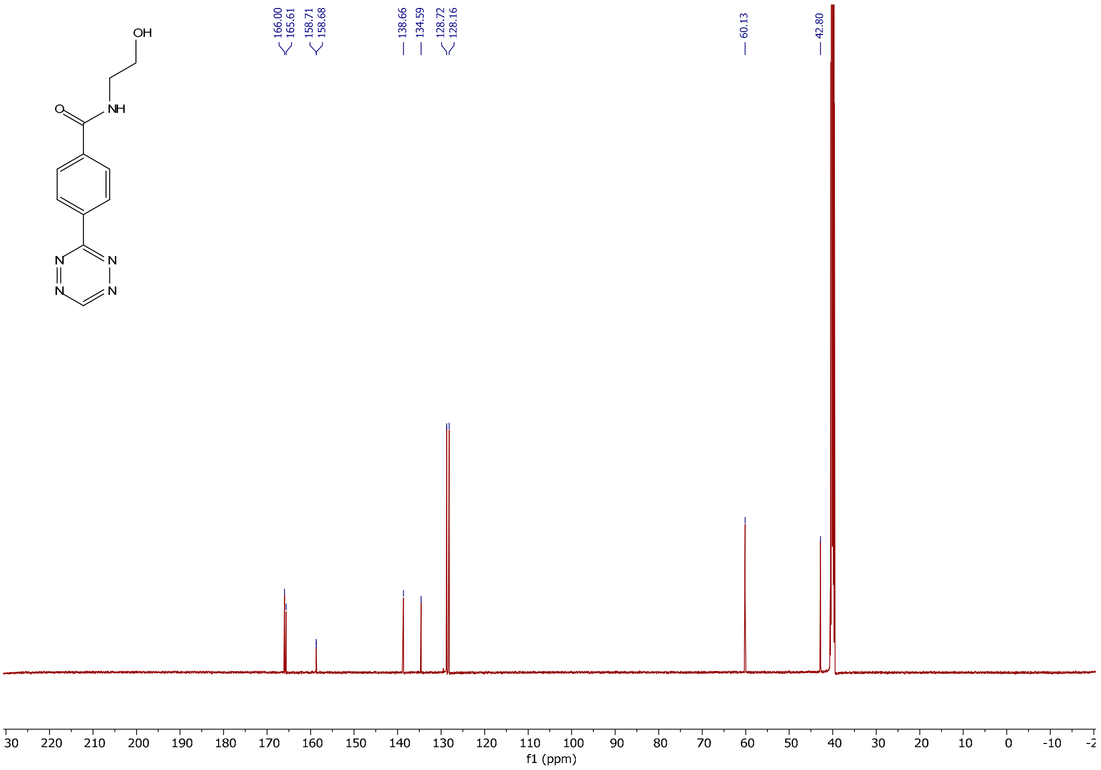

2-(4-(1,2,4,5-Tetrazin-3-yl)phenyl)-4,5-dihydrooxazole

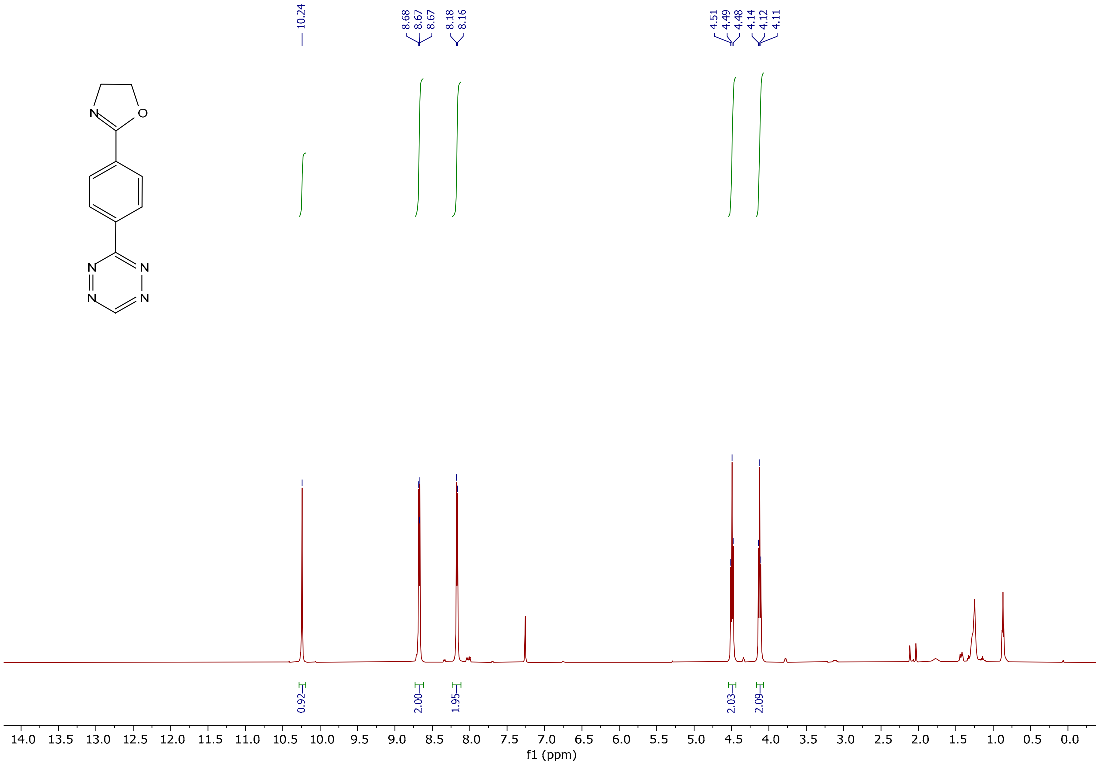

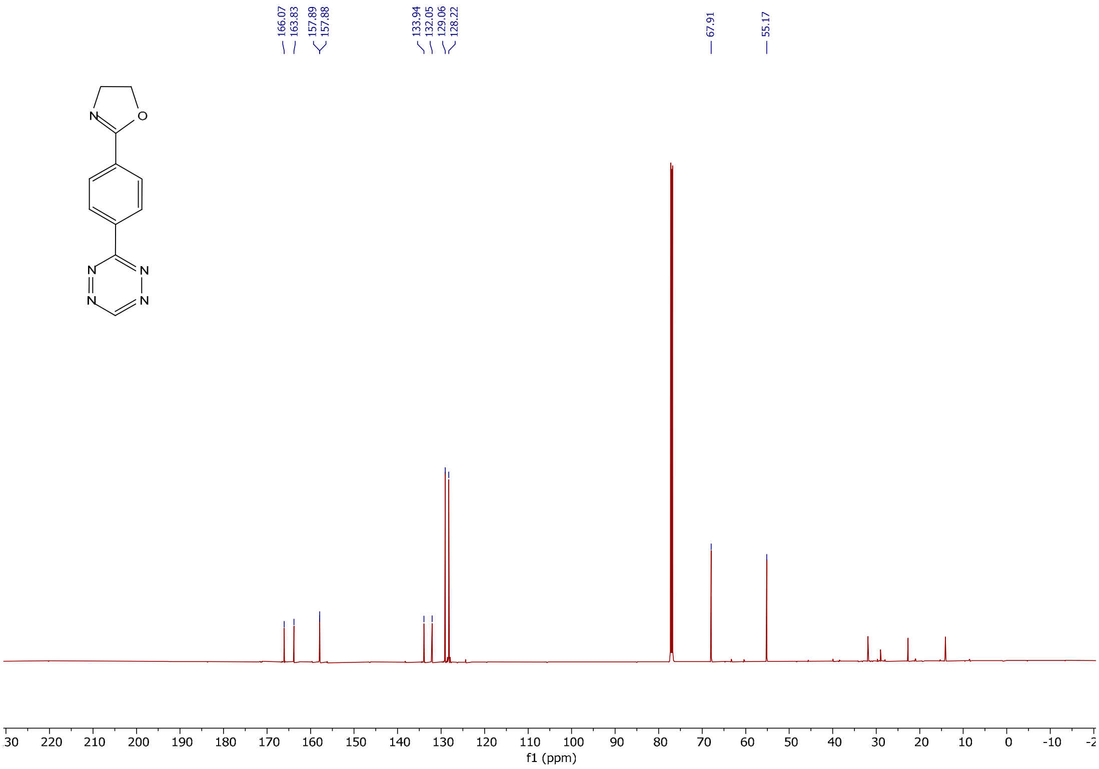

*2-(4-(1,2,4,5-Tetrazin-3-yl)phenyl)acetic acid (**15**)*

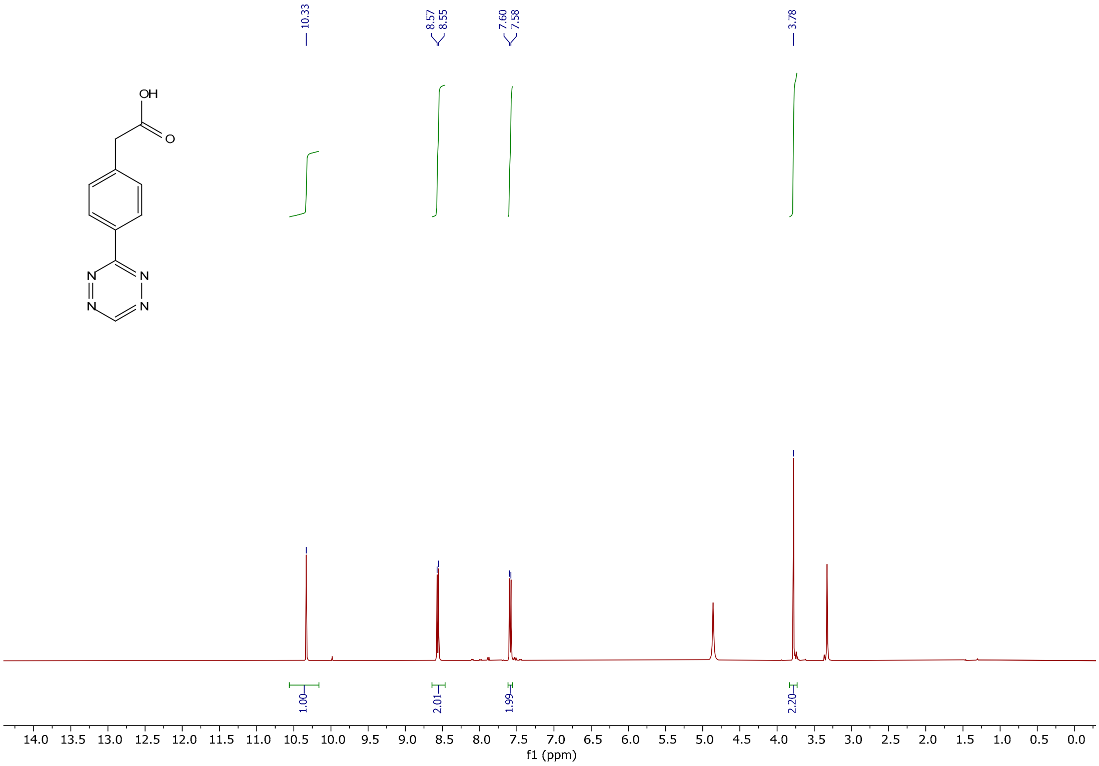

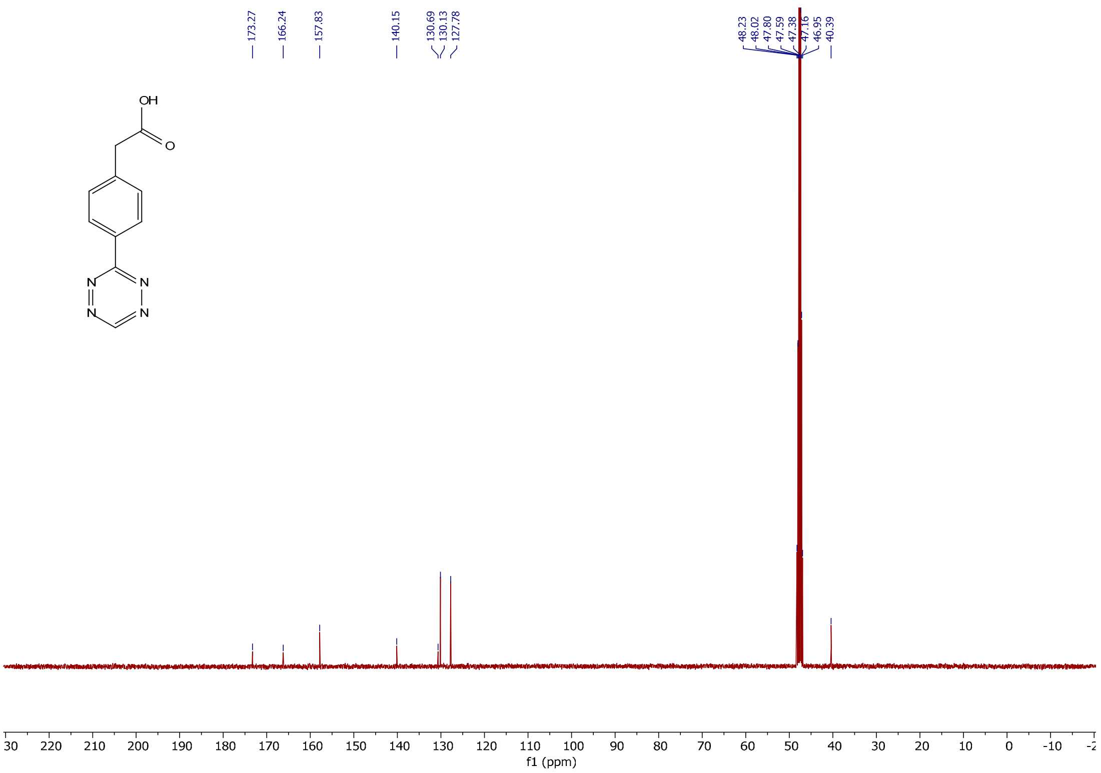

2-Hydroxyethyl 2-(4-(1,2,4,5-tetrazin-3-yl)phenyl)acetate (**16**)

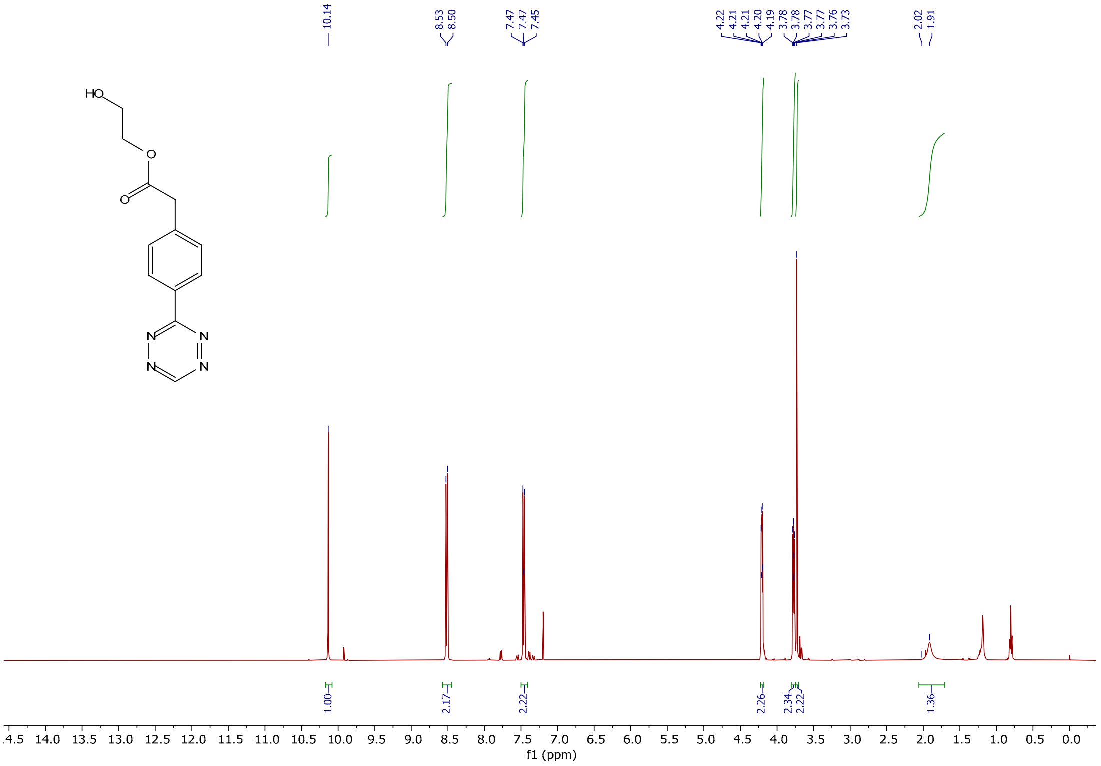

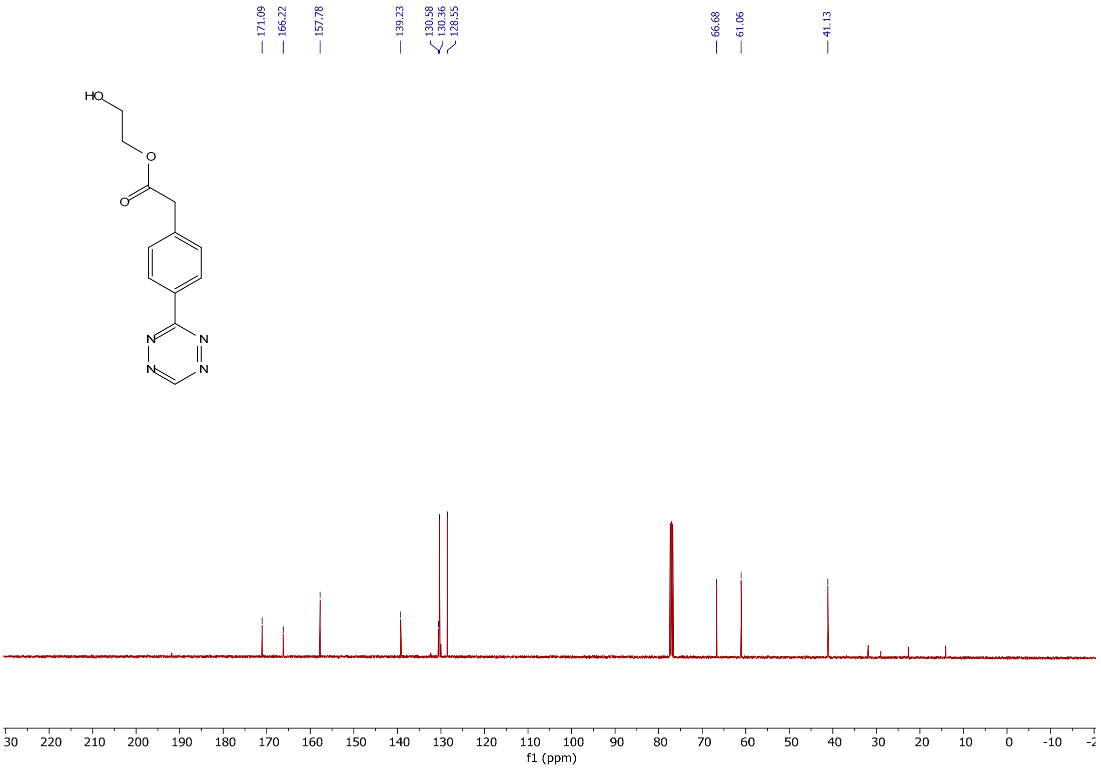

2-(4-Cyanophenyl)-N-(2-fluoroethyl)acetamide (**17**)

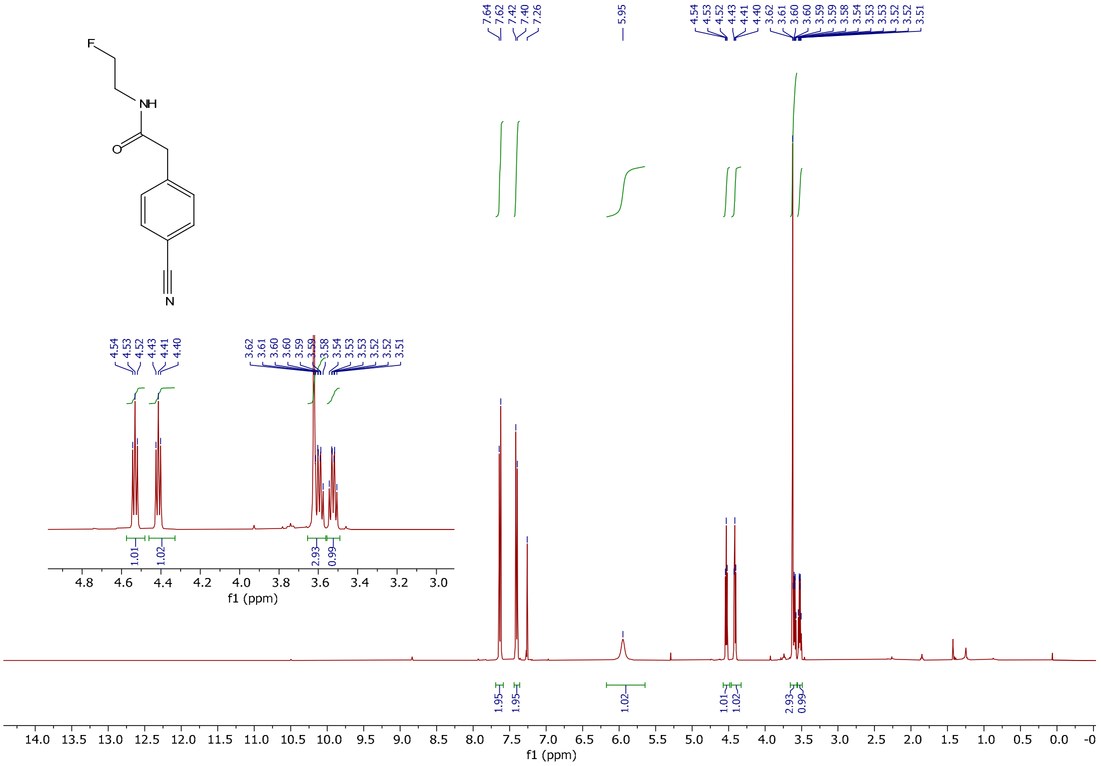

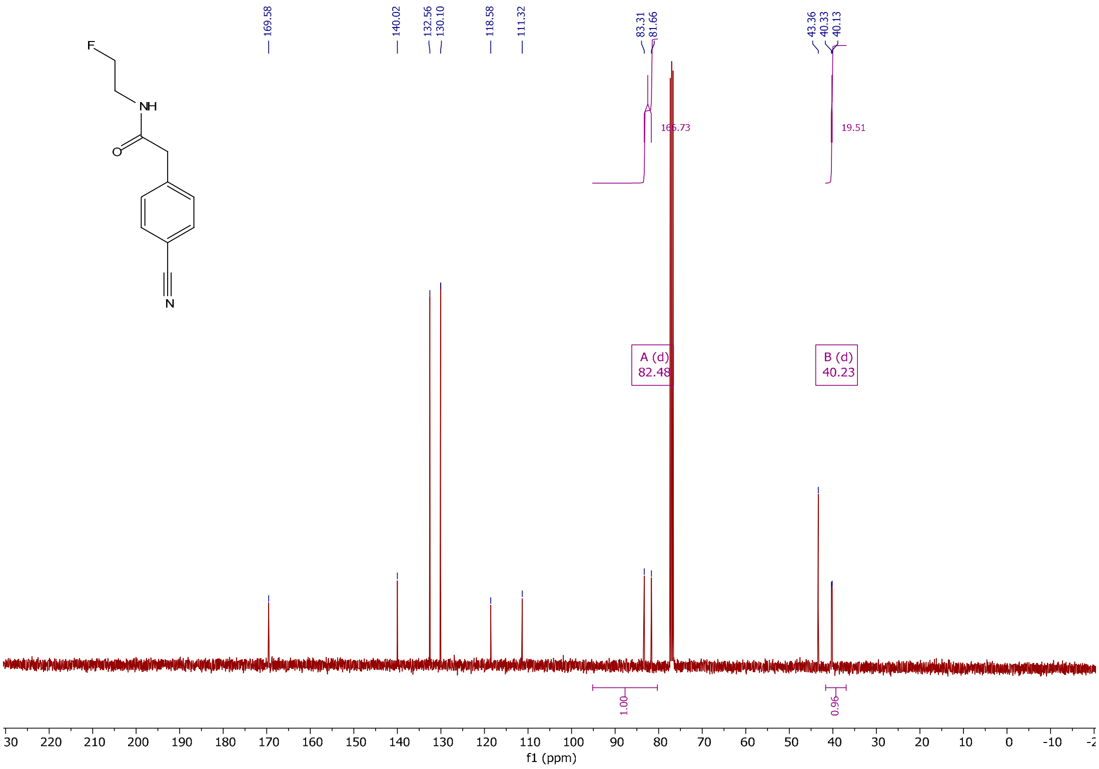

*2-(4-Cyanophenyl)-N-(2-hydroxyethyl)acetamide (**18**)*

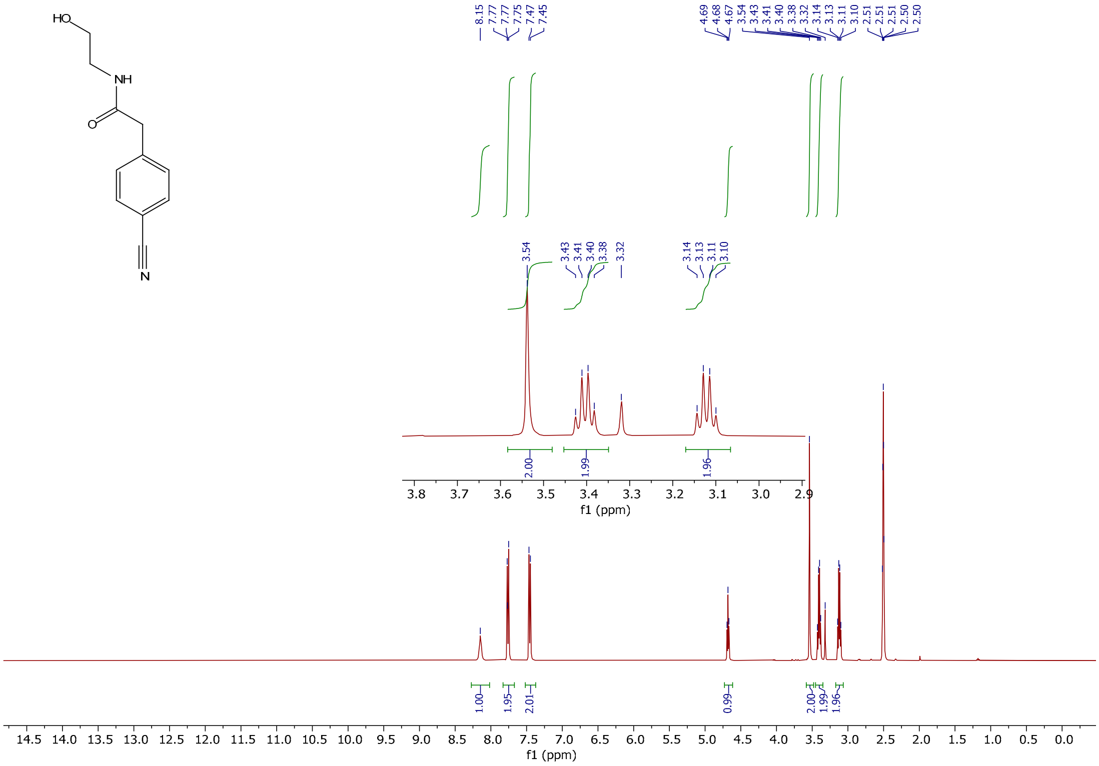

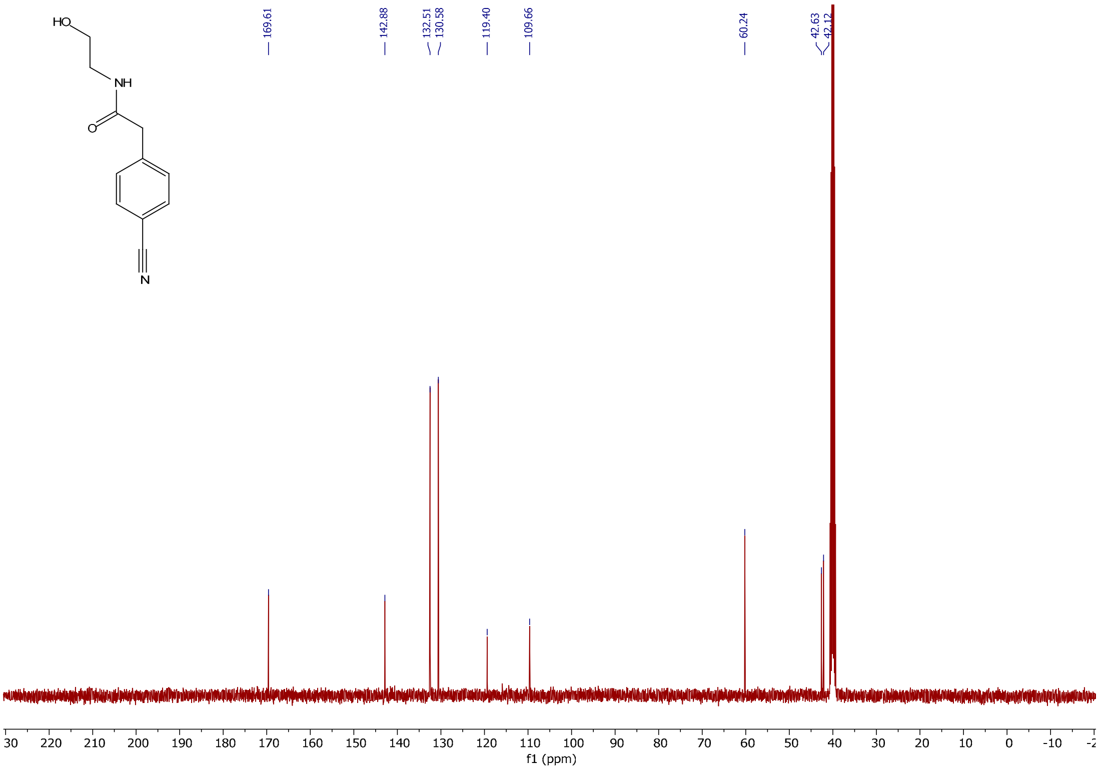

*2-(4-(1,2,4,5-Tetrazin-3-yl)phenyl)-N-(2-hydroxyethyl)acetamide (**19**)*

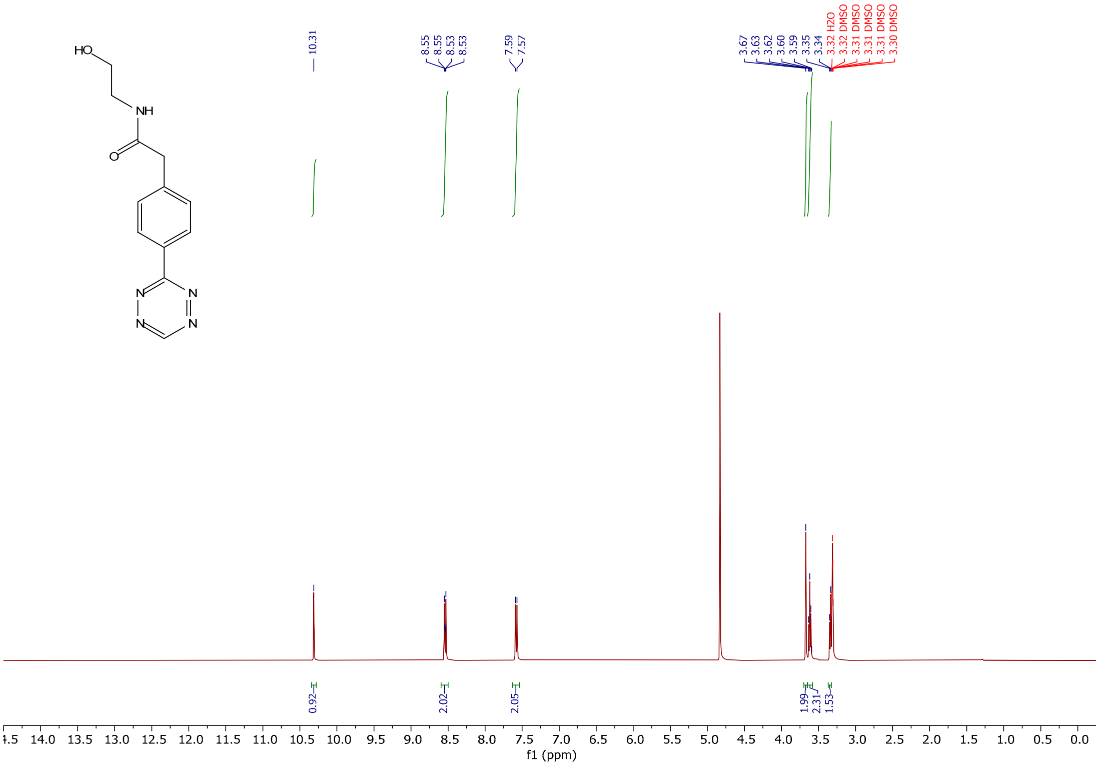

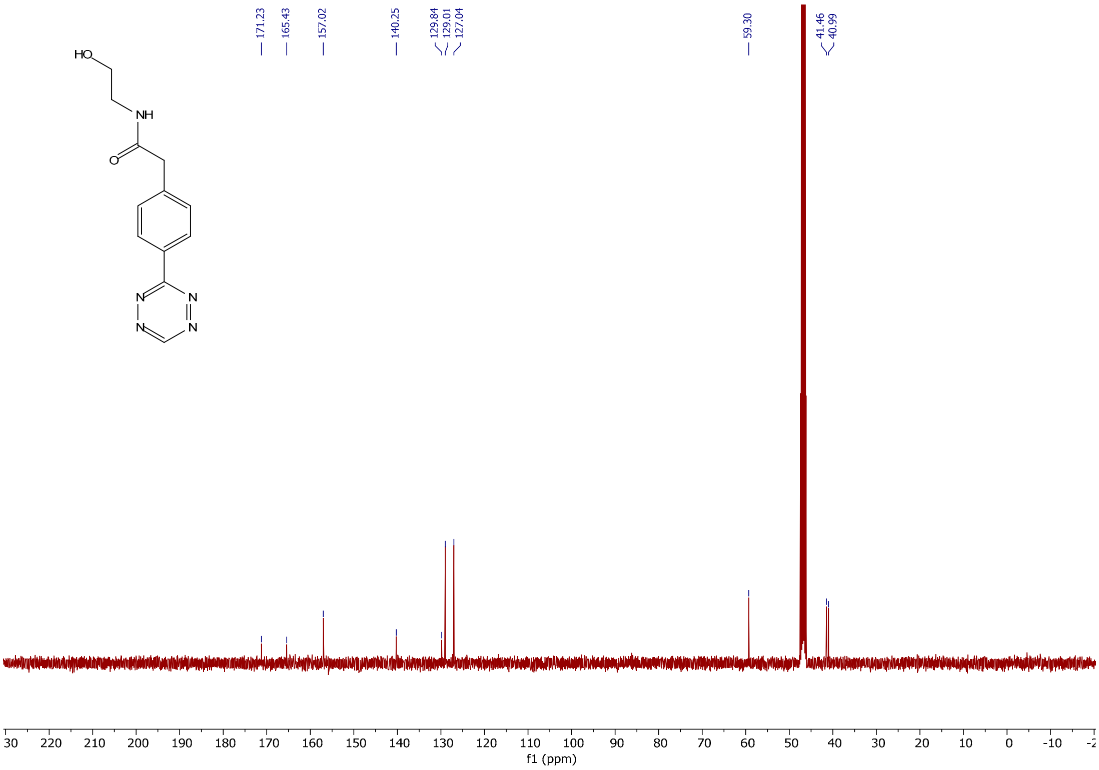

*4-(2-Fluoroethoxy)benzonitrile (**20**)*

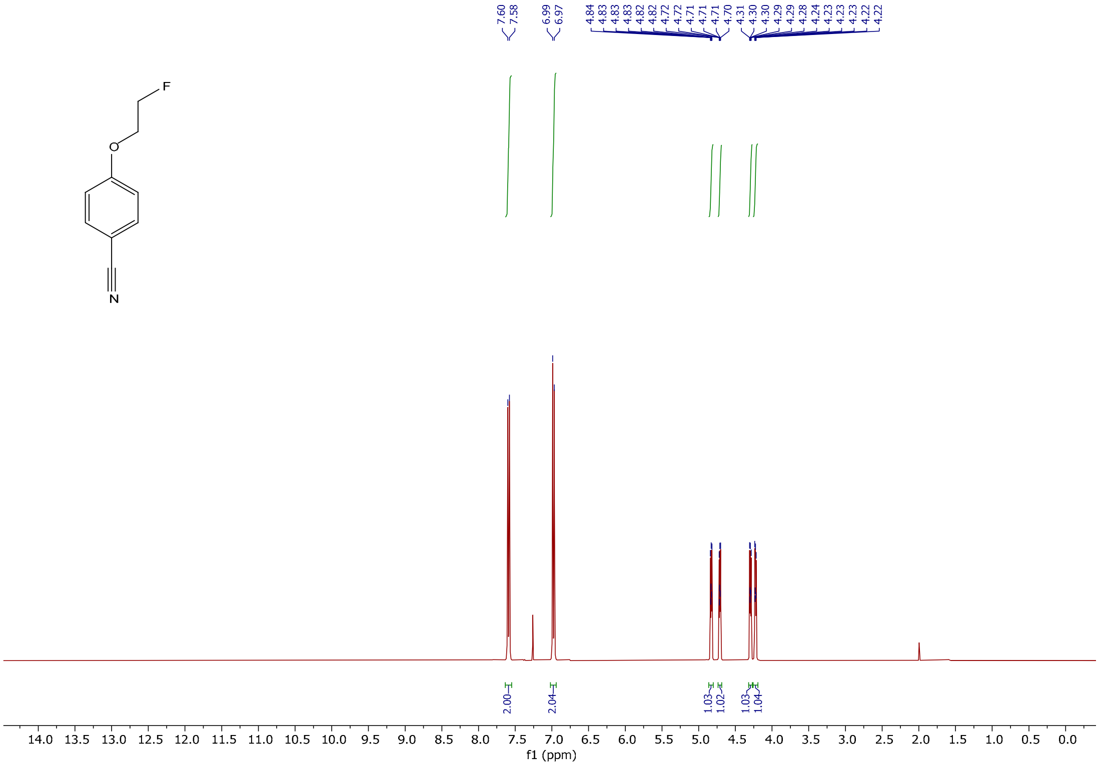

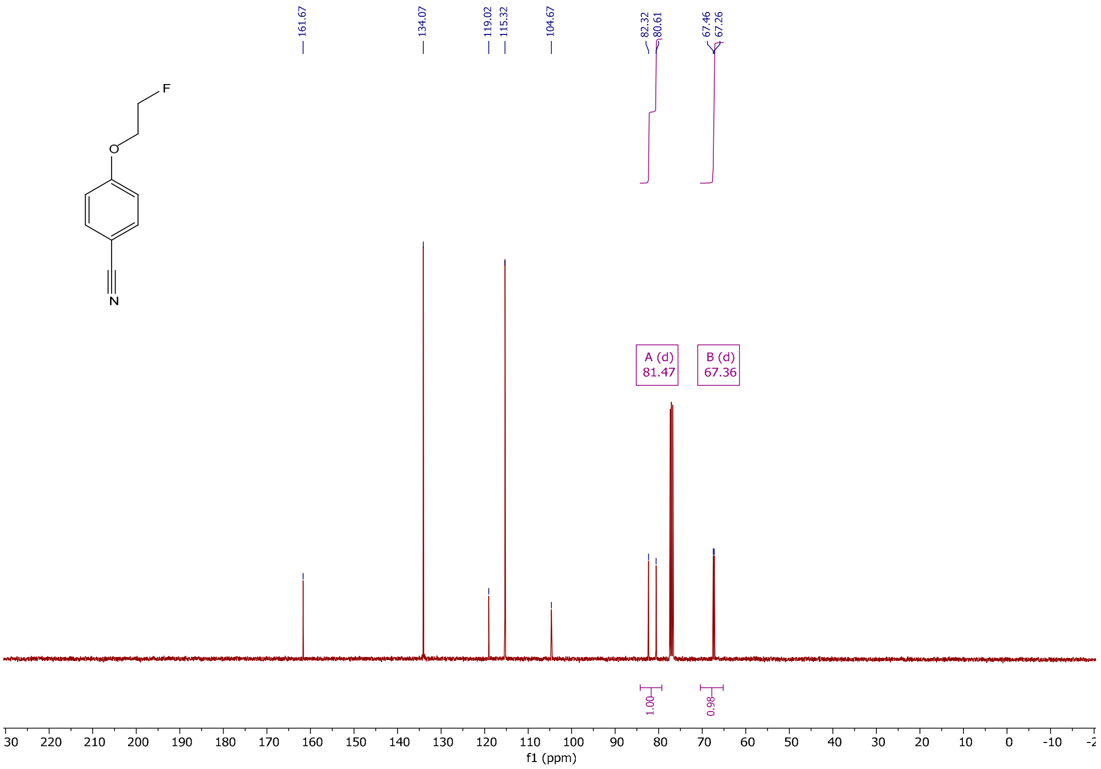

*4-(2-Hydroxyethoxy)benzonitrile (**21**)*

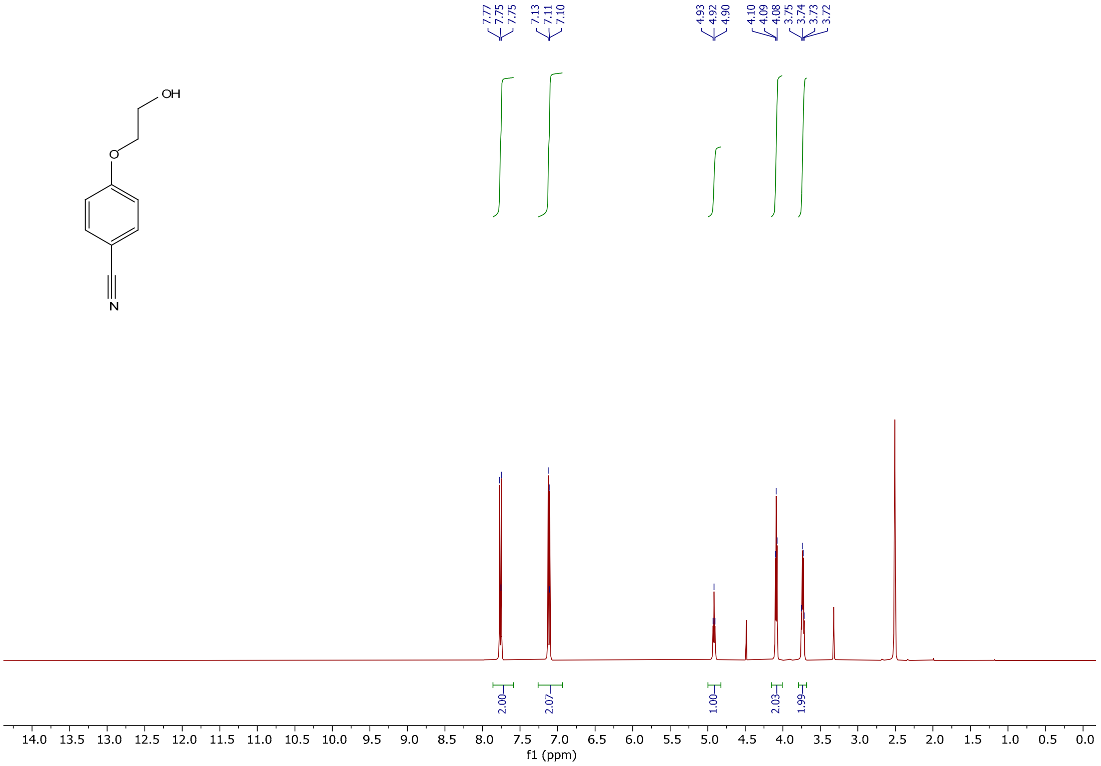

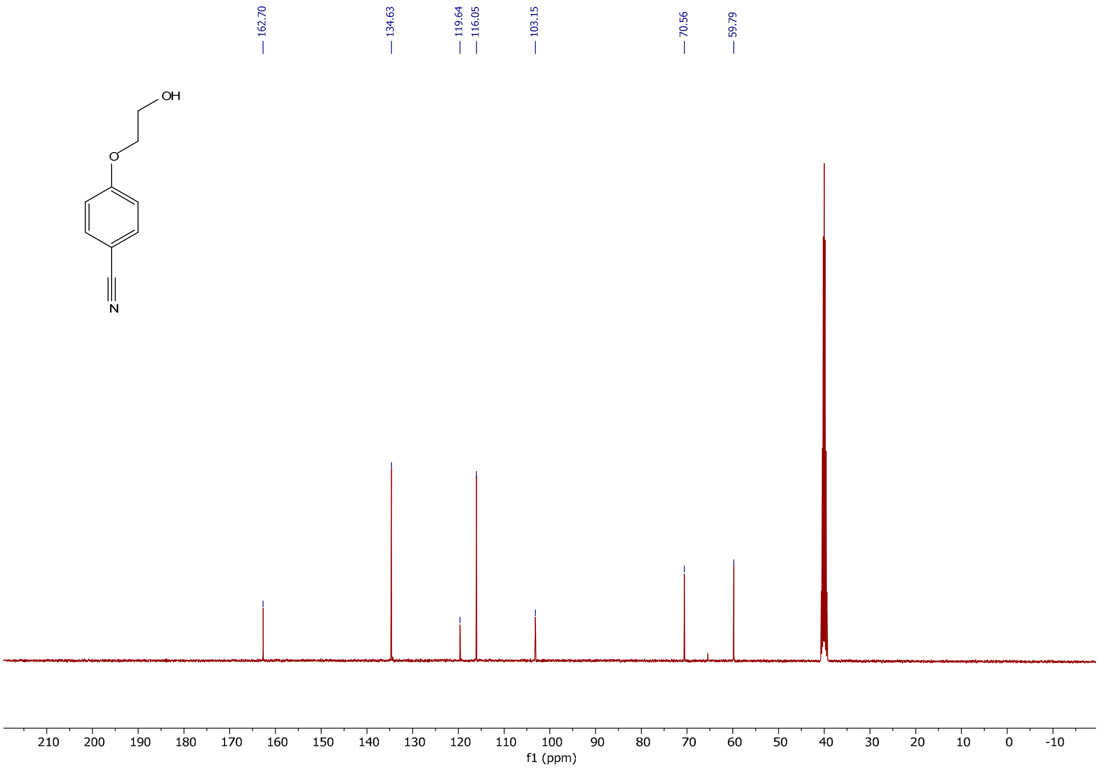

*2-(4-(1,2,4,5-Tetrazin-3-yl)phenoxy)ethan-1-ol (**22**)*

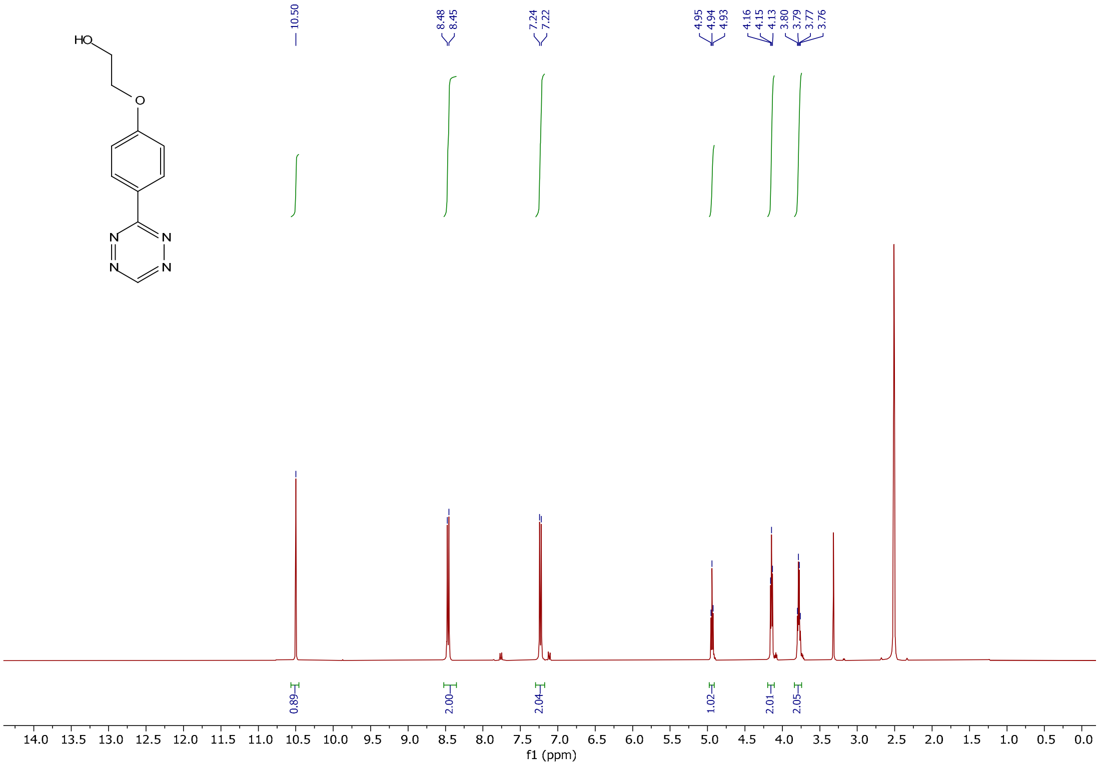

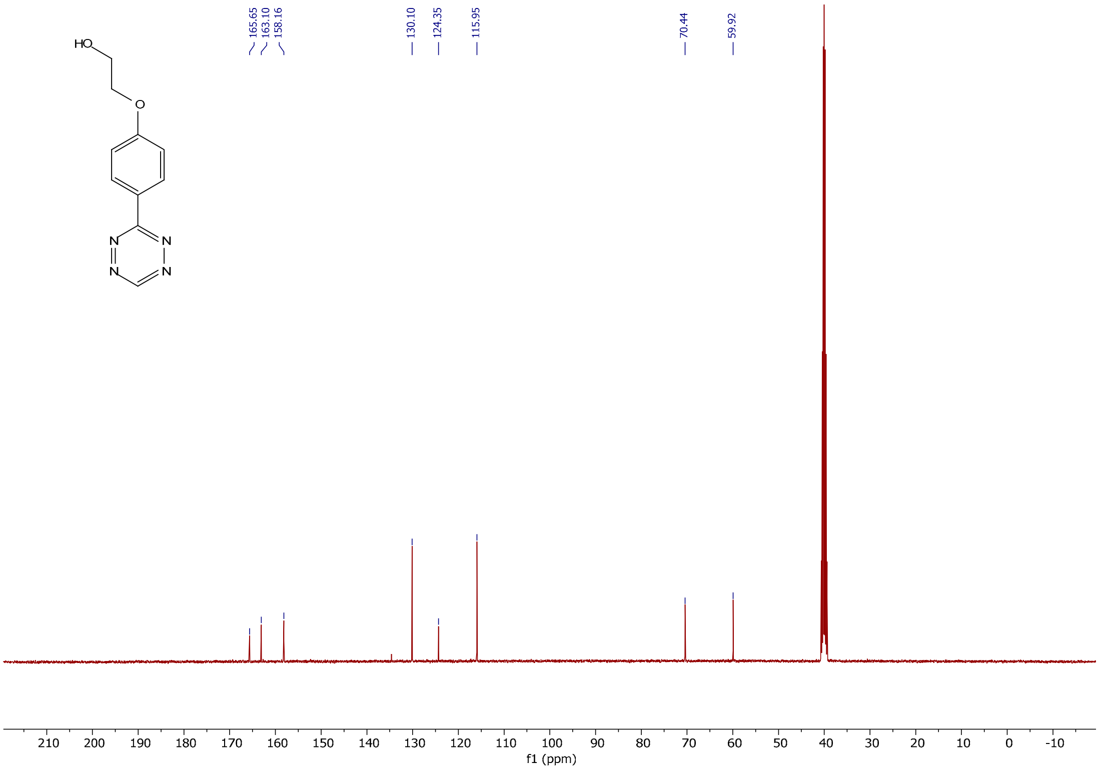

*4-(((2-Fluoroethyl)amino)methyl)benzonitrile (**23**)*

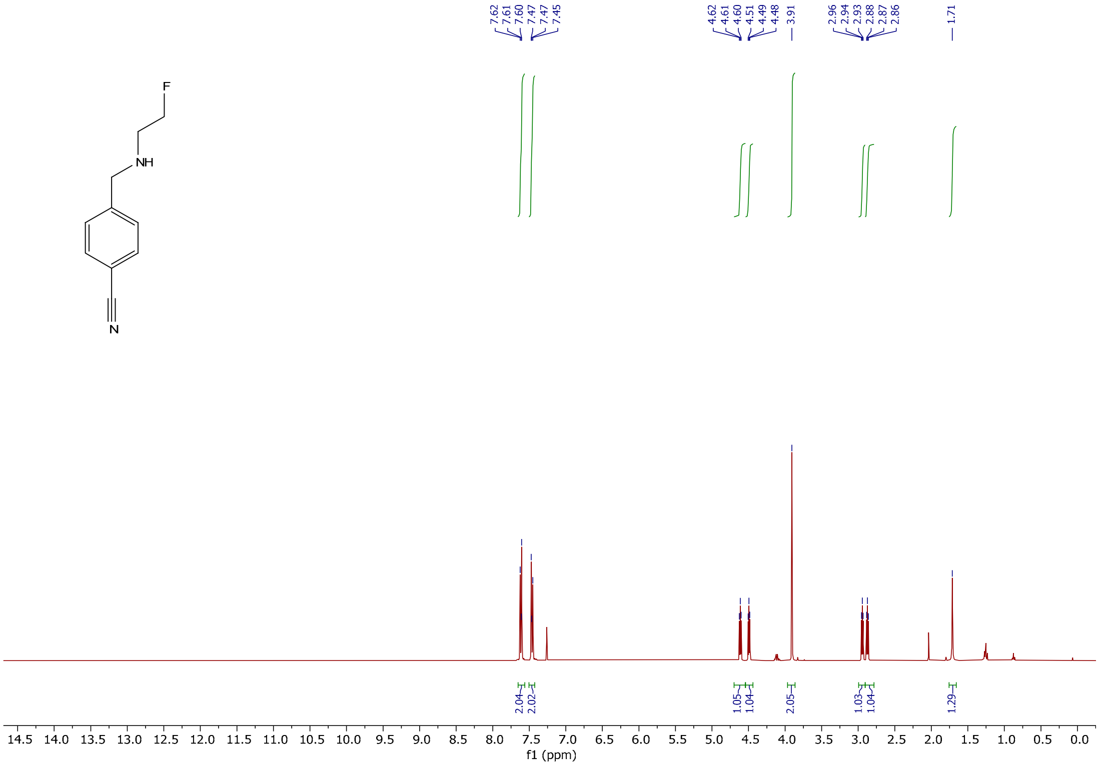

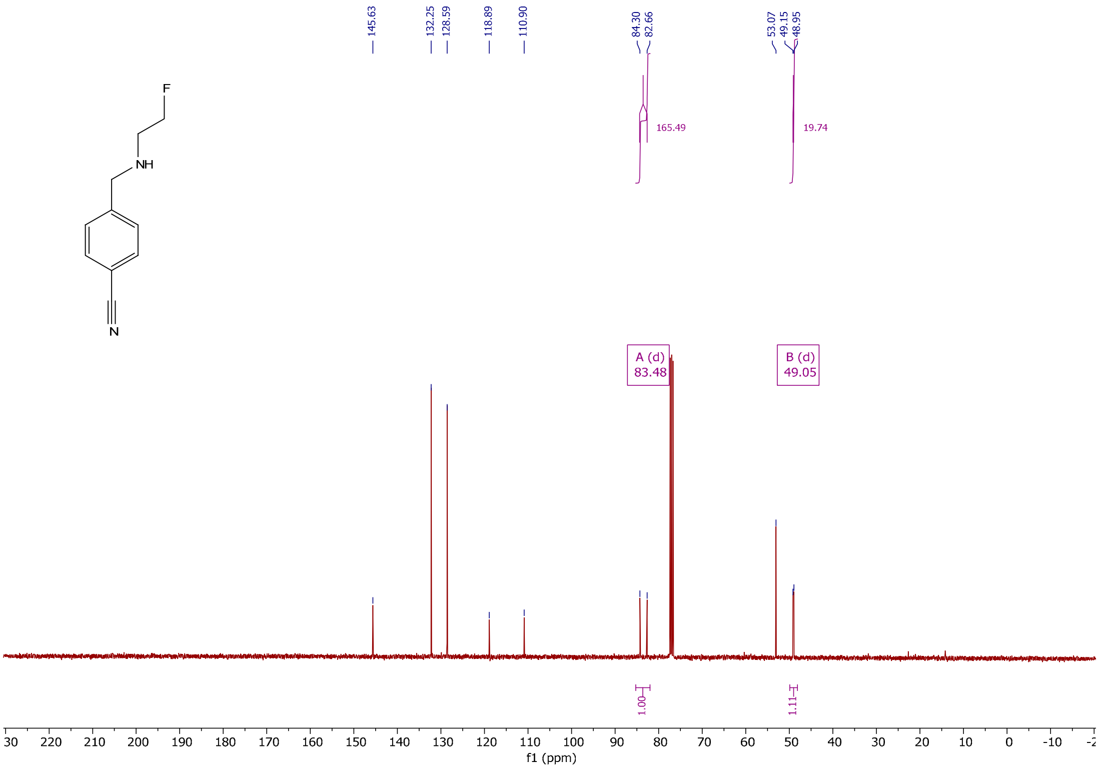

4-(((2-Hydroxyethyl)amino)methyl)benzonitrile ***(24)***

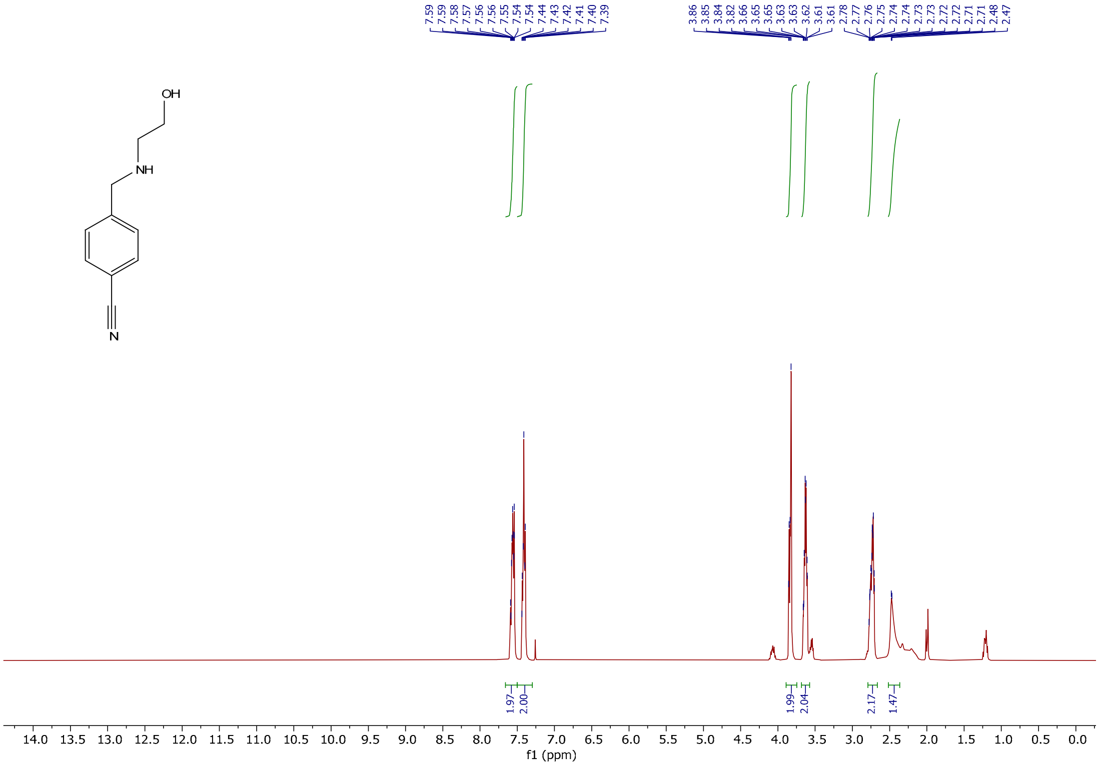

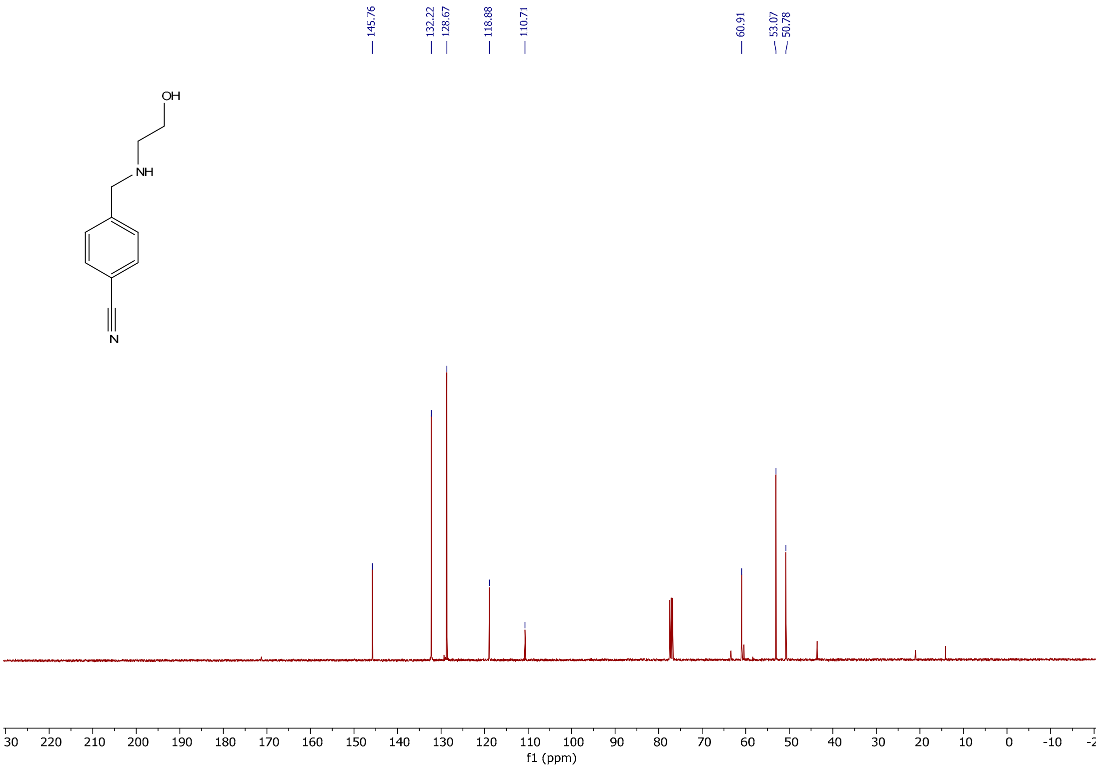

*Tert-butyl 4-cyanobenzyl(2-fluoroethyl)carbamate (**25**)*

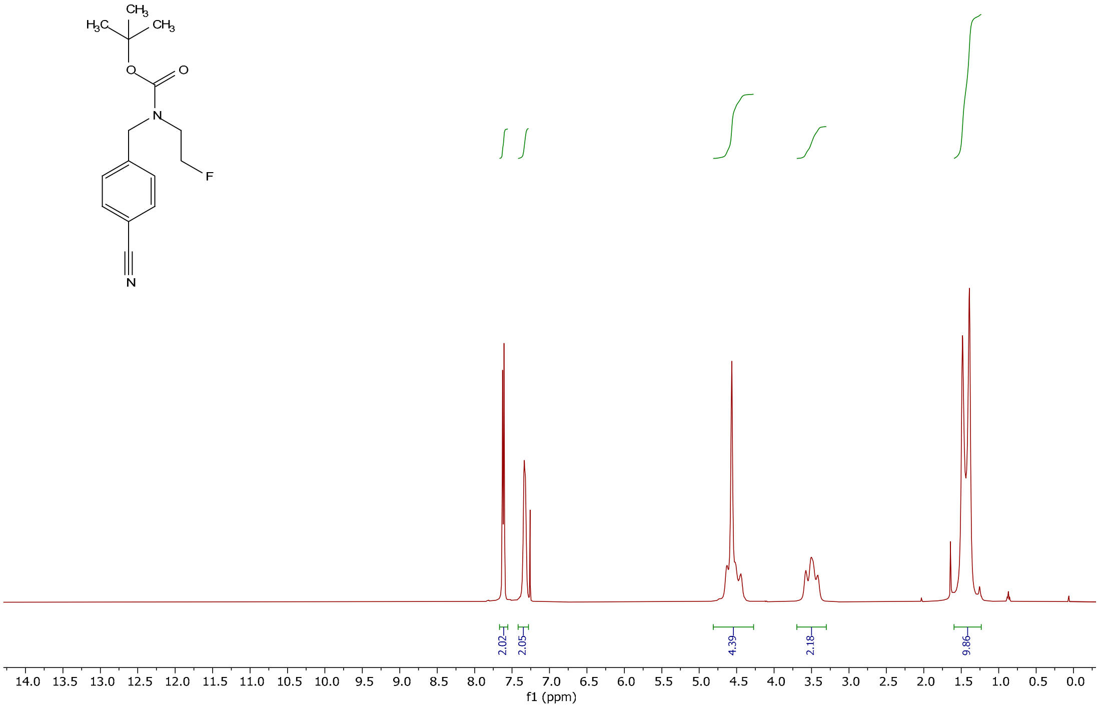

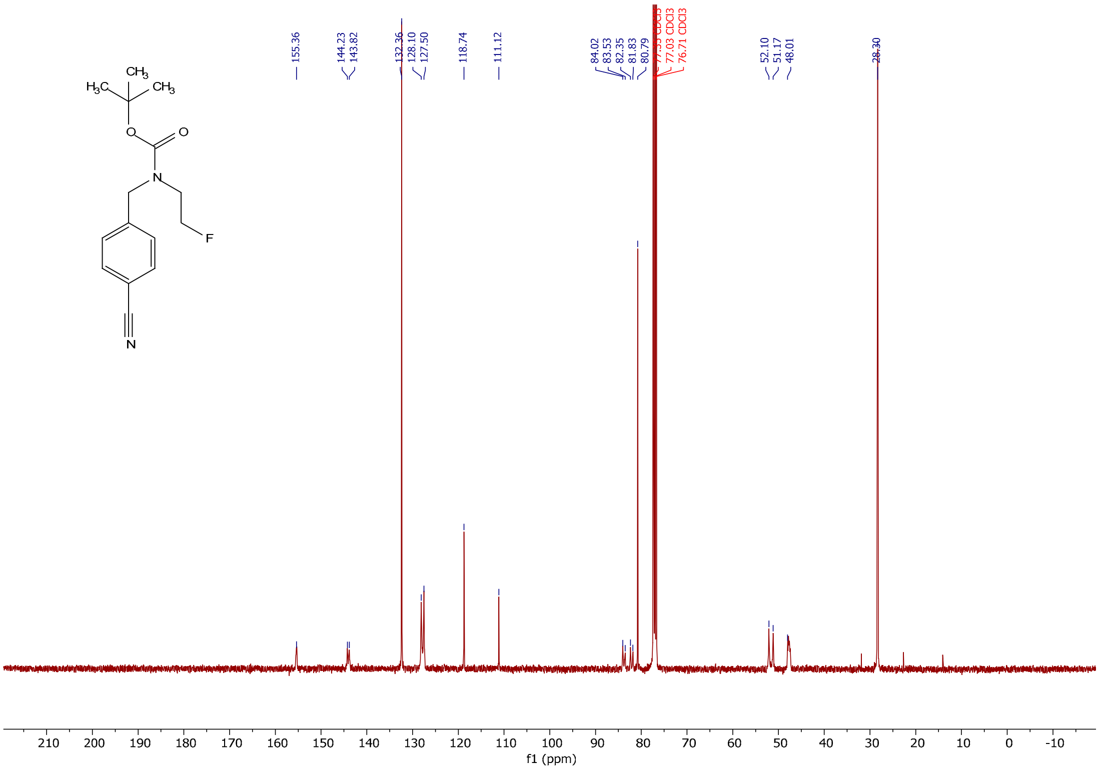

*Tert-butyl (4-cyanobenzyl)(2-hydroxyethyl)carbamate (**26**)*

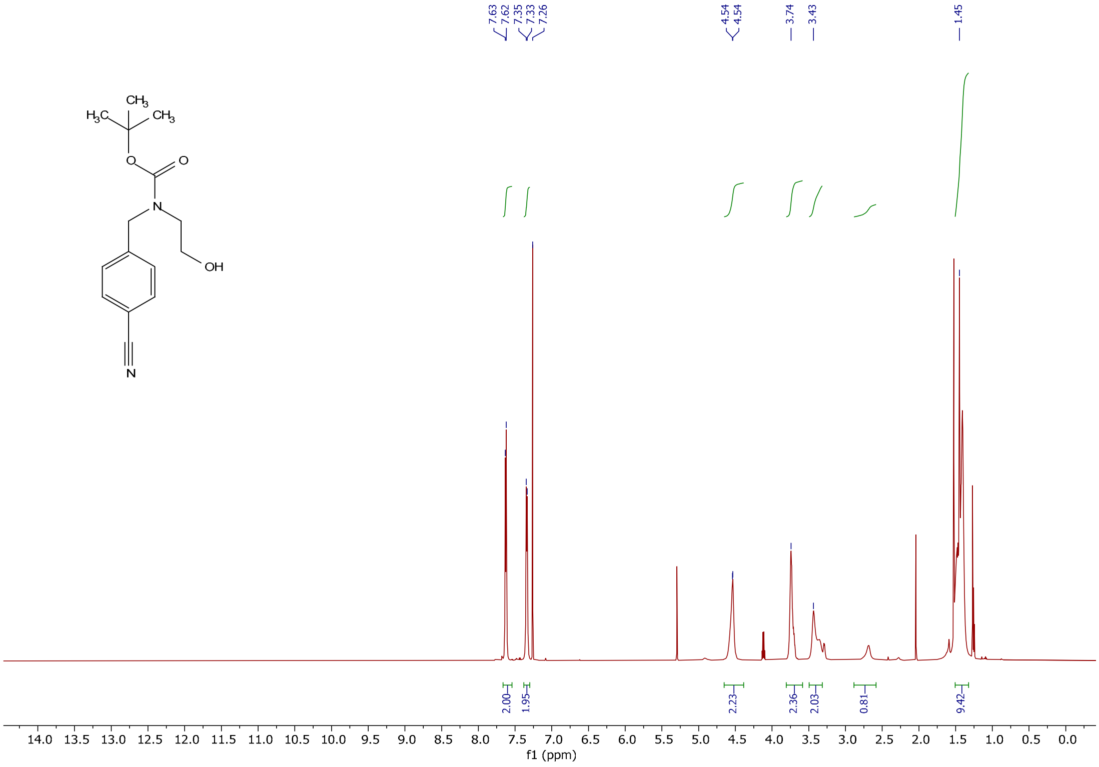

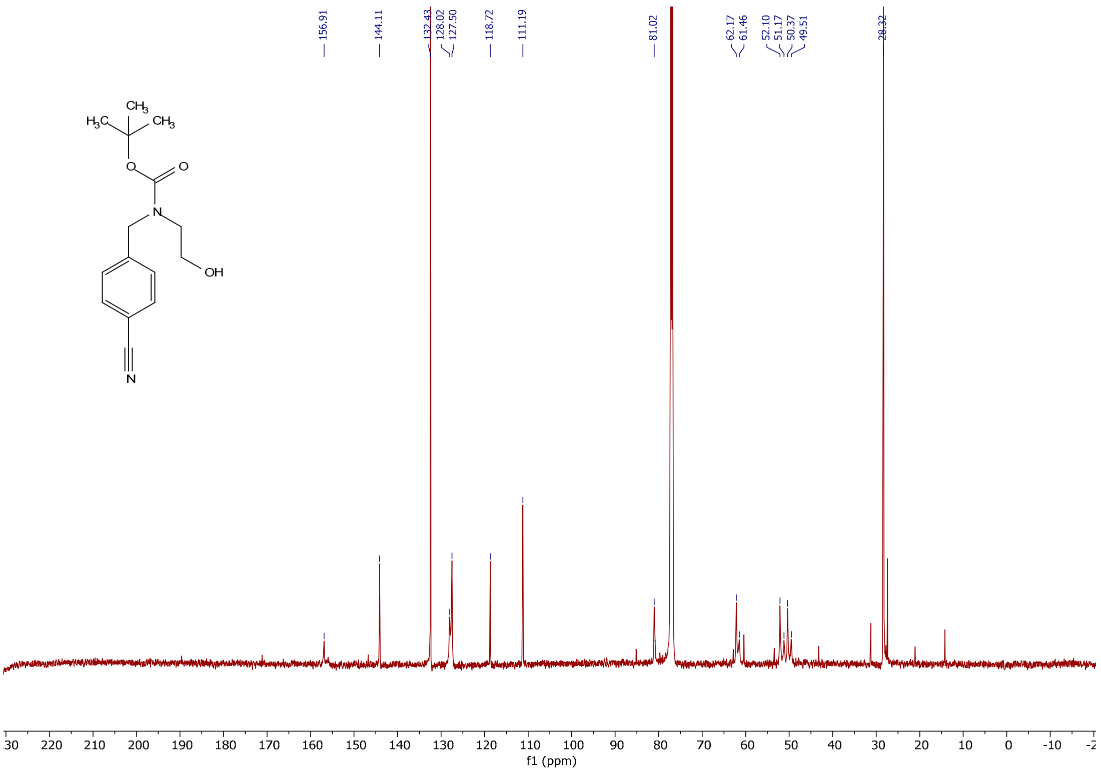

*Tert-butyl 4-(1,2,4,5-tetrazin-3-yl)benzyl(2-fluoroethyl)carbamate (**27**)*

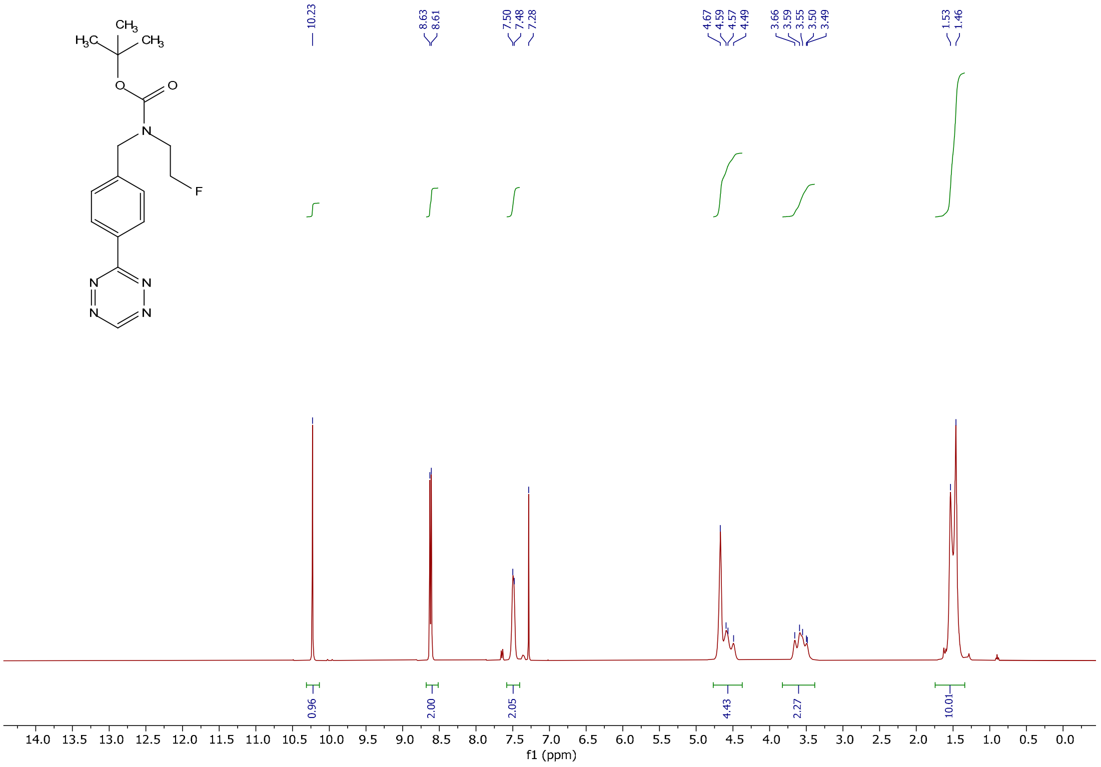

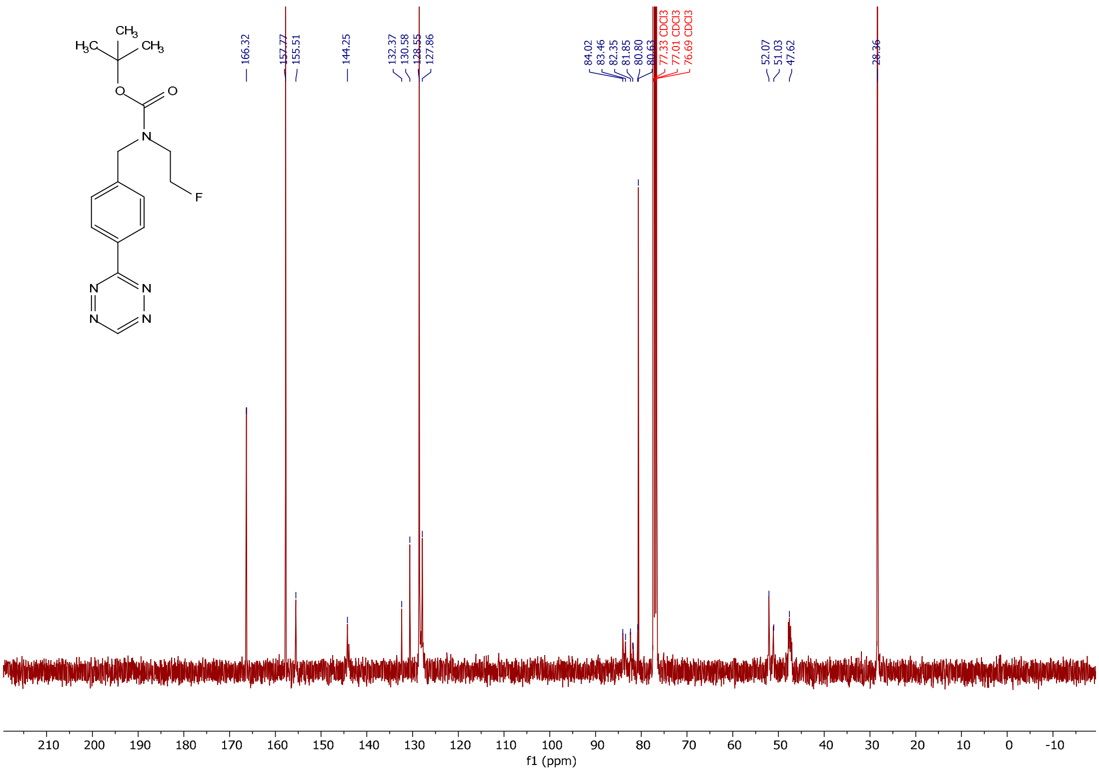

*Tert-butyl (4-(1,2,4,5-tetrazin-3-yl)benzyl)(2-hydroxyethyl)carbamate (**28**)*

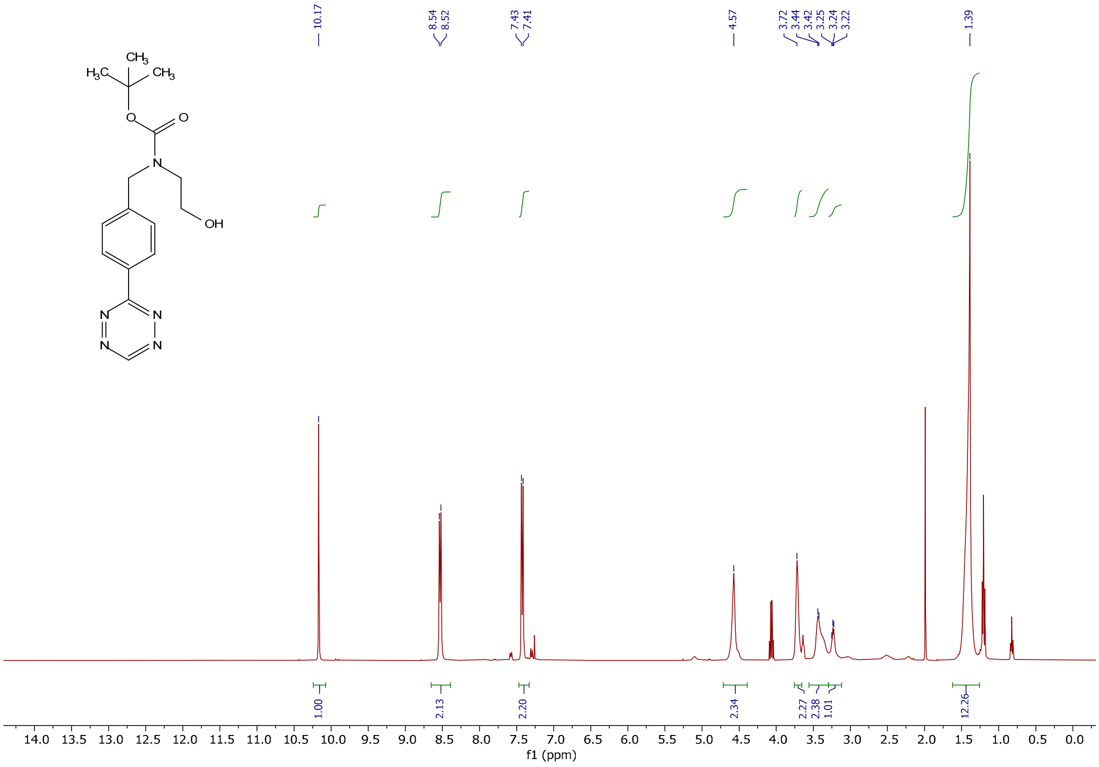

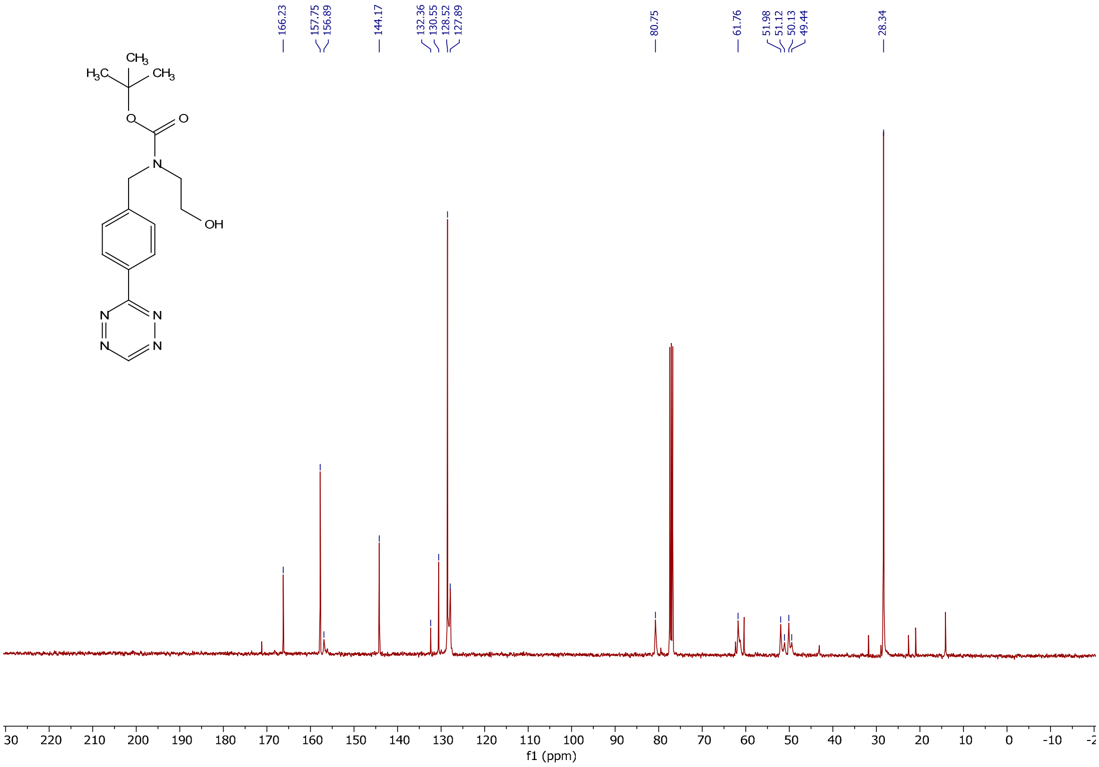

3-(4-(1,2,4,5-Tetrazin-3-yl)benzyl)oxazolidin-2-one

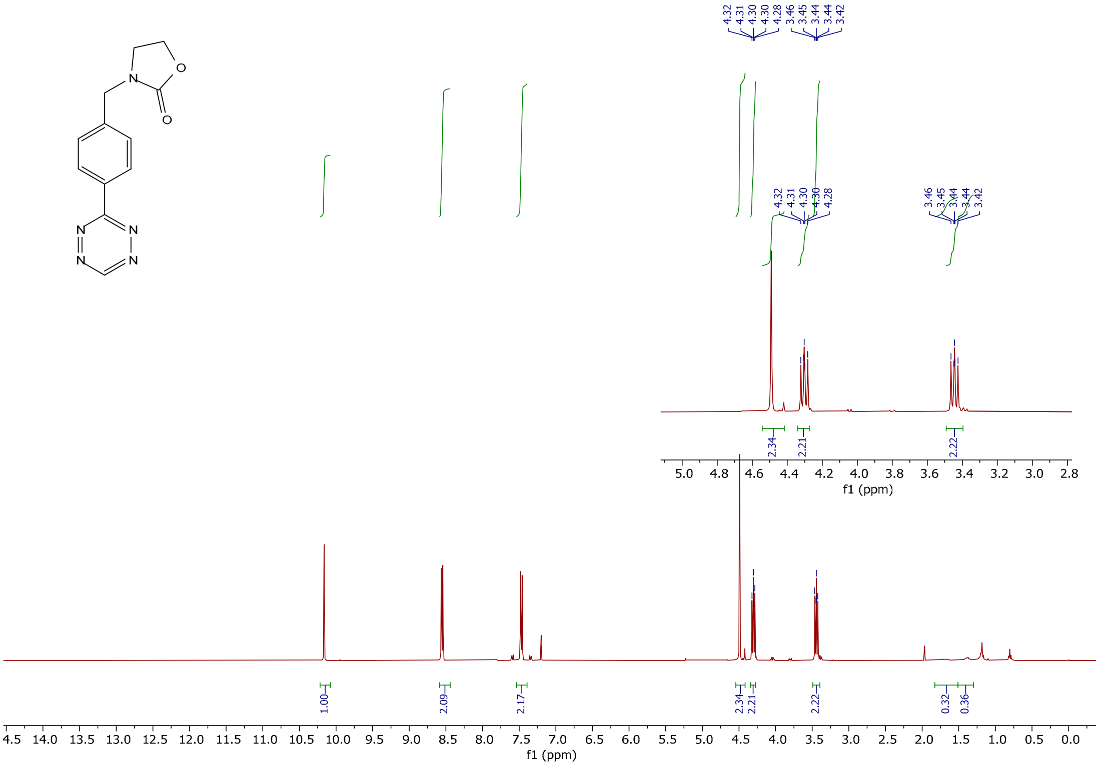

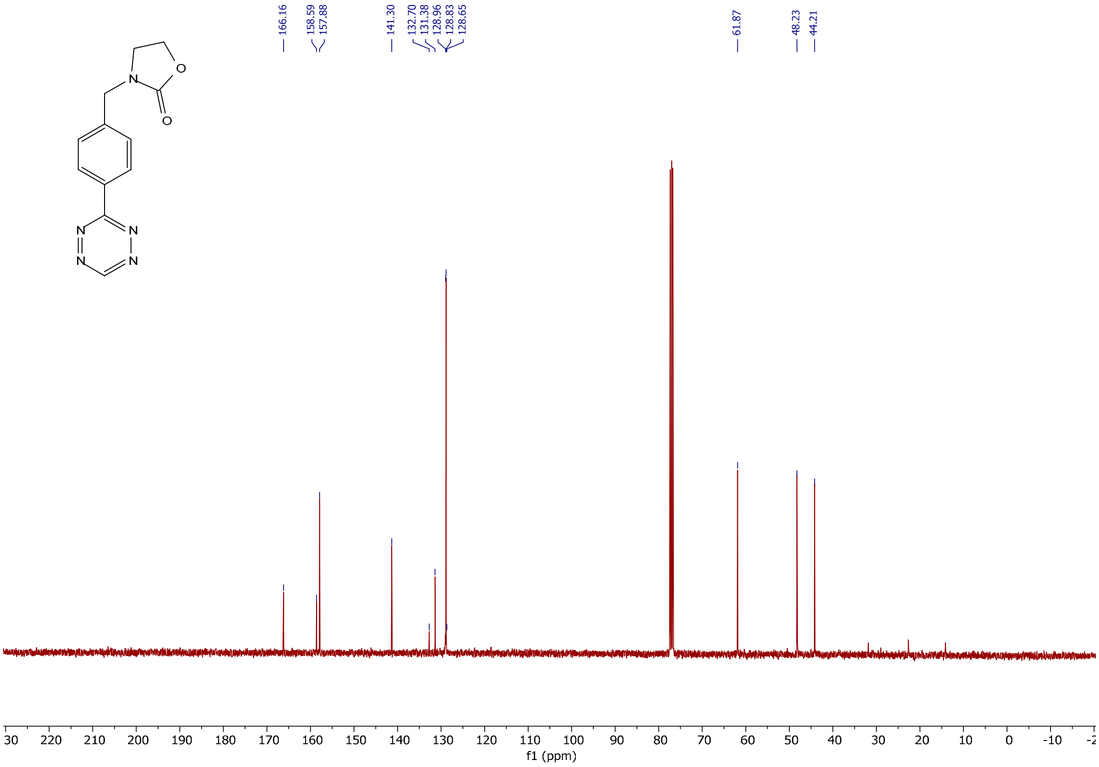

2-((4-(1,2,4,5-Tetrazin-3-yl)benzyl)amino)ethan-1-ol (**29**)

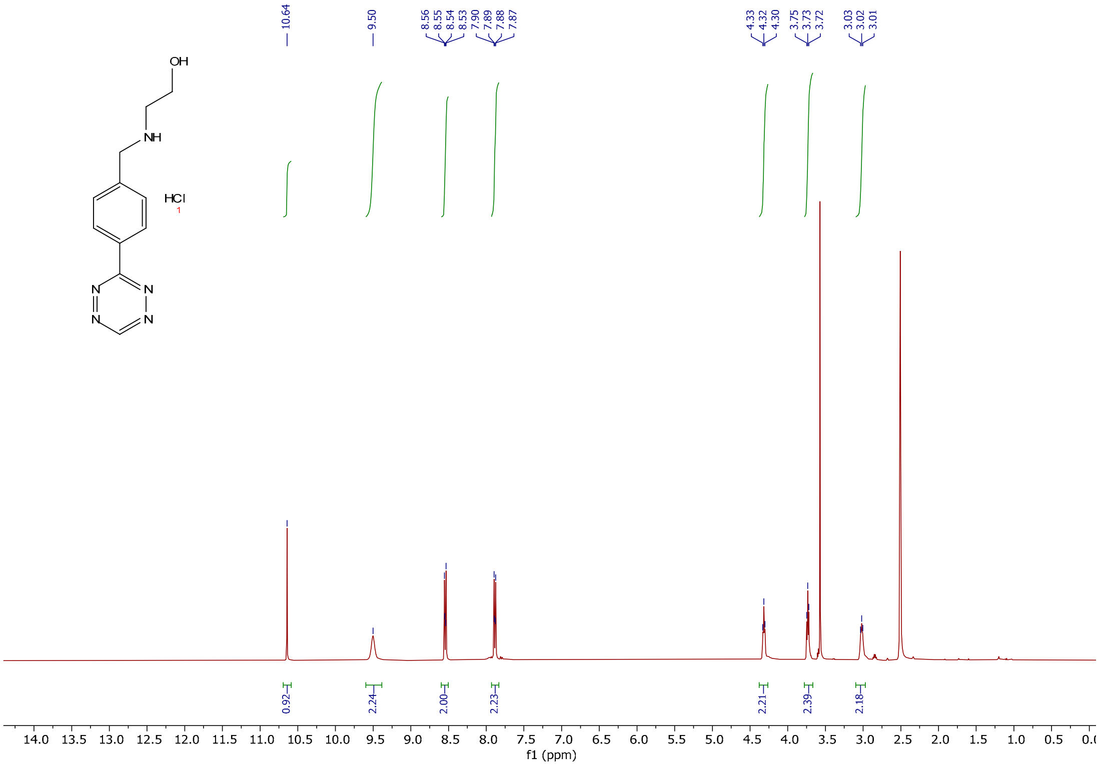

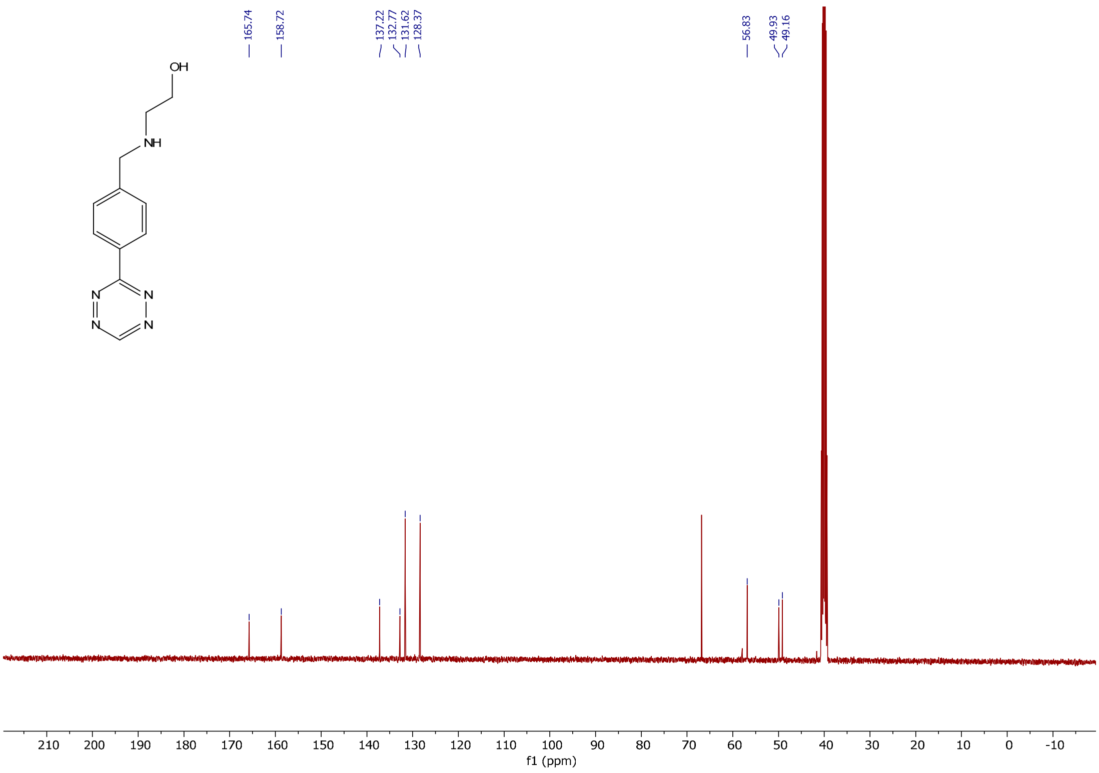

*N-(4-(1,2,4,5-tetrazin-3-yl)benzyl)-2-fluoro-N-tritylethan-1-amine (**30**)*

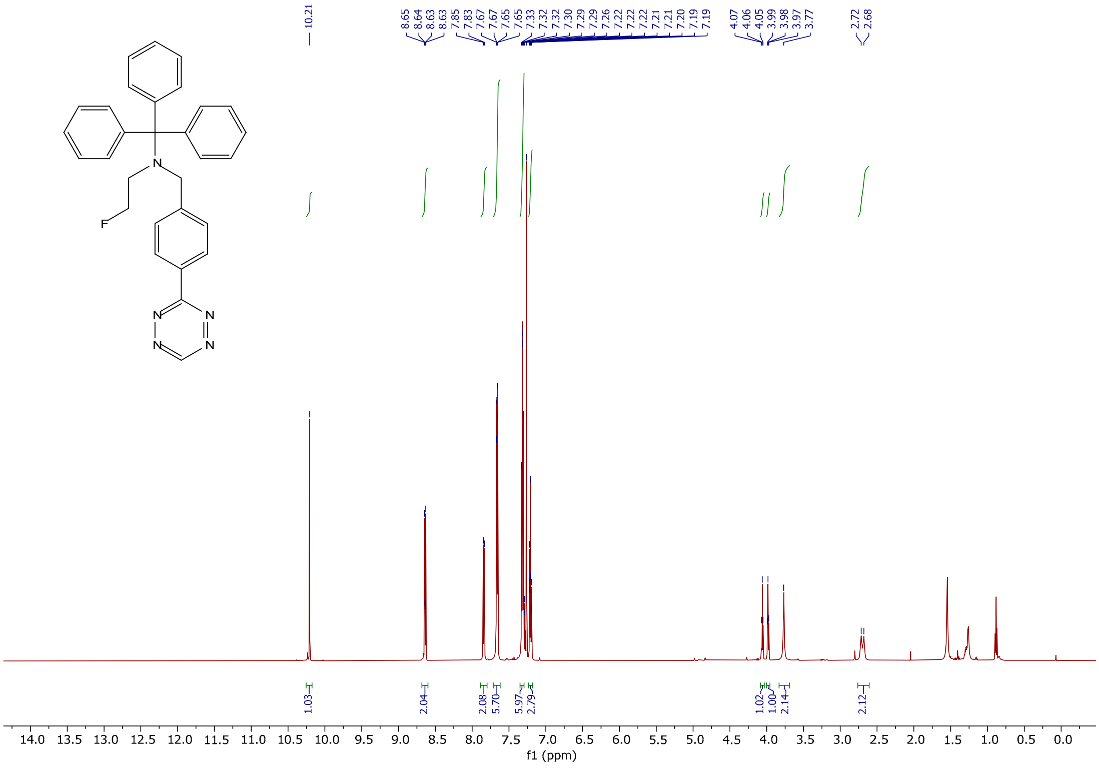

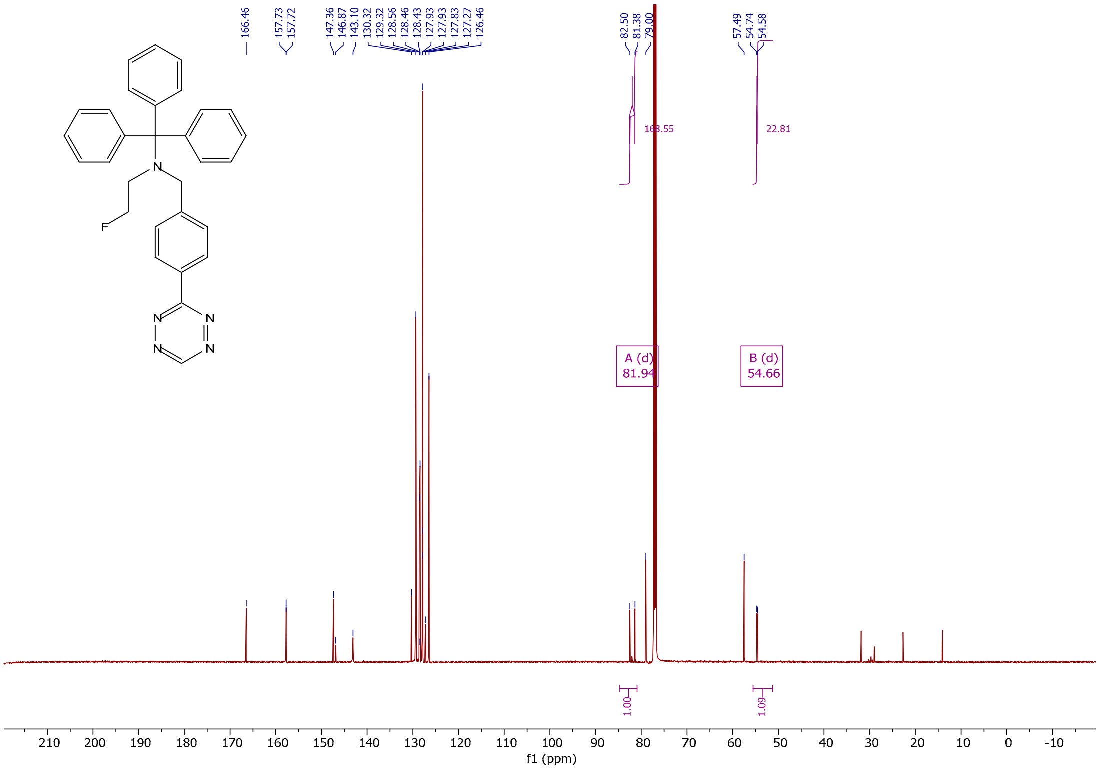

*2-((4-(1,2,4,5-Tetrazin-3-yl)benzyl)(trityl)amino)ethan-1-ol (**31**)*

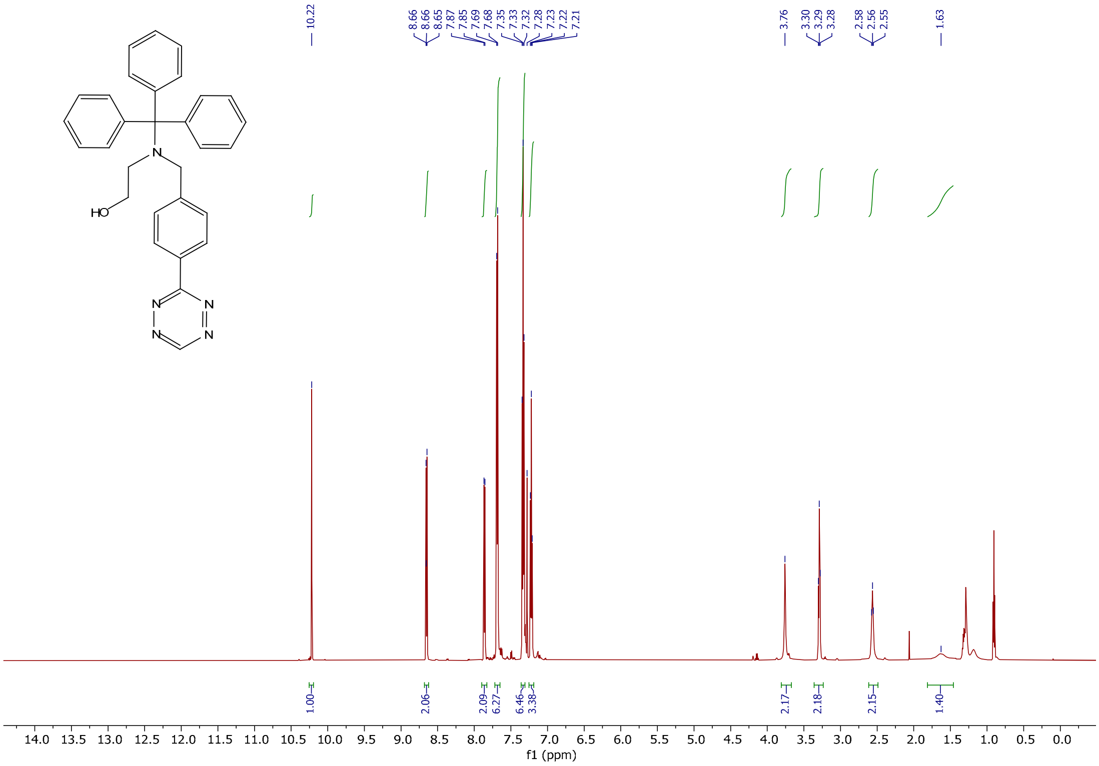

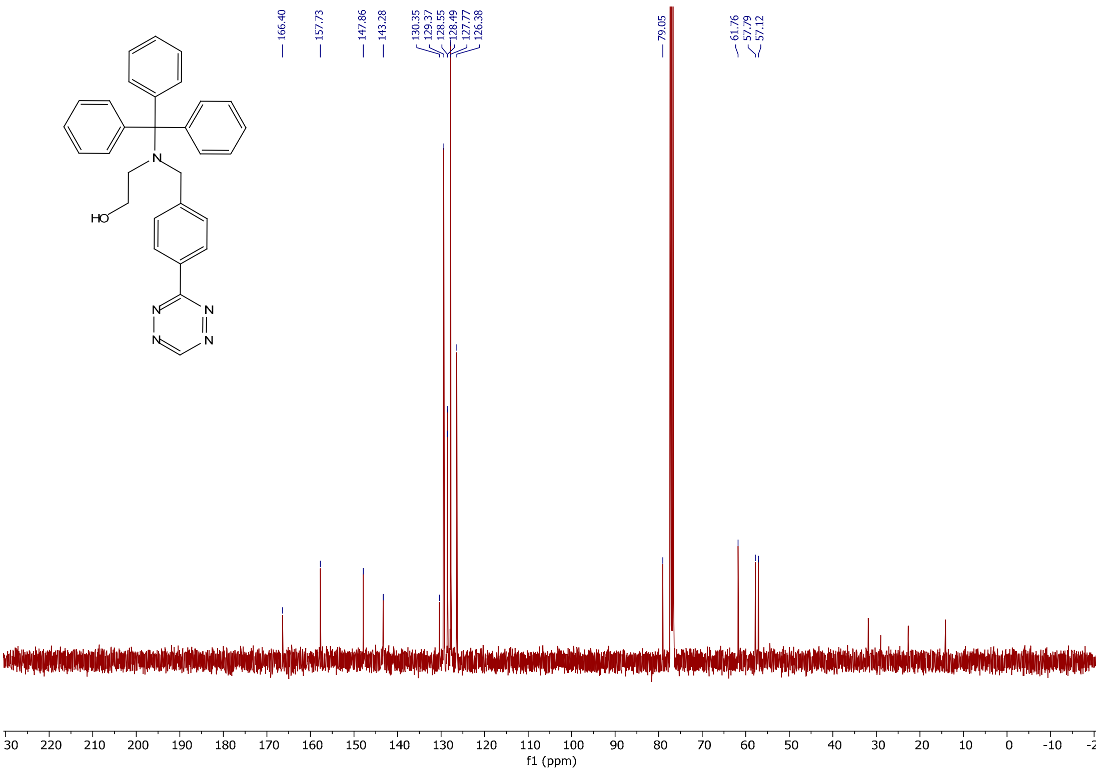

*6-(4-(Pyridin-2-yl)phenyl)nicotinic acid (**32**)*

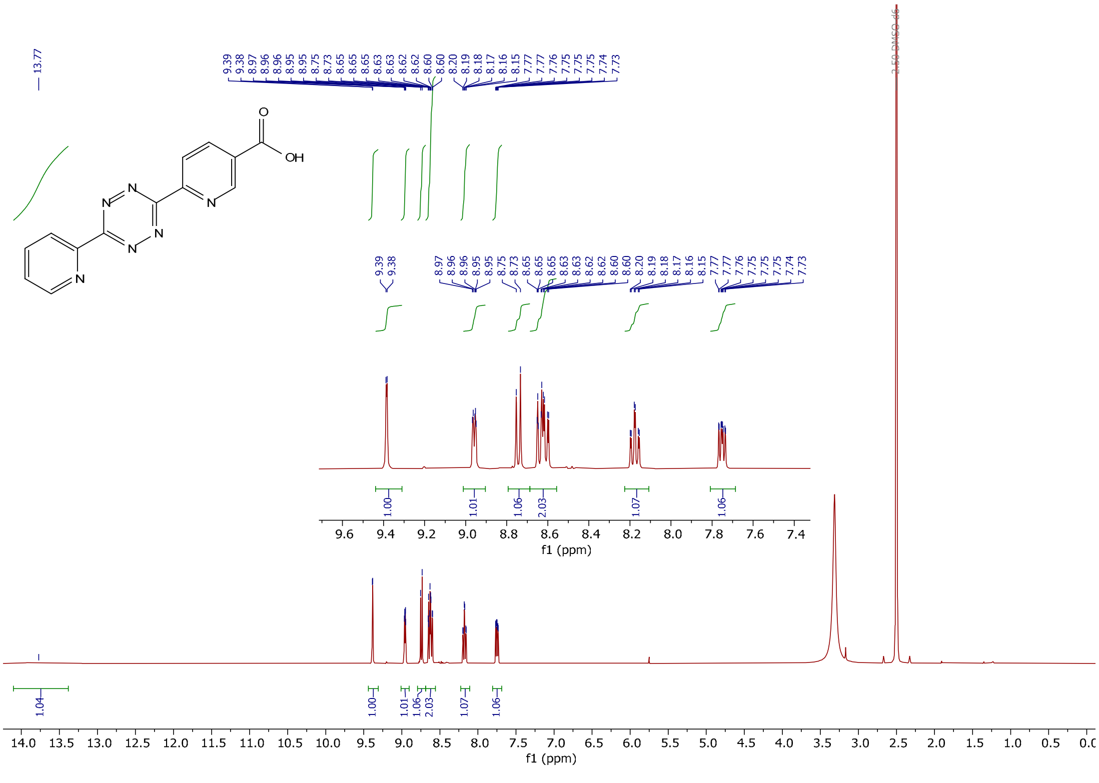

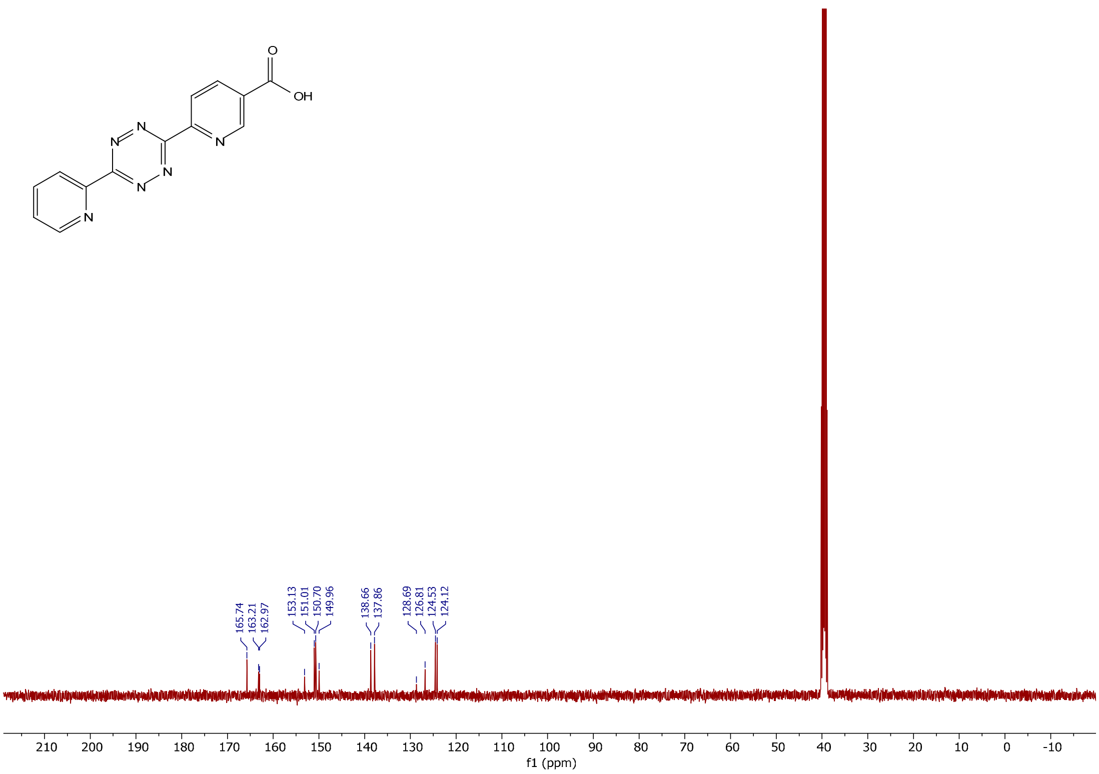

*2-Fluoroethyl 6-(6-(pyridin-2-yl)-1,2,4,5-tetrazin-3-yl)nicotinate (**33**)*

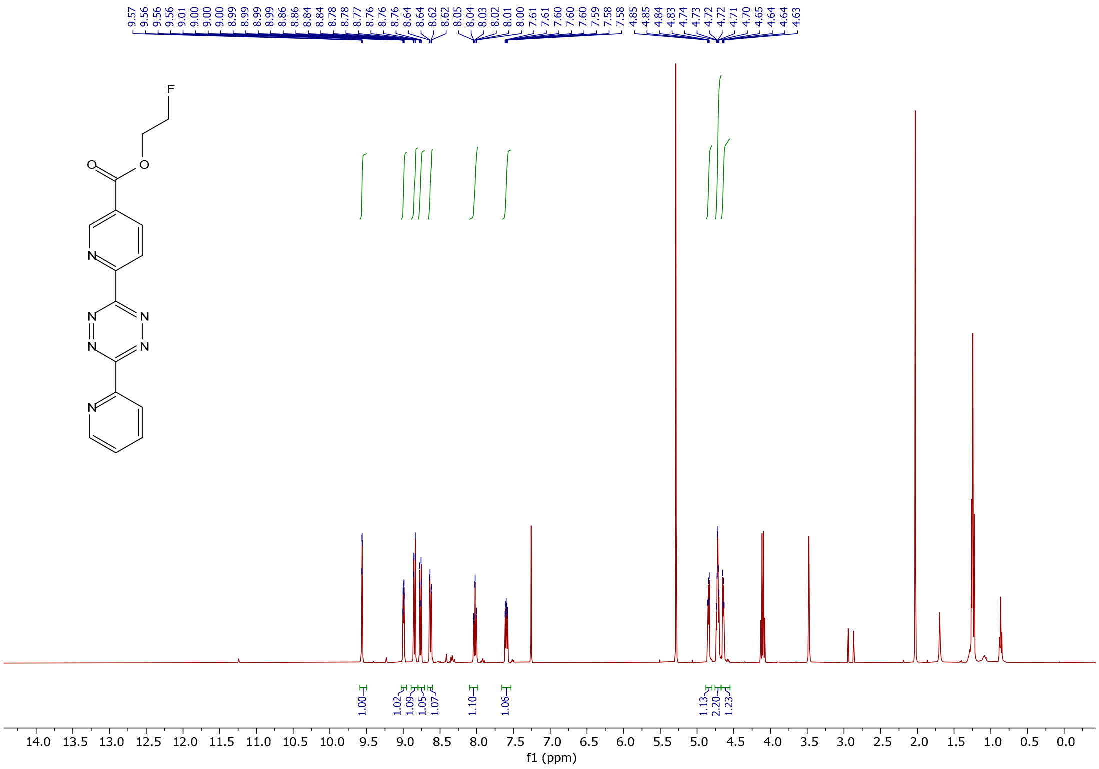

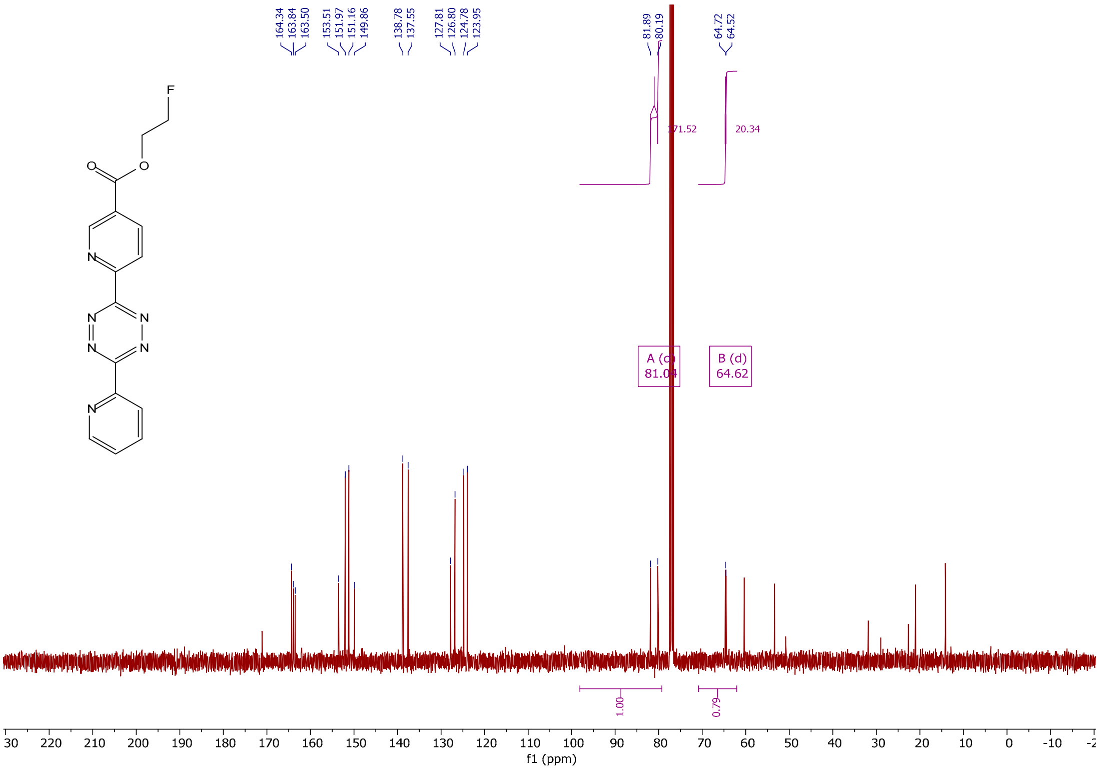

2-(((4-Nitrophenyl)sulfonyl)oxy)ethyl 6-(6-(pyridin-2-yl)-1,2,4,5-tetrazin-3-yl)nicotinate ***(33a)***

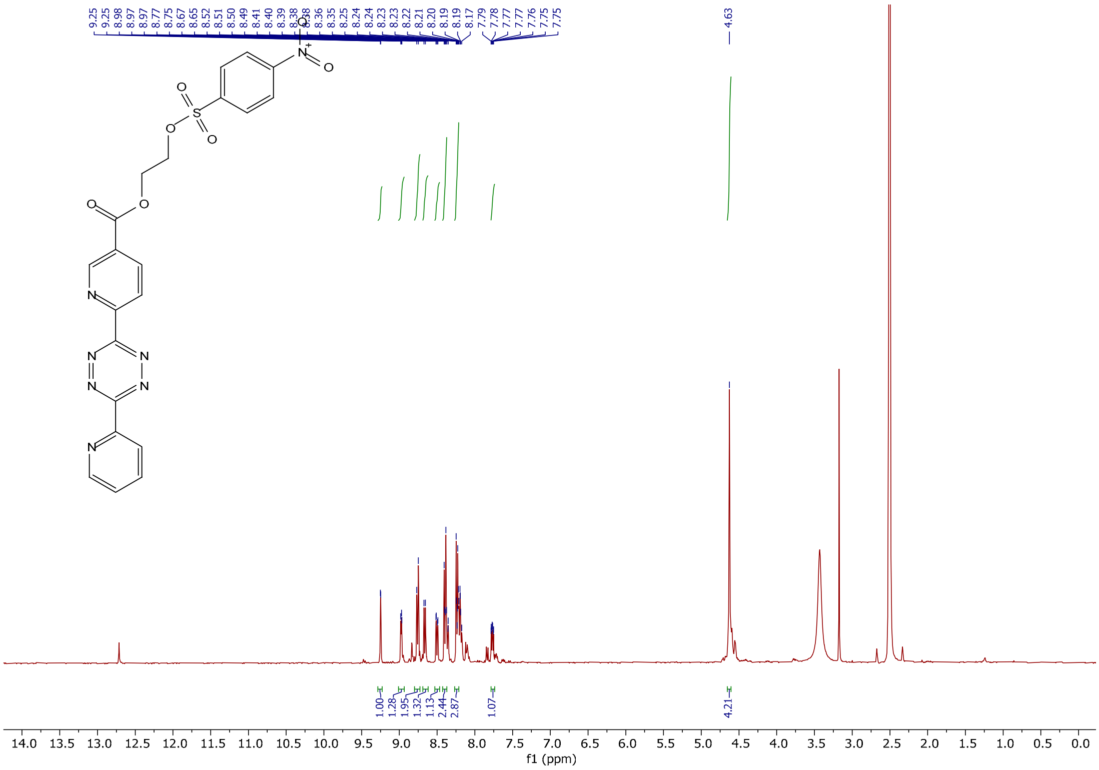

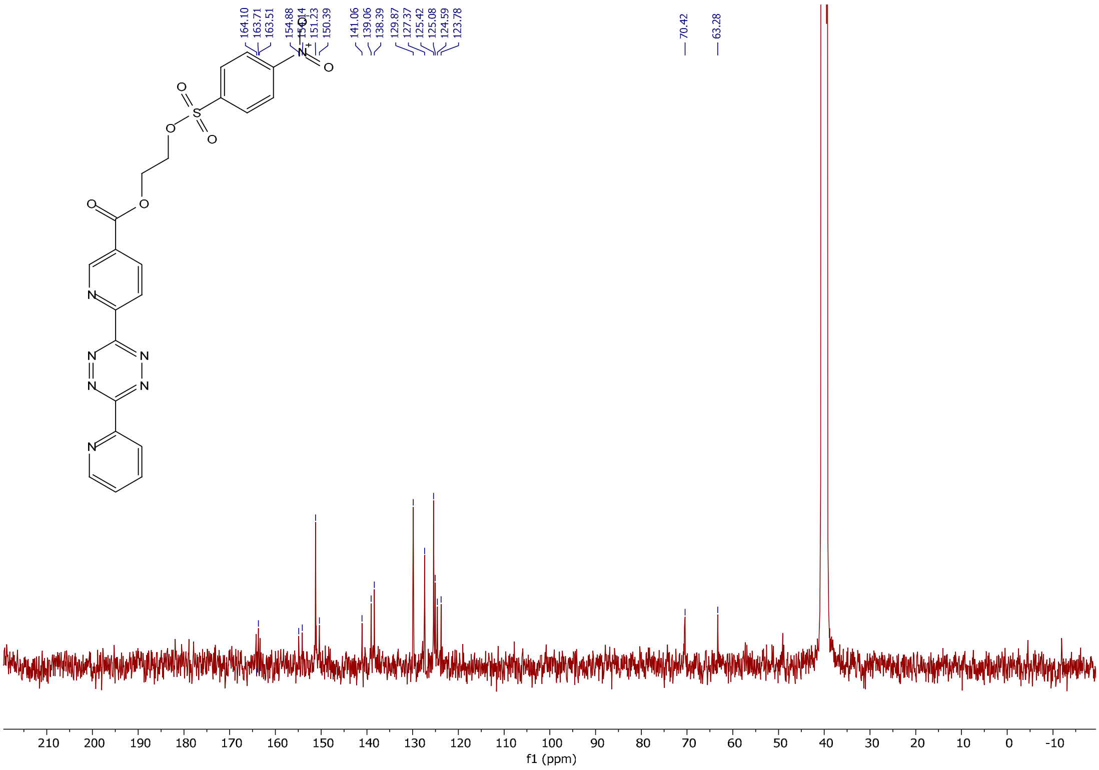

*5-(Bromomethyl)picolinonitrile (**35**)*

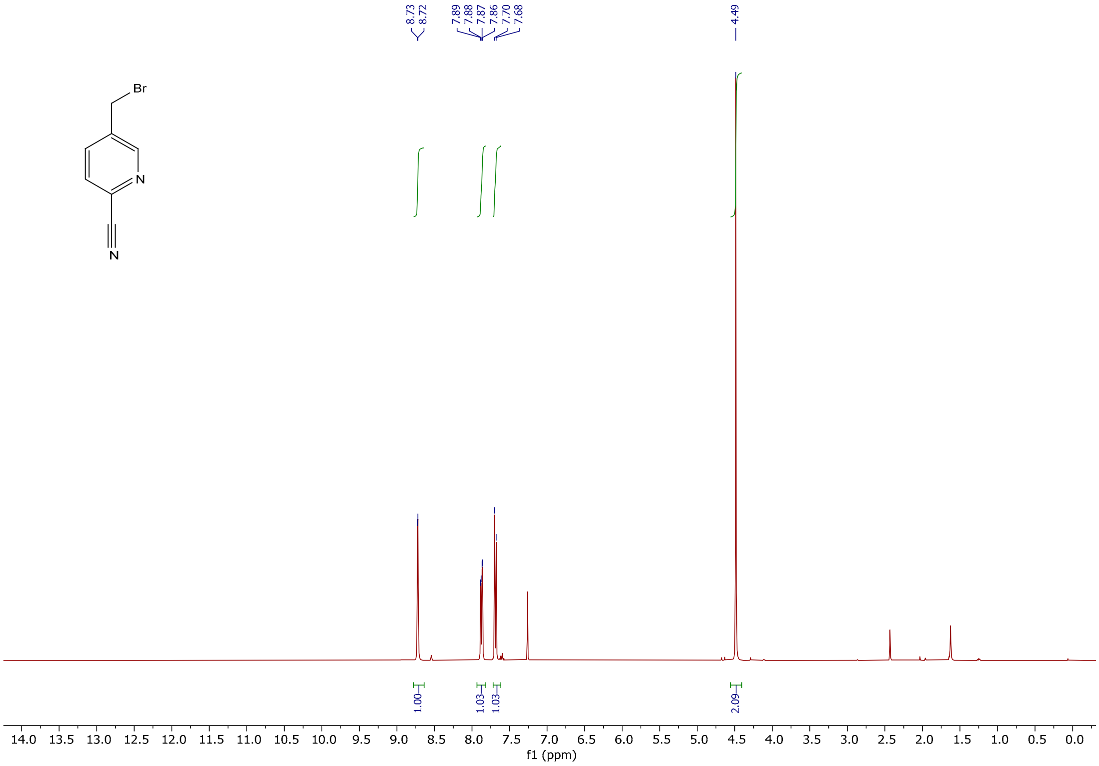

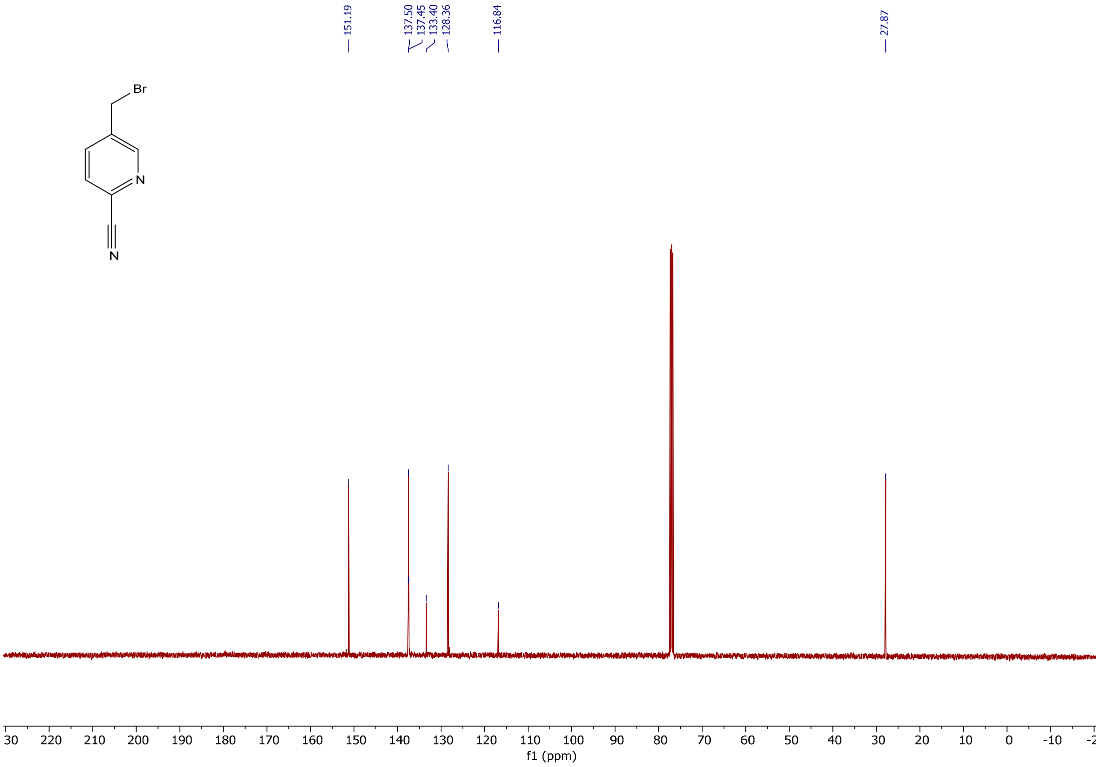

*5-((2-Hydroxyethoxy)methyl)picolinonitrile (**36**)*

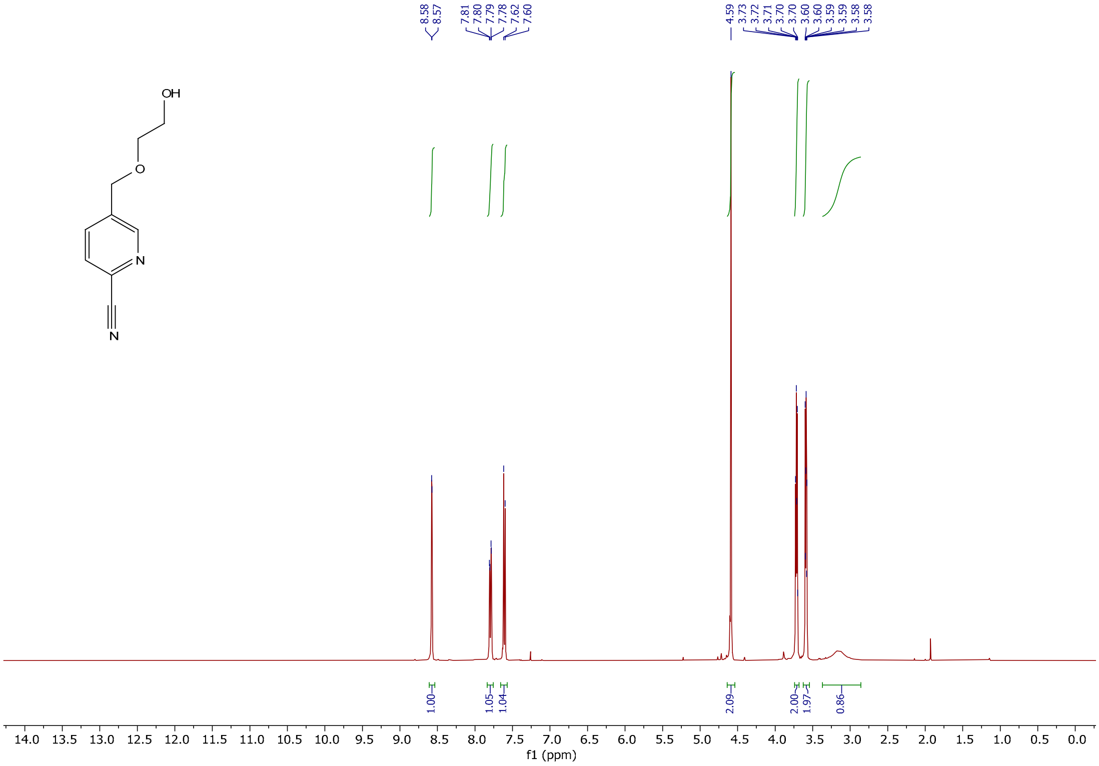

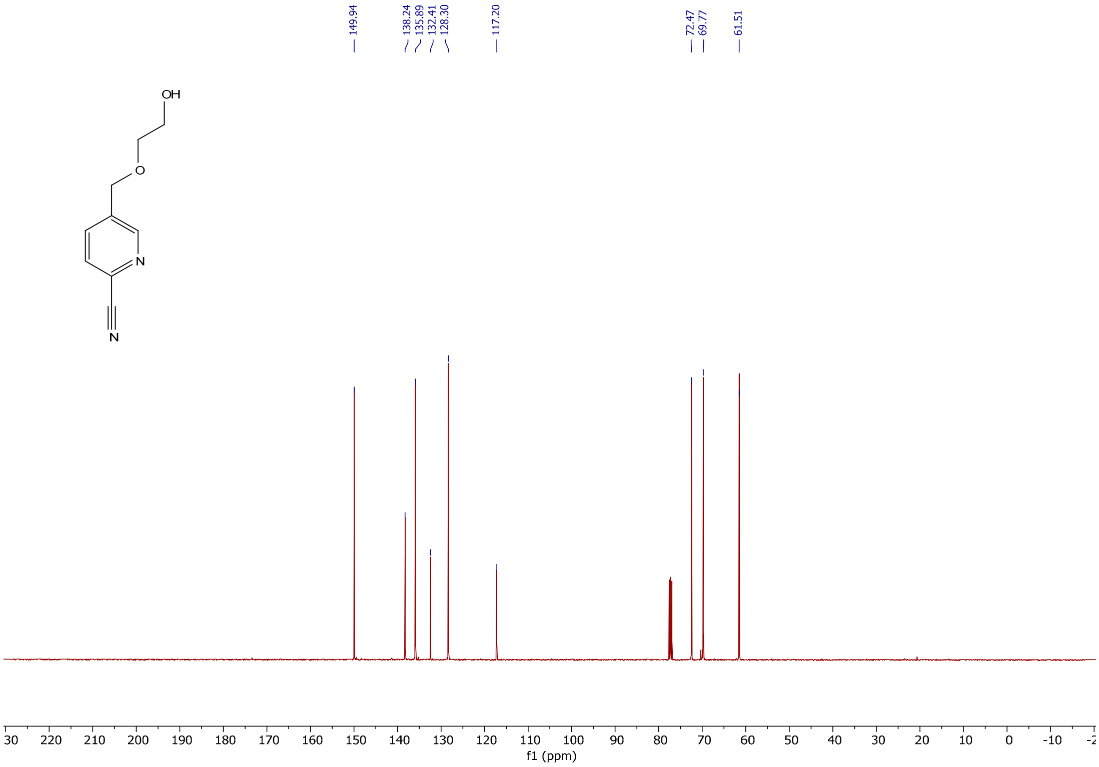

*2-((6-(6-(Pyridin-2-yl)-1,2,4,5-tetrazin-3-yl)pyridin-3-yl)methoxy)ethan-1-ol (**37**)*

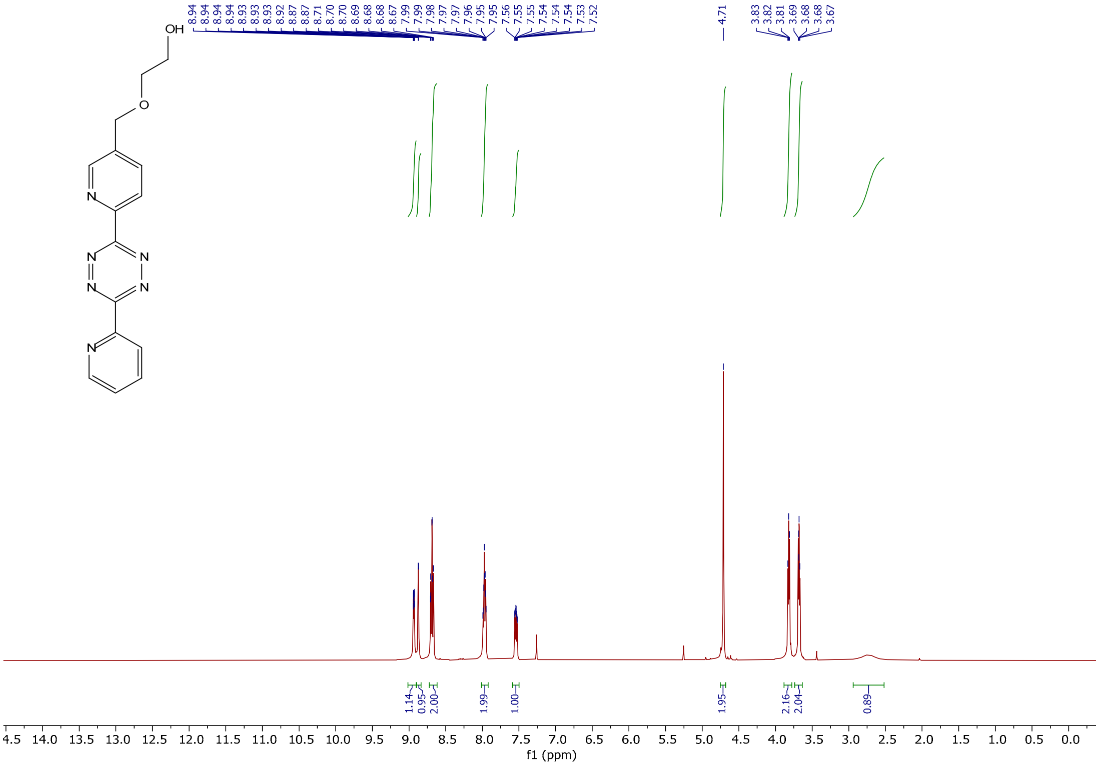

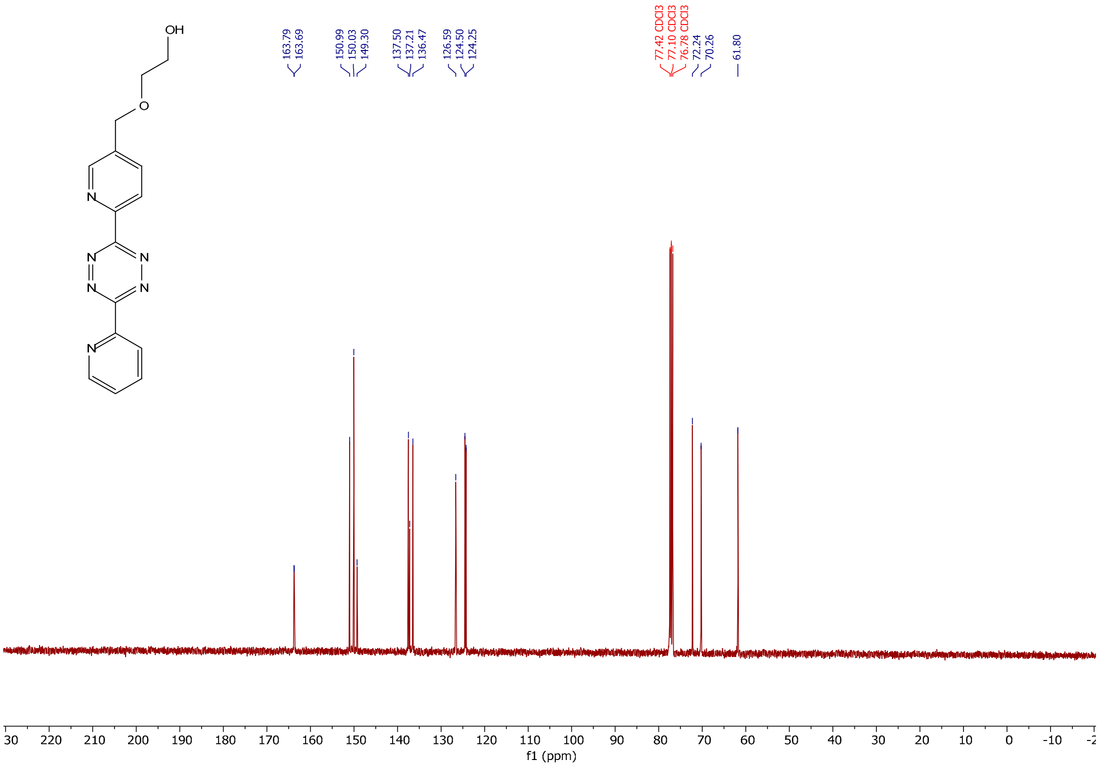

*3-(5-((2-fluoroethoxy)methyl)pyridin-2-yl)-6-(pyridin-2-yl)-1,2,4,5-tetrazine (**38**)*

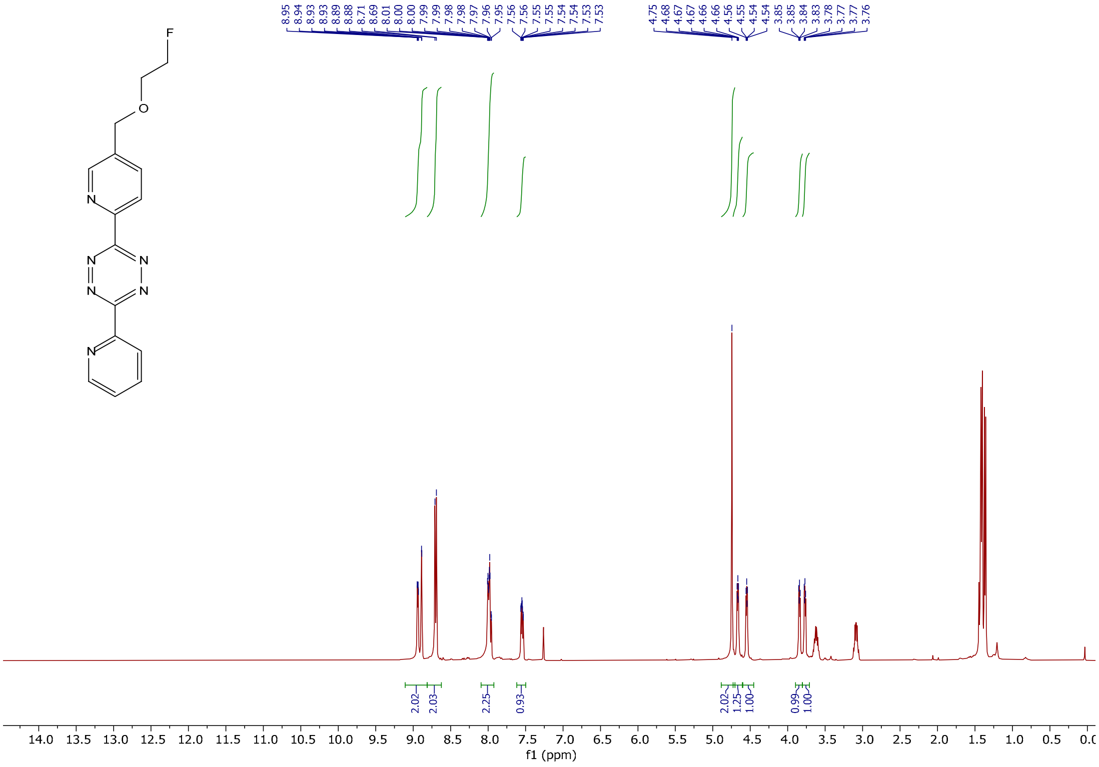

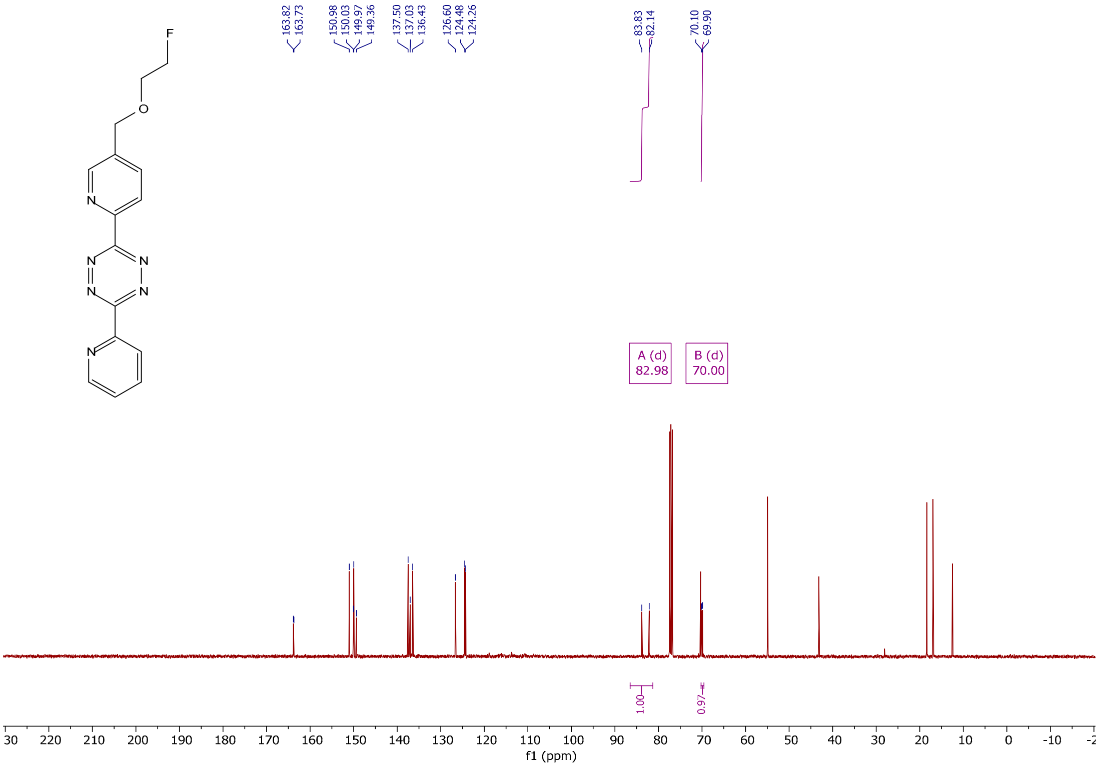

2-((6-(4-(pyridin-2-yl)phenyl)pyridin-3-yl)methoxy)ethyl 4-methylbenzene sulfonate ***(38a)***

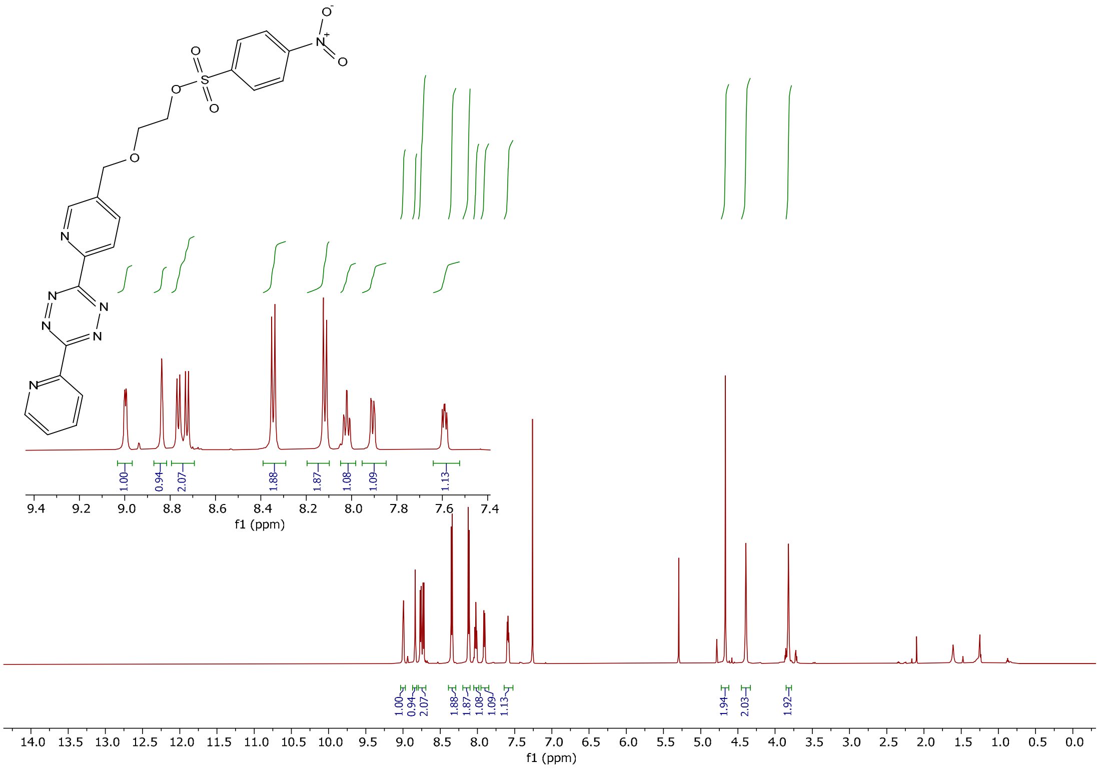

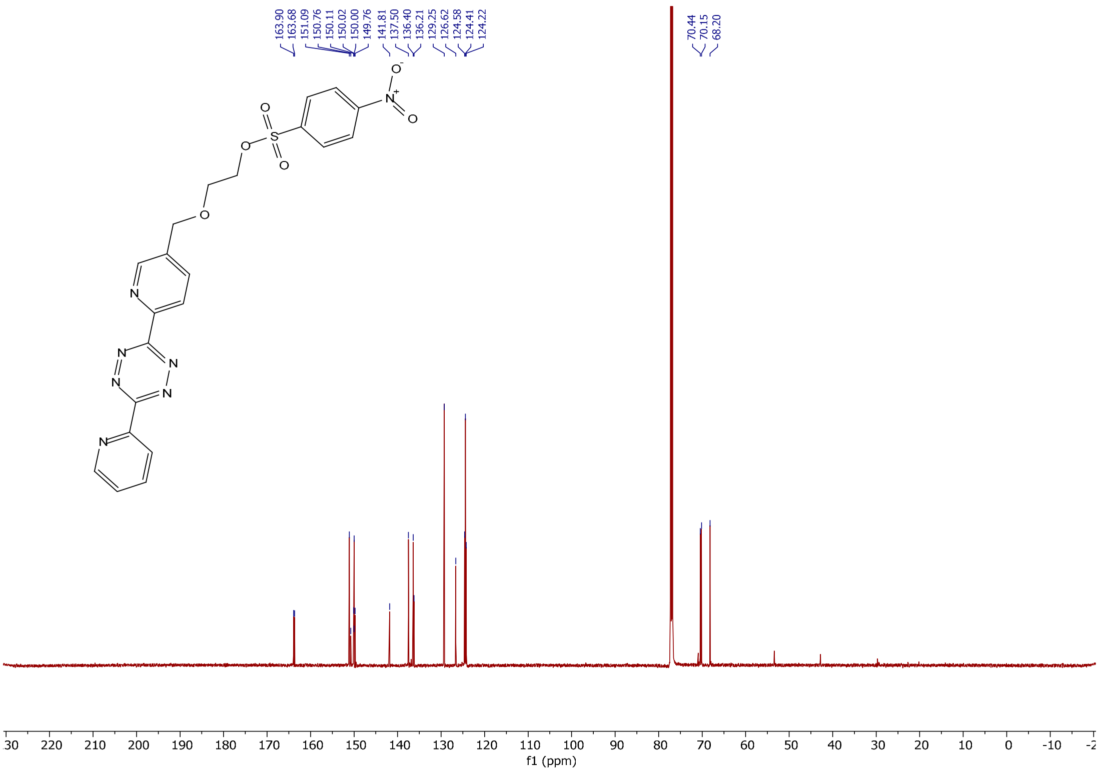

*5-(2-Fluoroethoxy)picolinonitrile (**39**)*

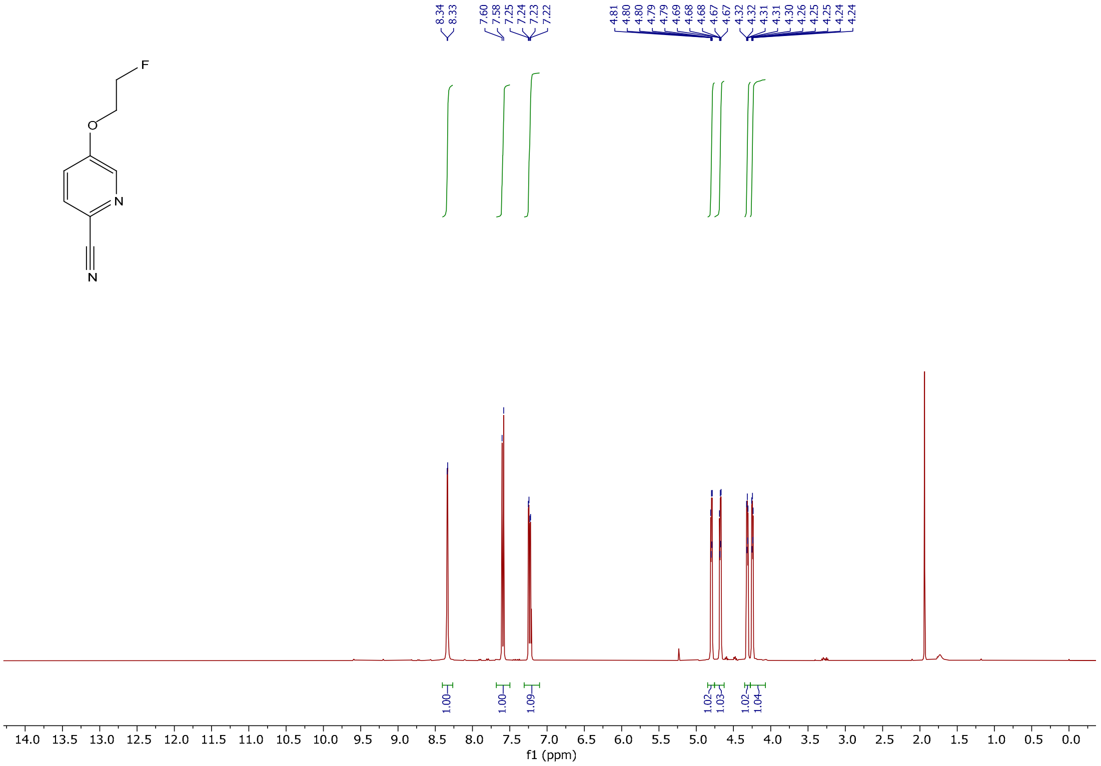

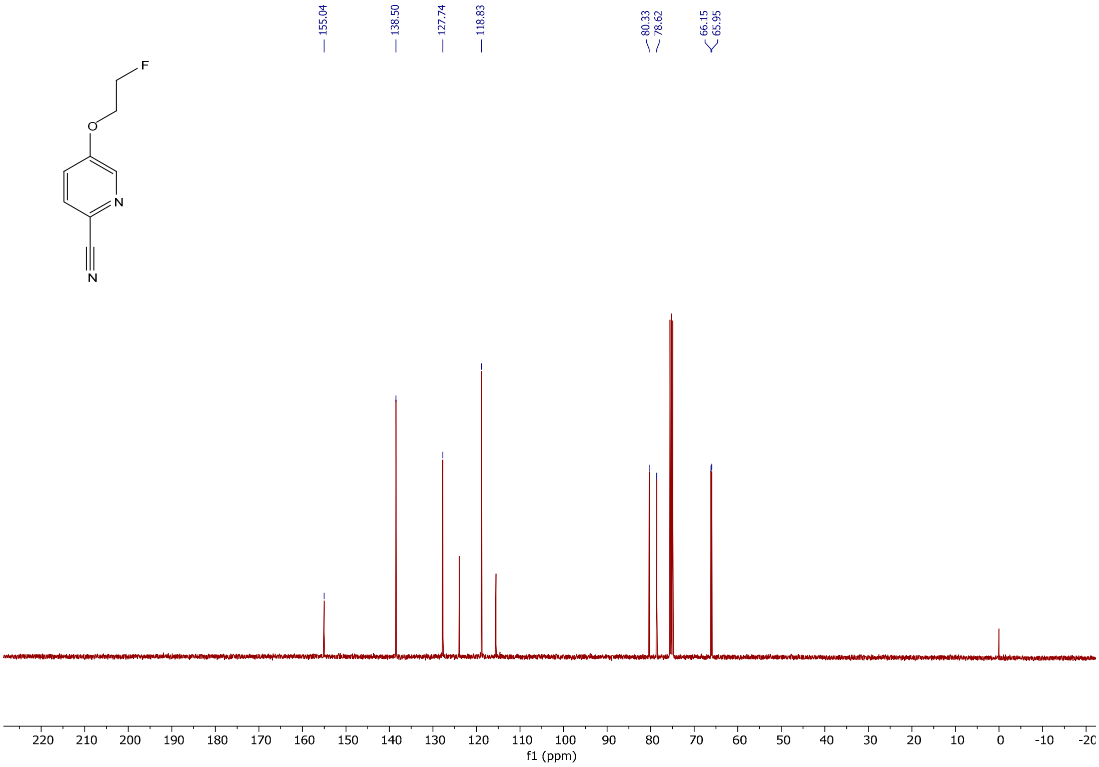

*5-(2-Hydroxyethoxy)picolinonitrile (**40**)*

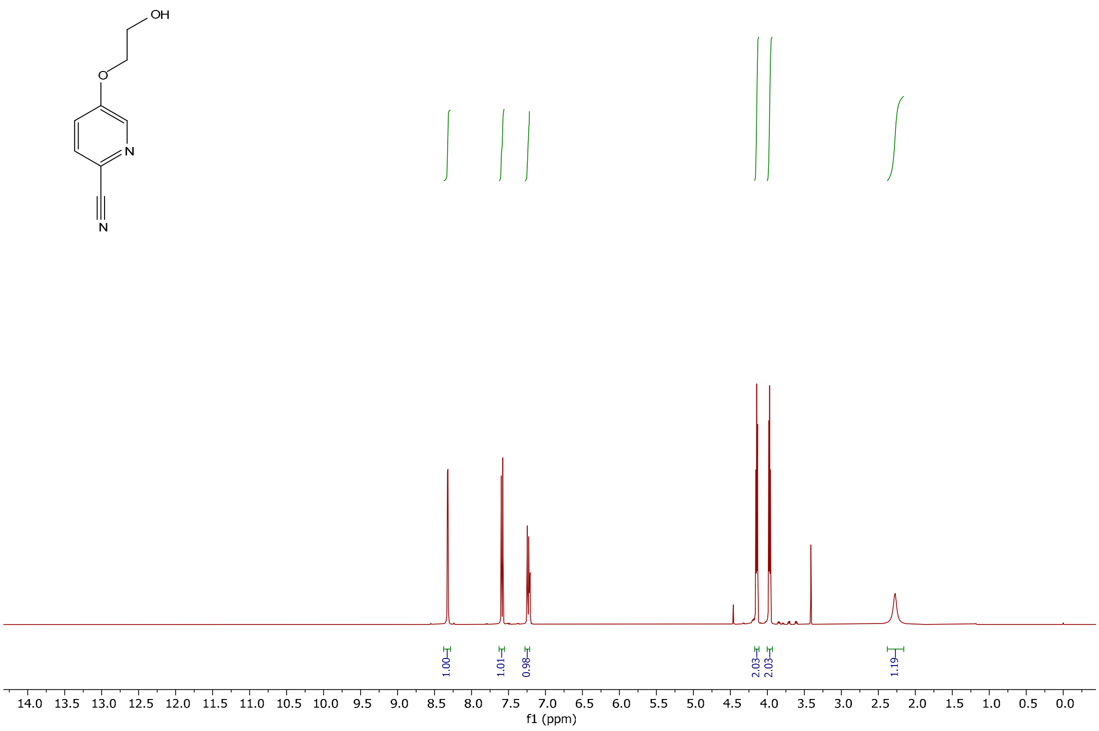

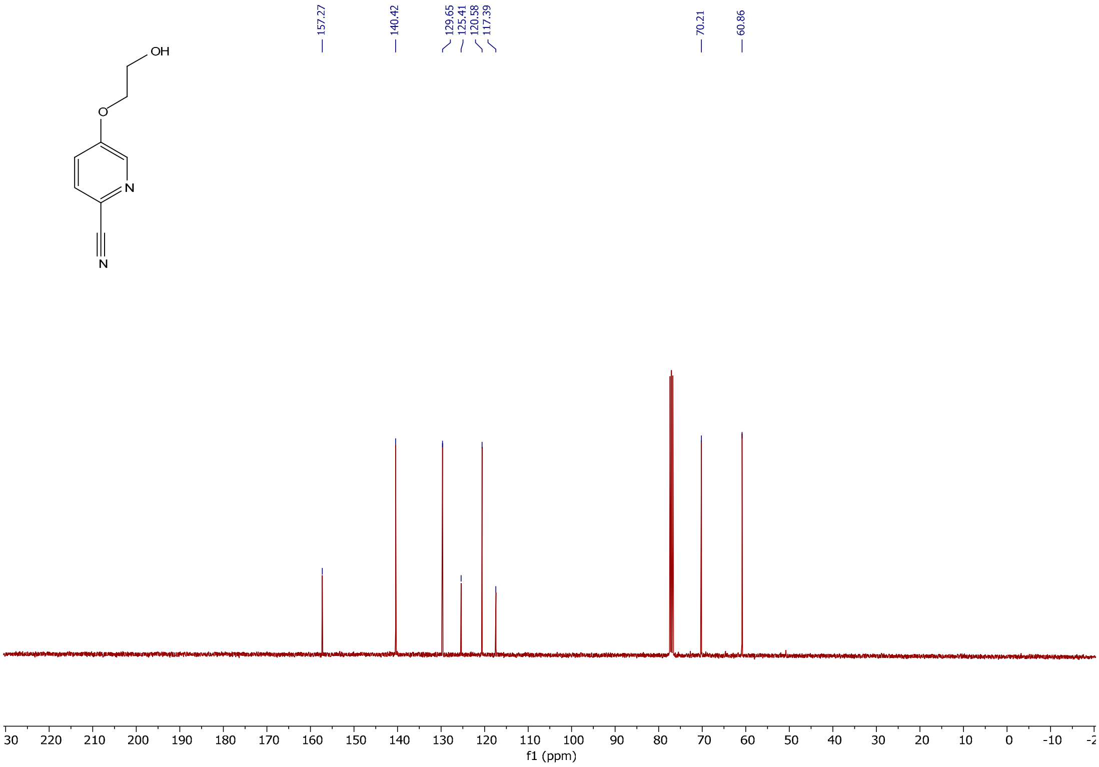

3-(5-(2-Fluoroethoxy)pyridin-2-yl)-6-(pyridin-2-yl)-1,2,4,5-tetrazine ***(41)***

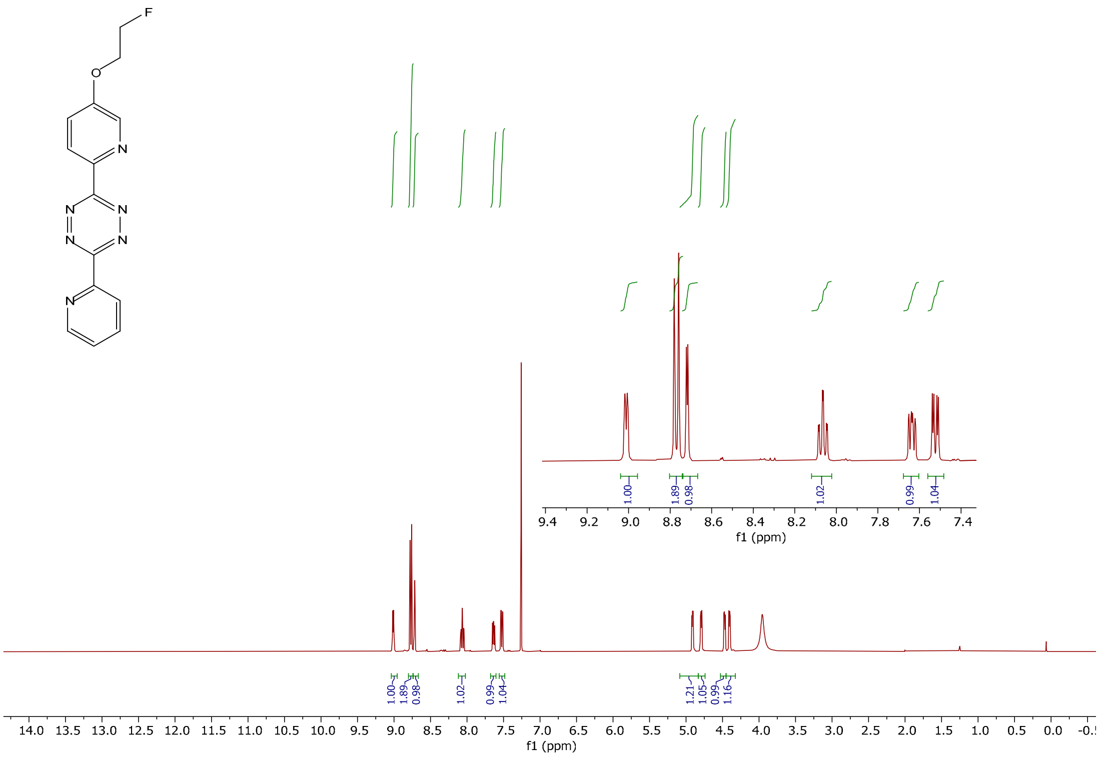

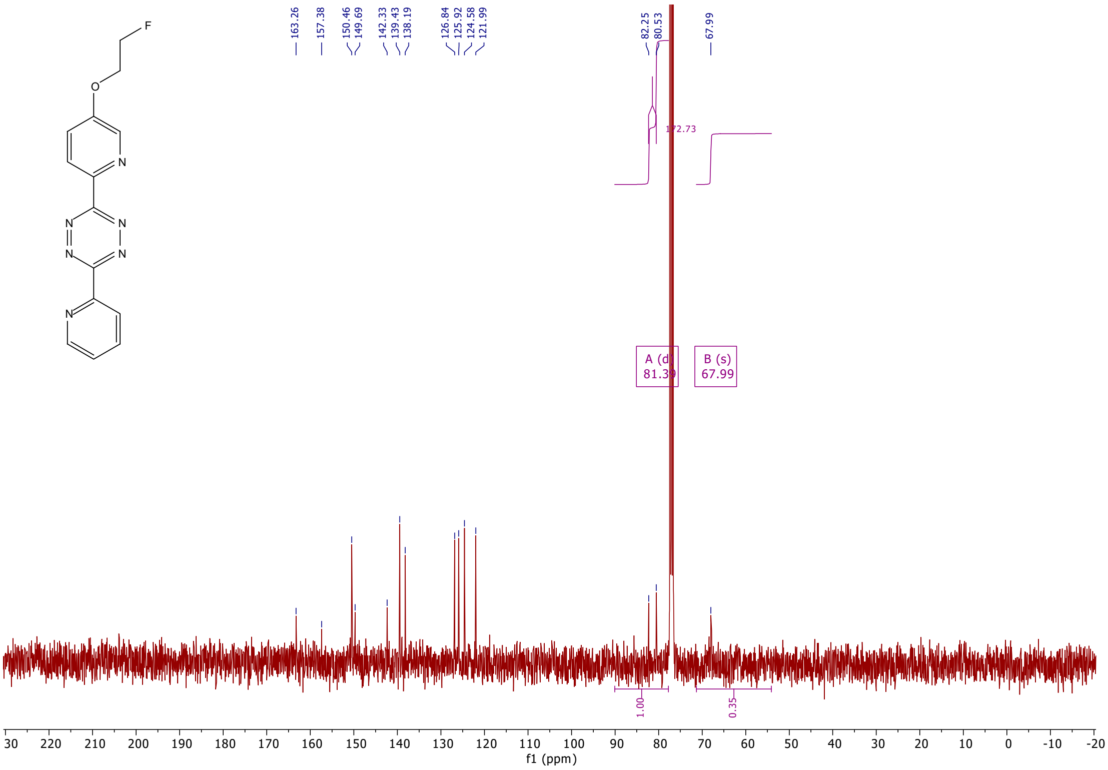

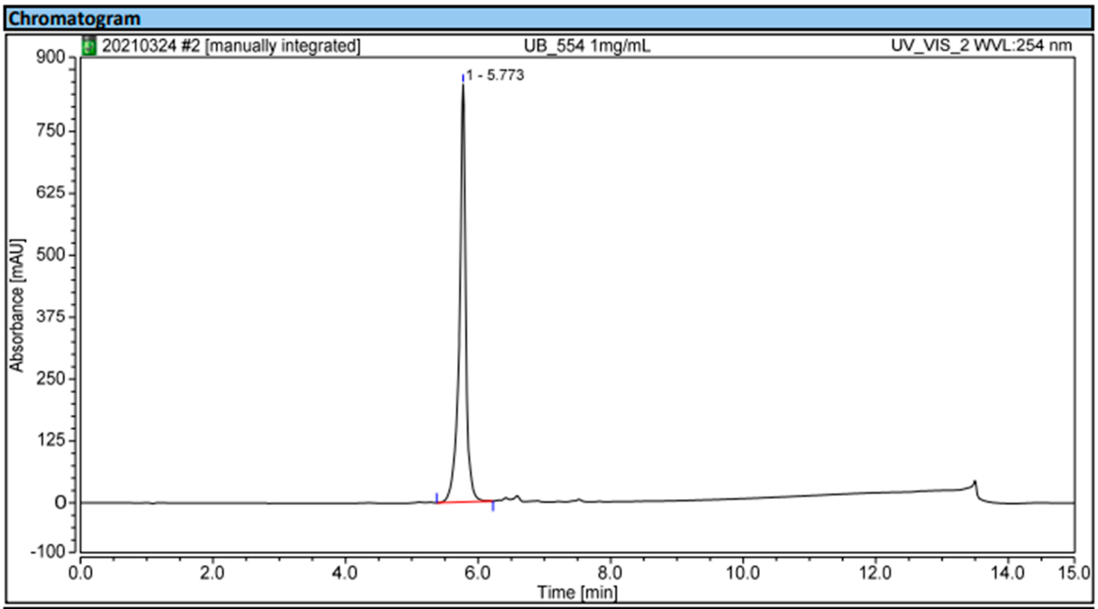

Integration Results

No.	Peak Name	Retention Time min	Area mAU*min	Height mAU	Relative Area %	Relative Height %
1		5.773	94.418	843.489	100.00	100.00
**Total:**			**94.418**	**843.489**	**100.00**	**100.00**

*2-((6-(6-(Pyridin-2-yl)-1,2,4,5-tetrazin-3-yl)pyridin-3-yl)oxy)ethan-1-ol (**42**)*

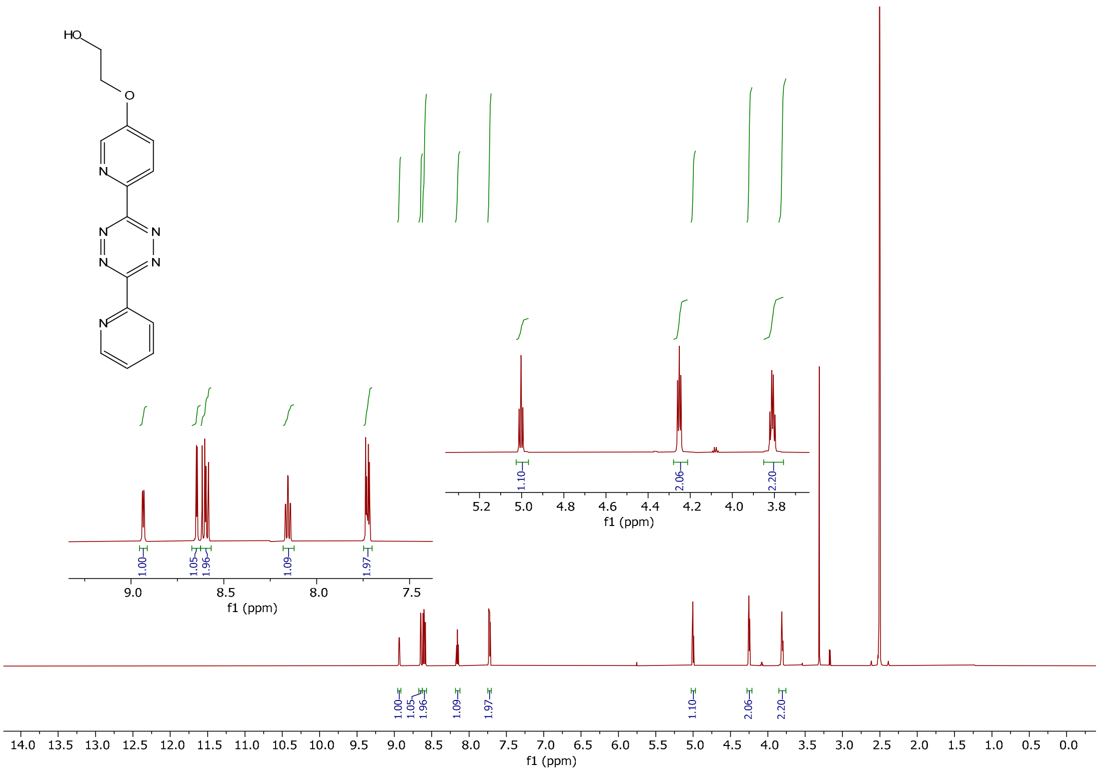

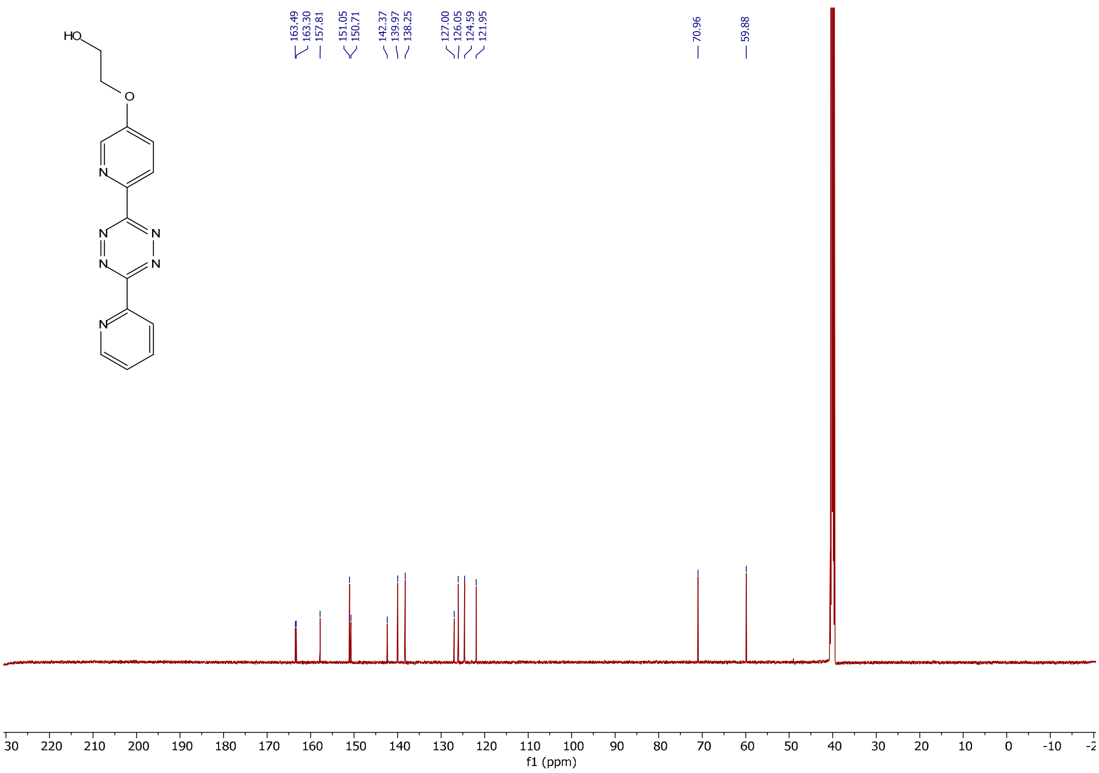

*Di-tert-butyl 2,2'-(((6-cyanopyridin-3-yl)methyl)azanediyl)diacetate (**43**)*

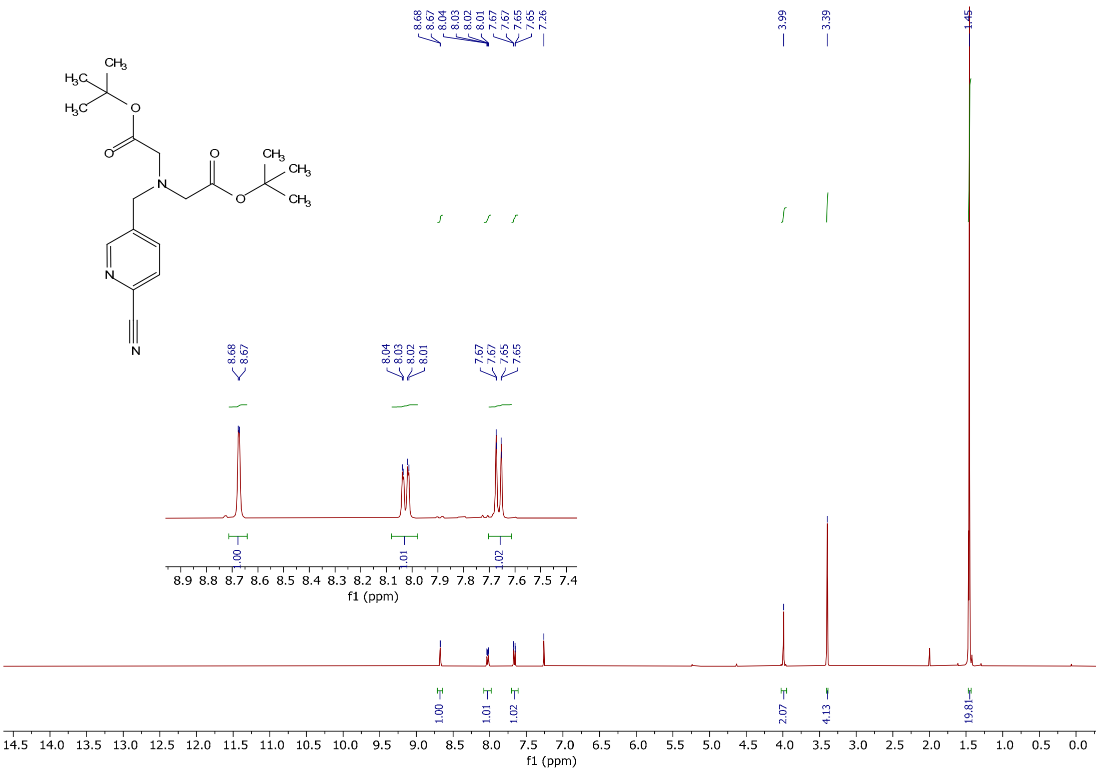
8.68
8.67
8.04
8.03
8.02
8.01
7.67
7.67
7.65
7.65
7.26
3.99
3.39
1.45
1.00
1.01
1.02
2.07
4.13
19.81
f1 (ppm)

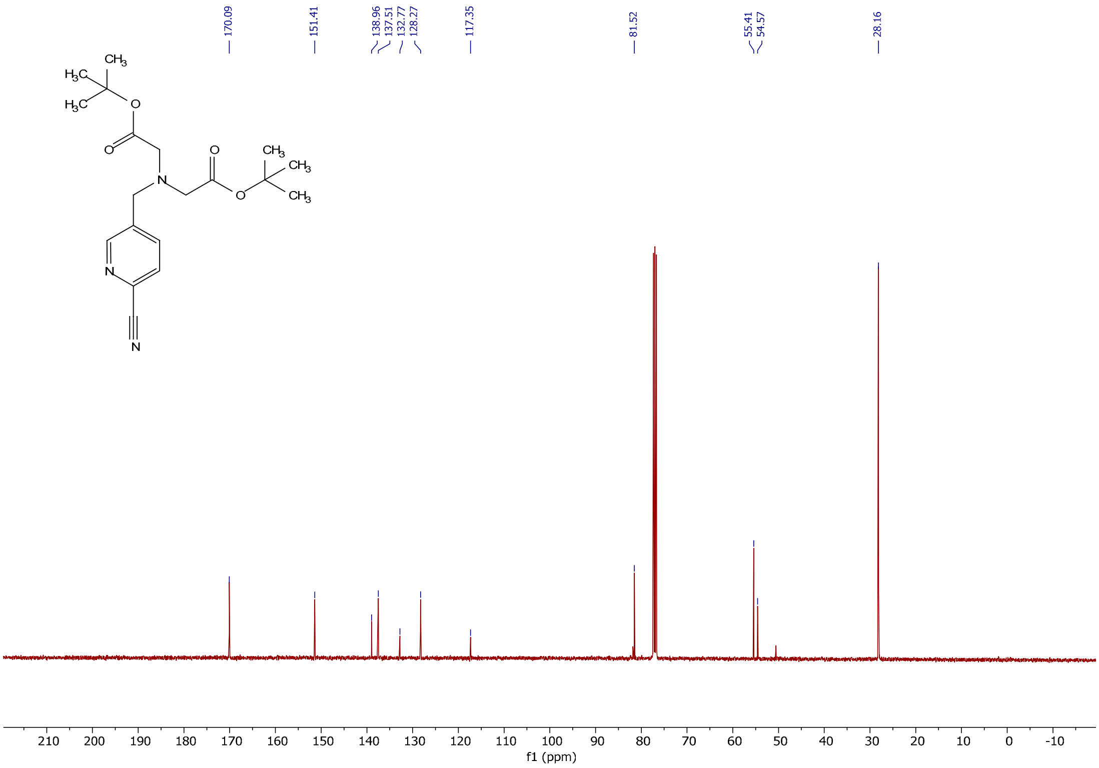
170.09
151.41
138.96
137.51
132.77
128.27
117.35
81.52
55.41
54.57
28.16
f1 (ppm)

*Di-tert-butyl 2,2'-(((6-(6-(5-(2-fluoroethoxy)pyridin-2-yl)-1,2,4,5-tetrazin-3-yl)pyridin-3-yl)methyl)- azanediyl)diacetate (**44**)*

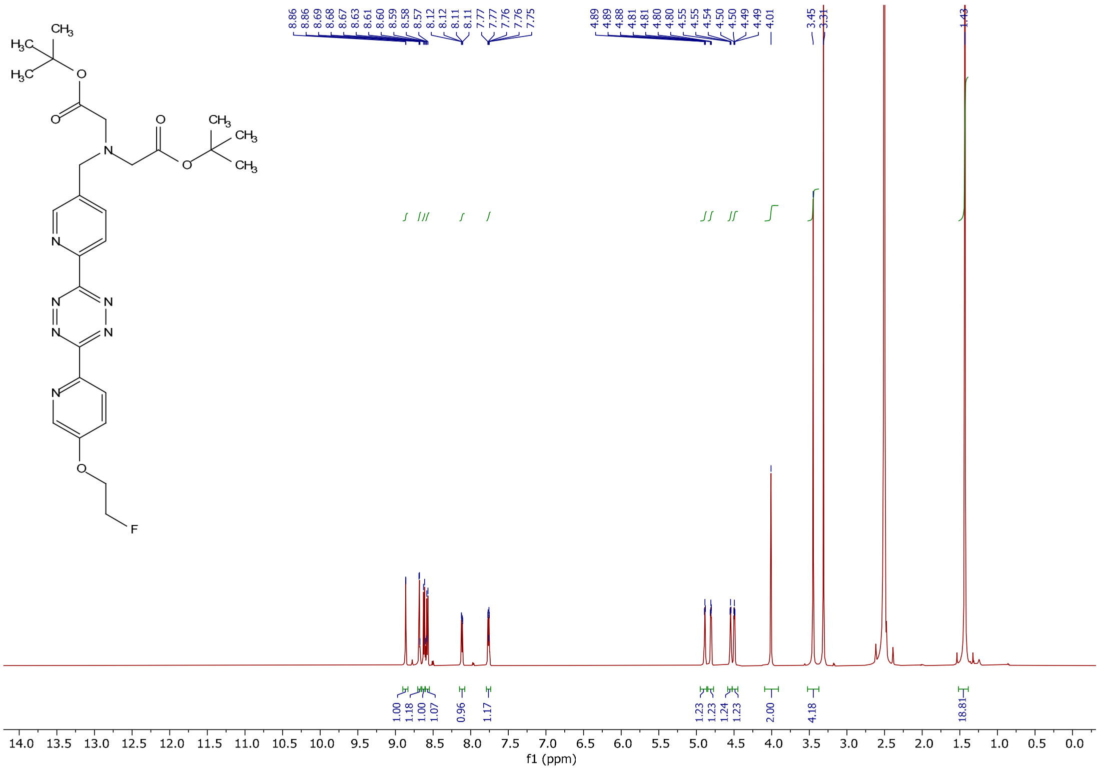

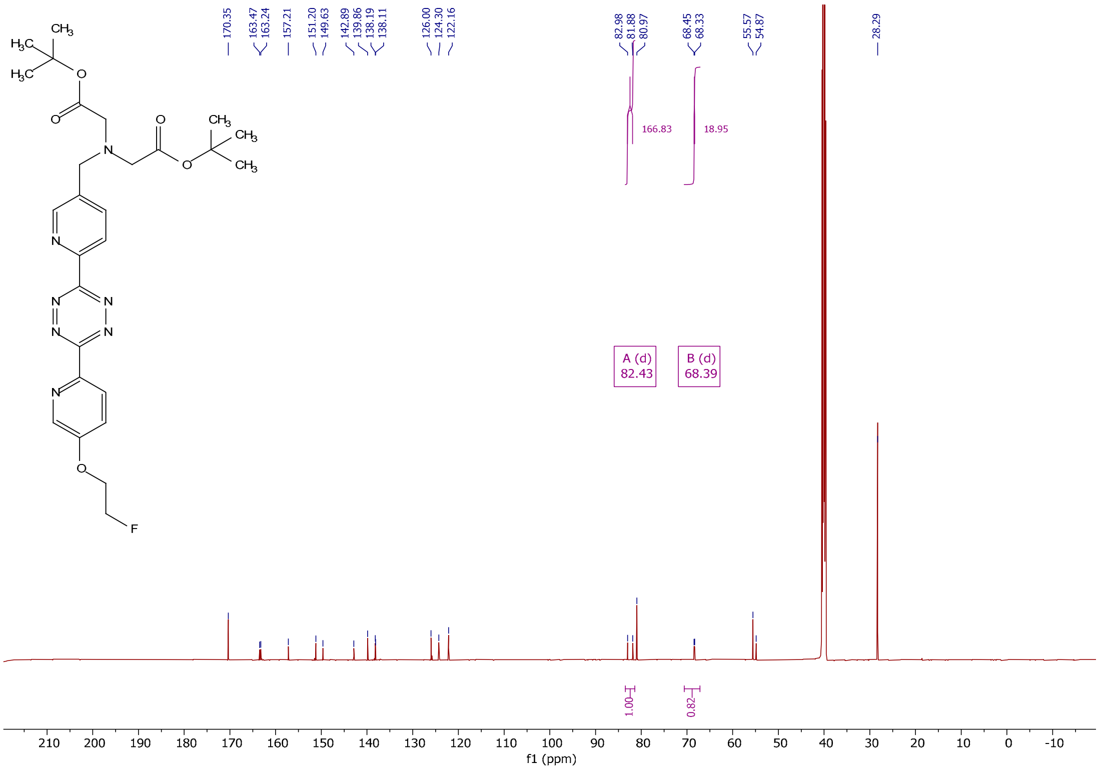

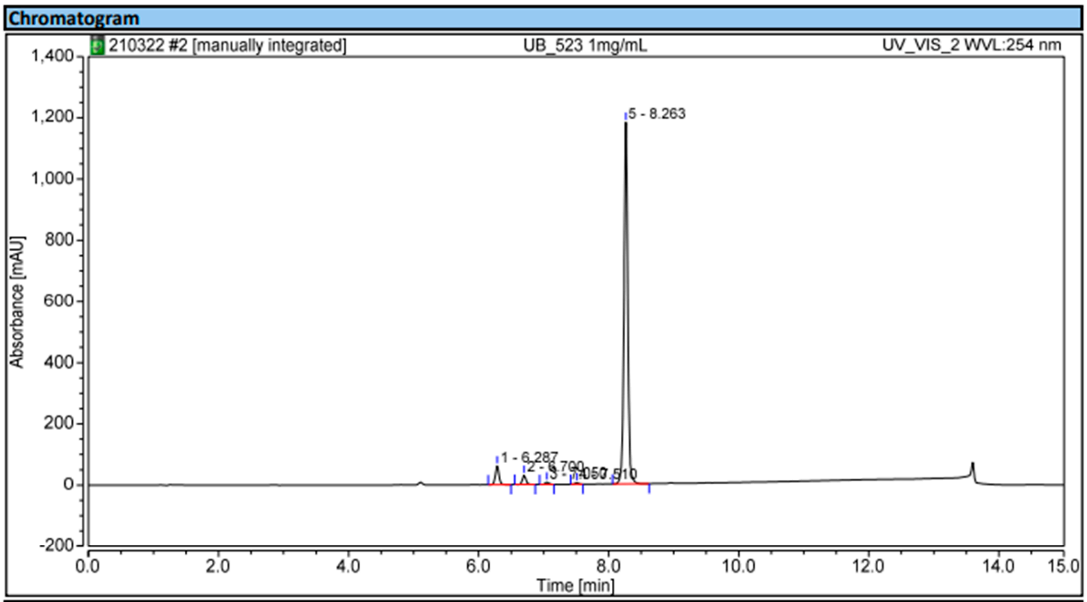

Integration Results

No.	Peak Name	Retention Time min	Area mAU*min	Height mAU	Relative Area %	Relative Height %
1		6.287	3.887	61.435	4.48	4.78
2		6.700	1.780	29.761	2.05	2.32
3		7.050	0.470	7.431	0.54	0.58
4		7.510	0.298	4.813	0.34	0.37
5		8.263	80.362	1181.589	92.59	91.95
**Total:**			**86.796**	**1285.028**	**100.00**	**100.00**

*2,2'-(((6-(6-(5-(2-fluoroethoxy)pyridin-2-yl)-1,2,4,5-tetrazin-3-yl)pyridin-3-yl)methyl) azanediyl)diacetic acid (**45**)*

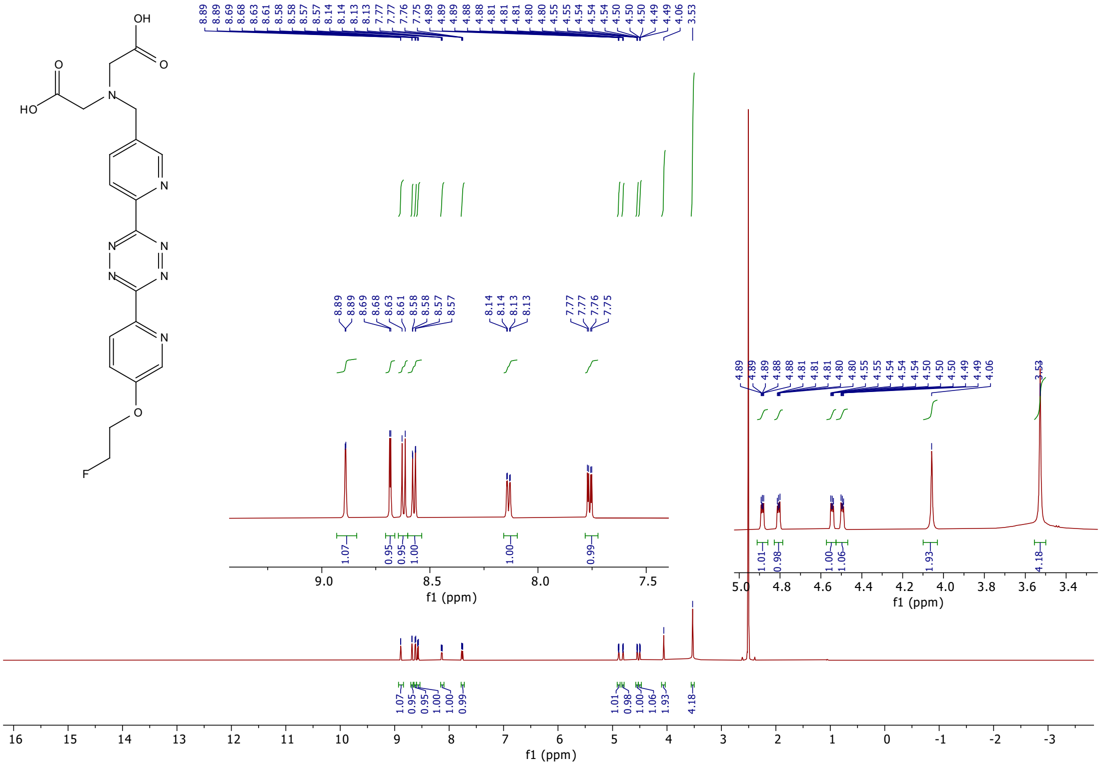

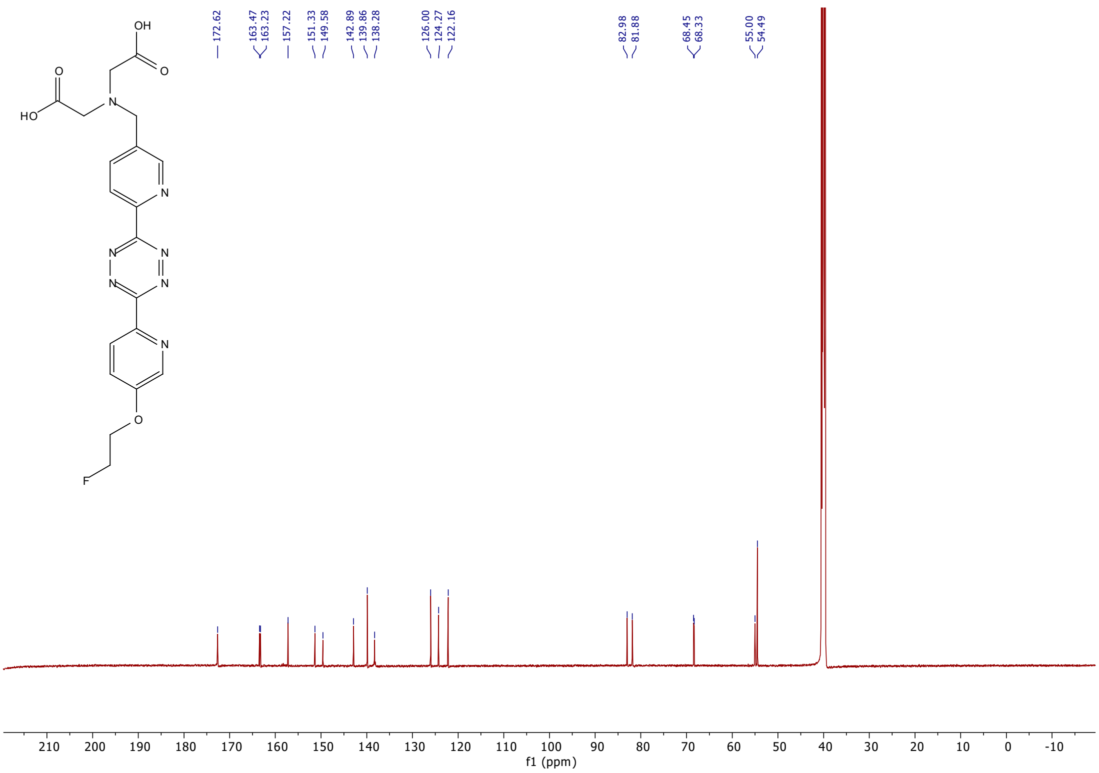

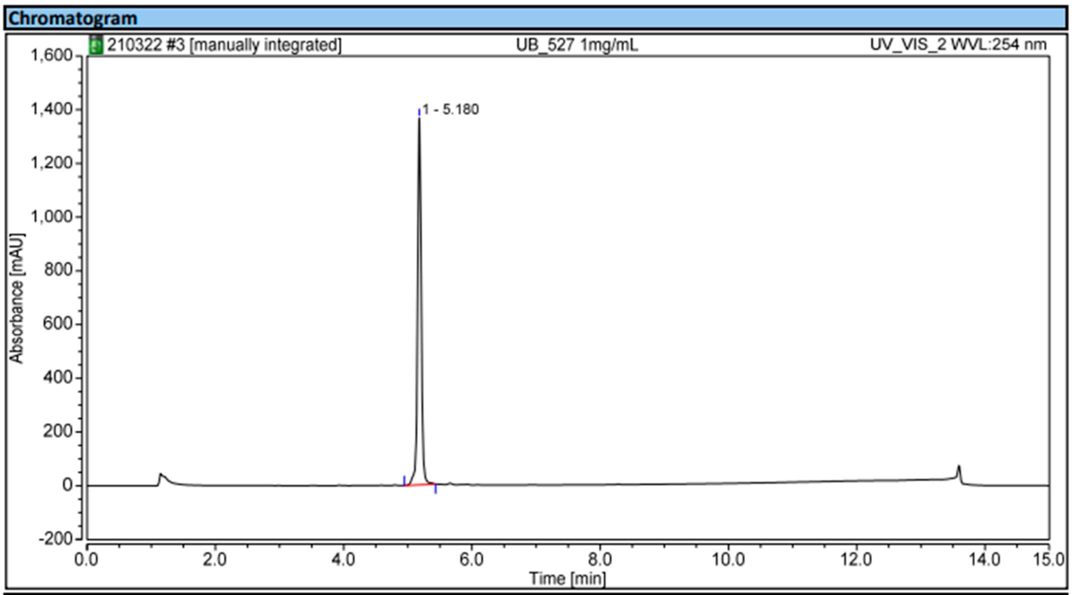

Integration Results

No.	Peak Name	Retention Time min	Area mAU*min	Height mAU	Relative Area %	Relative Height %
1		5.180	89.203	1364.904	100.00	100.00
**Total:**			**89.203**	**1364.904**	**100.00**	**100.00**

*Di-tert-butyl 2,2'-(((6-(6-(5-(2-(((4-nitrophenyl)sulfonyl)oxy)ethoxy)pyridin-2-yl)-1,2,4,5-tetrazin-3-yl)pyridin-3-yl)methyl)azanediyl)diacetate (**45a**)*

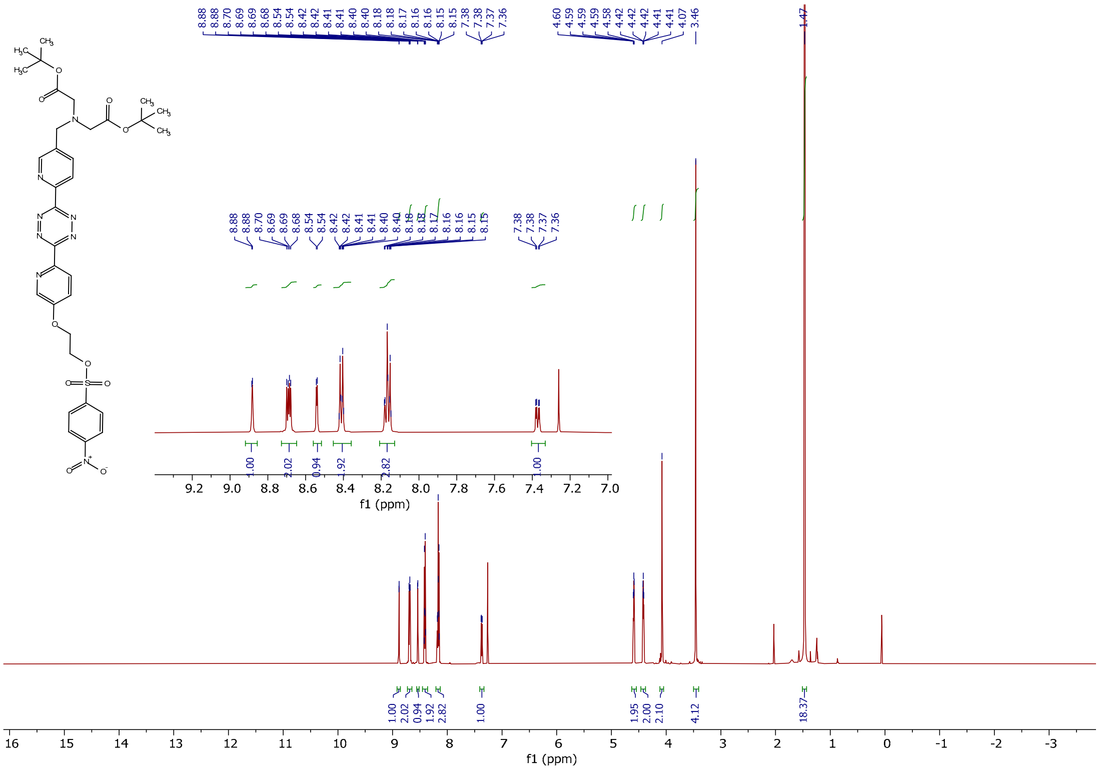

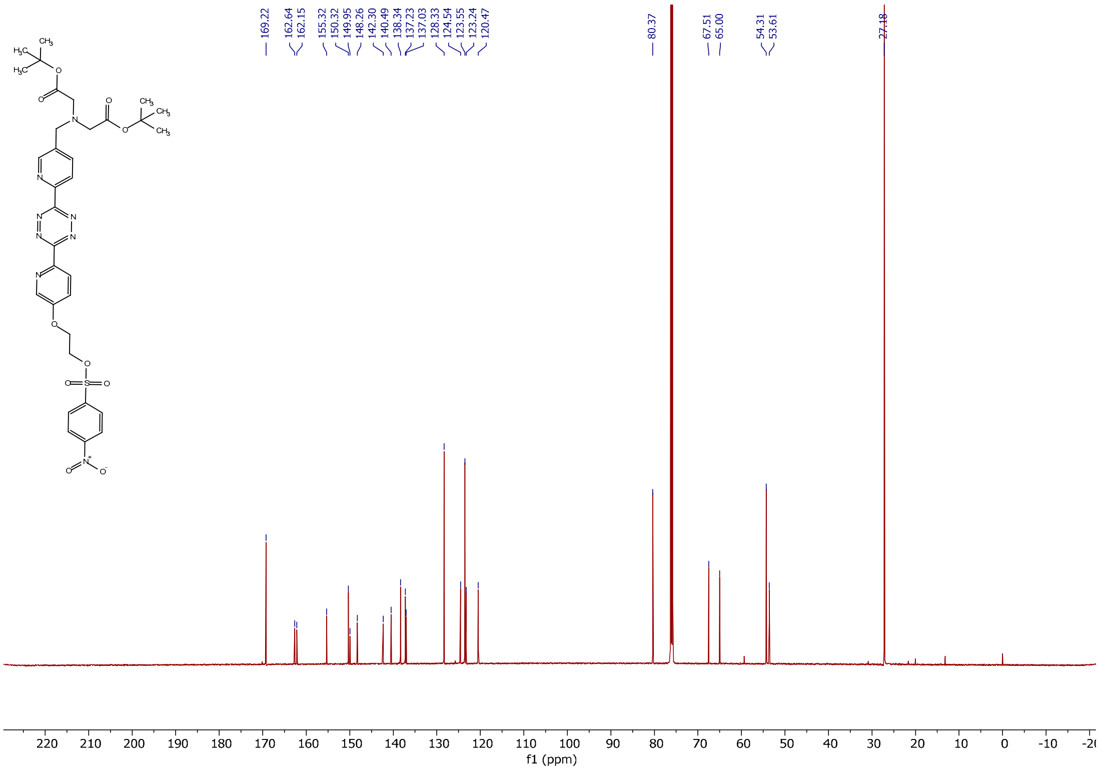

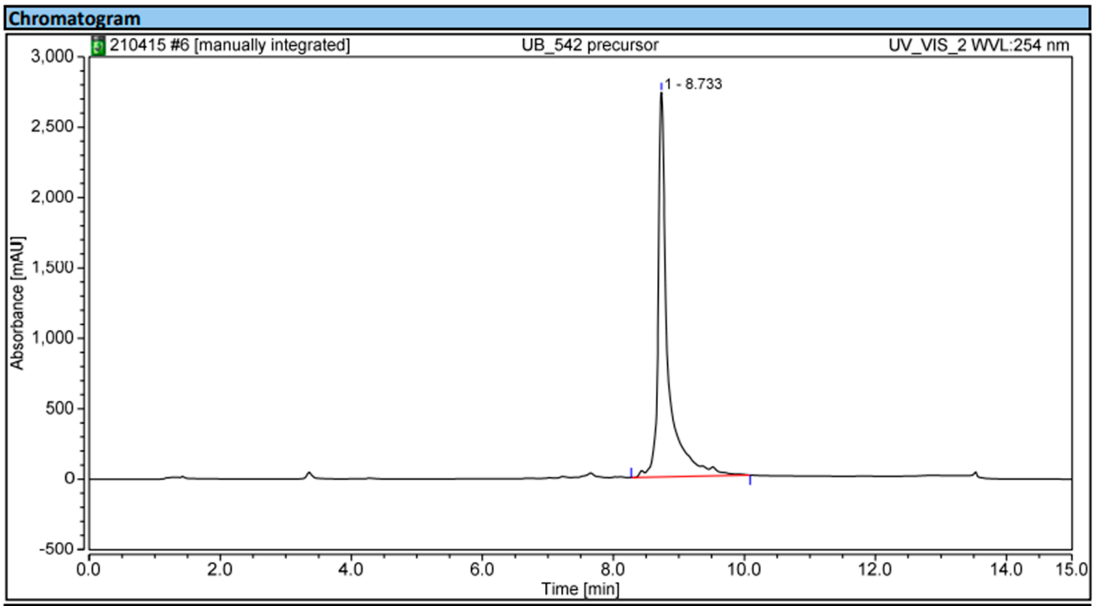

Integration Results

No.	Peak Name	Retention Time min	Area mAU*min	Height mAU	Relative Area %	Relative Height %
1		8.733	468.202	2731.135	100.00	100.00
**Total:**			**468.202**	**2731.135**	**100.00**	**100.00**

*Di-tert-butyl 2,2'-(((6-(6-(5-(2-hydroxyethoxy)pyridin-2-yl)-1,2,4,5-tetrazin-3-yl)pyridin-3-yl)methyl)azanediyl)diacetate (**46**)*

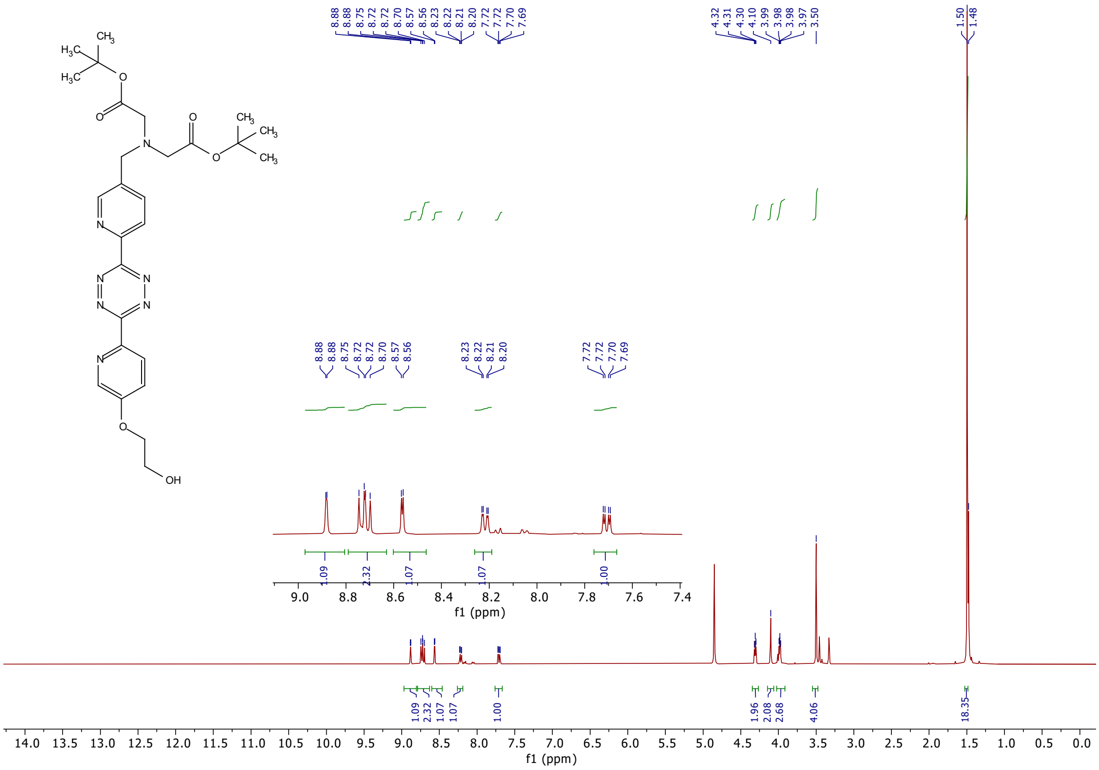

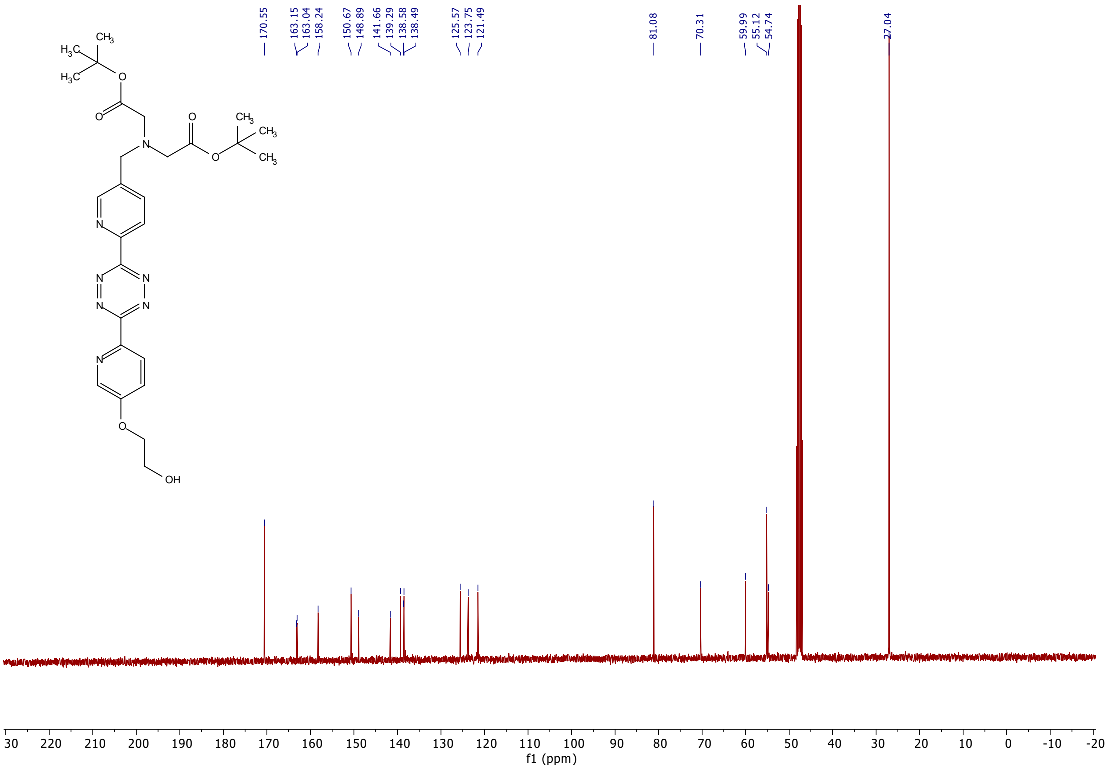

Labeling of compound [^{18}F]**1**

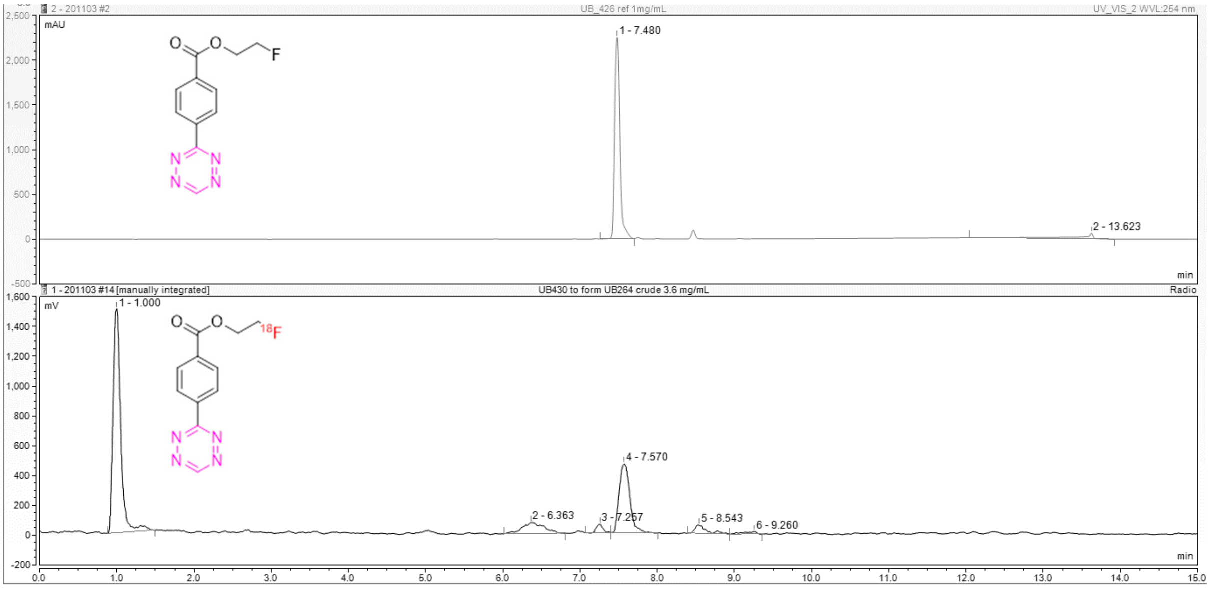

Labeling of compound [^{18}F]**8**

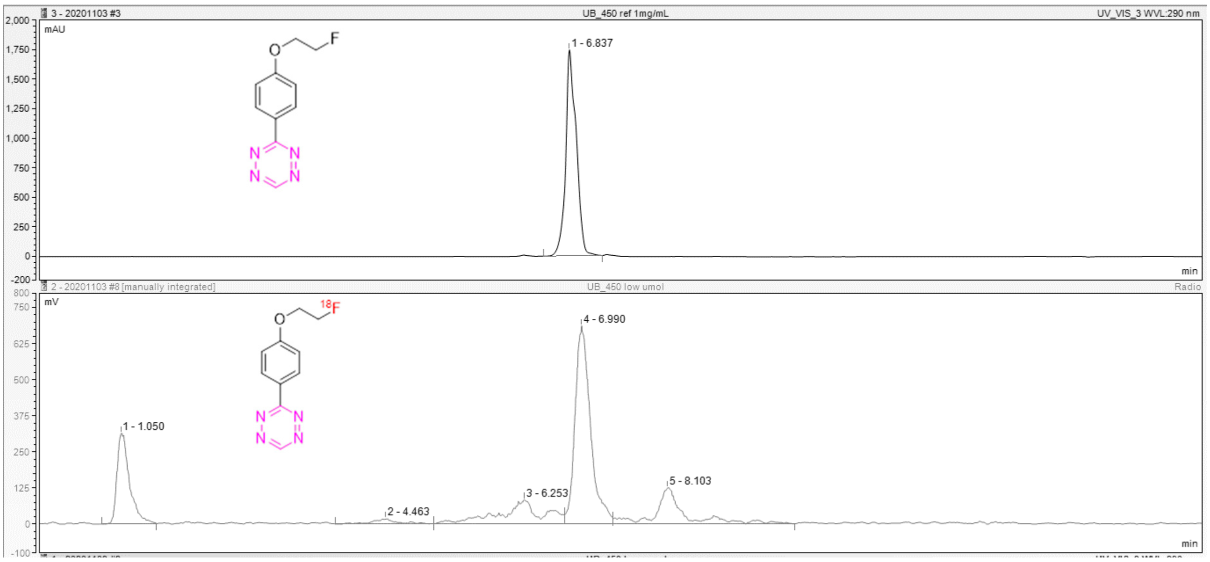

Labeling of compound [^{18}F]**33**

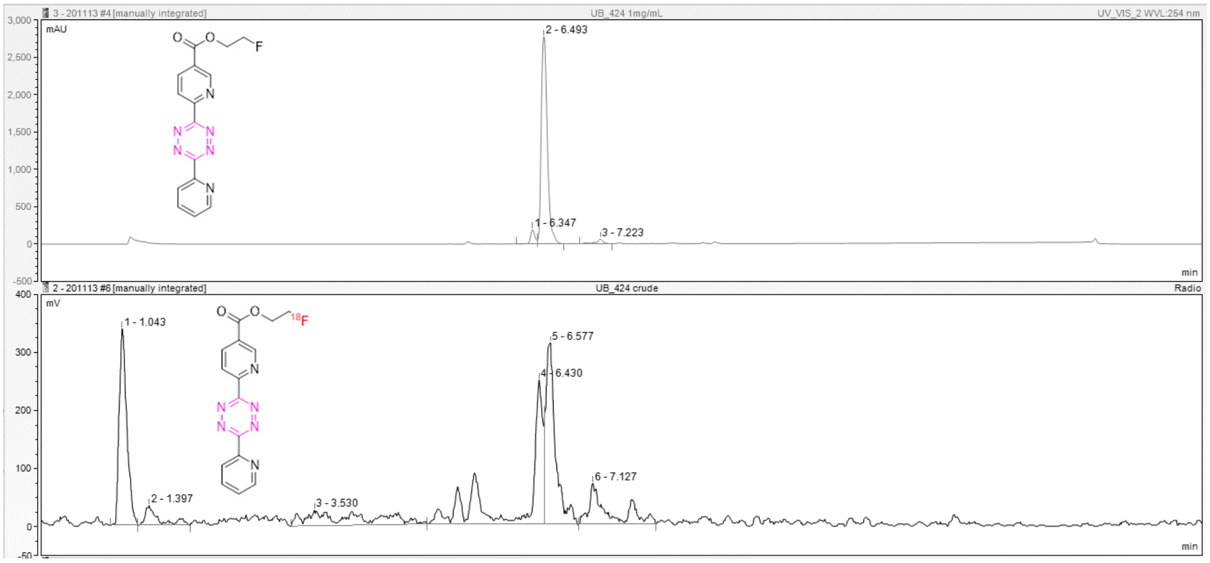

Labeling of compound $[^{18}F]$**38**

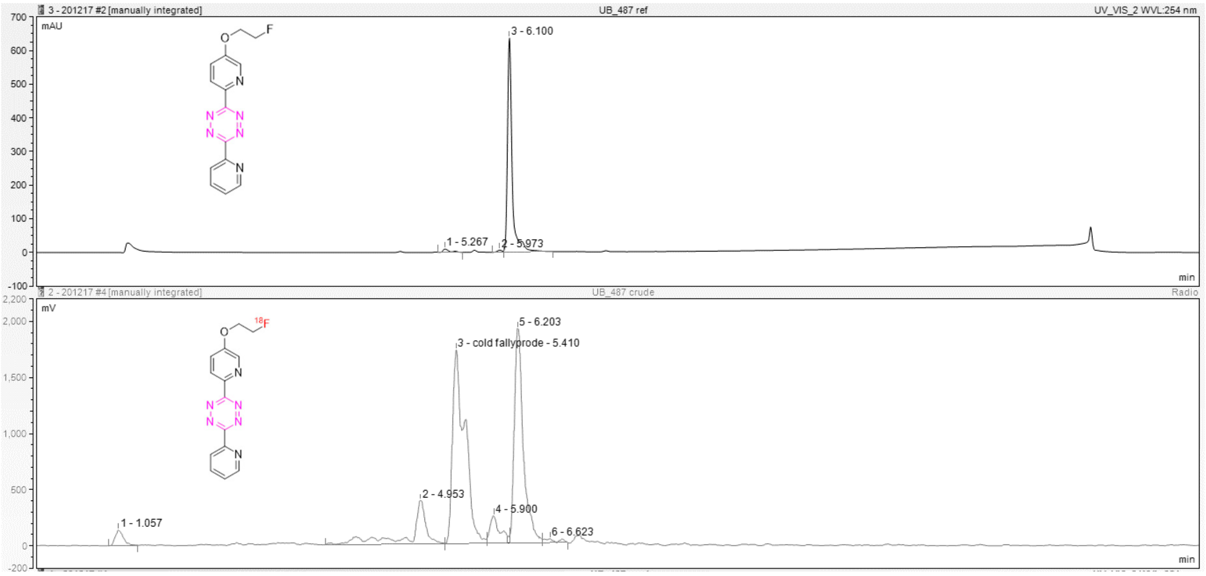

Labeling of compound $[^{18}F]$**41**

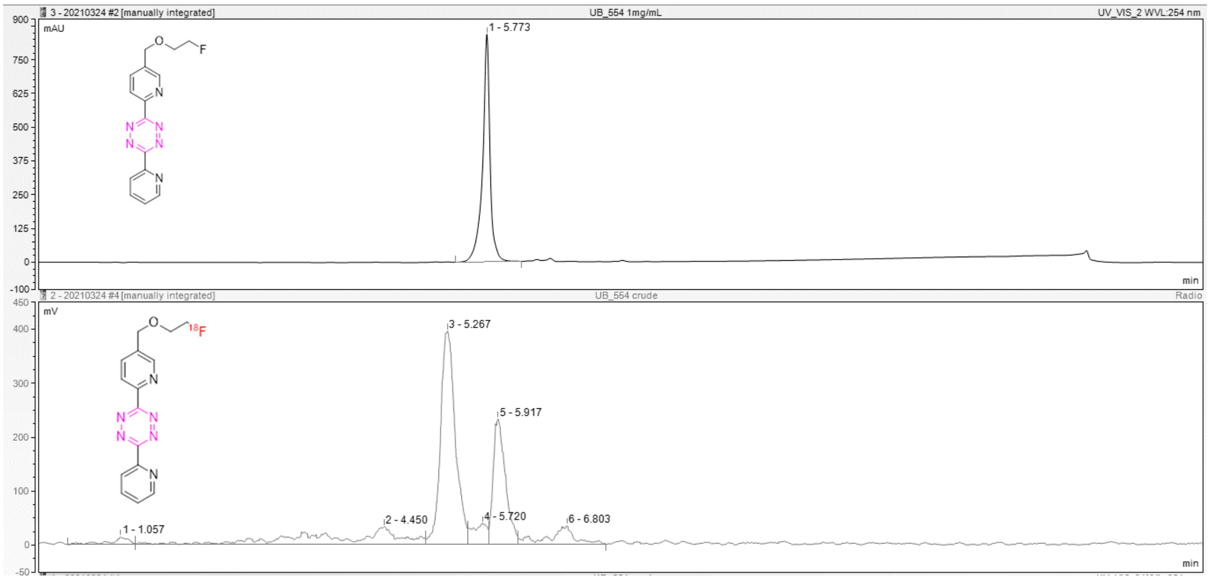

Labeling of compound [^{18}F]**44**

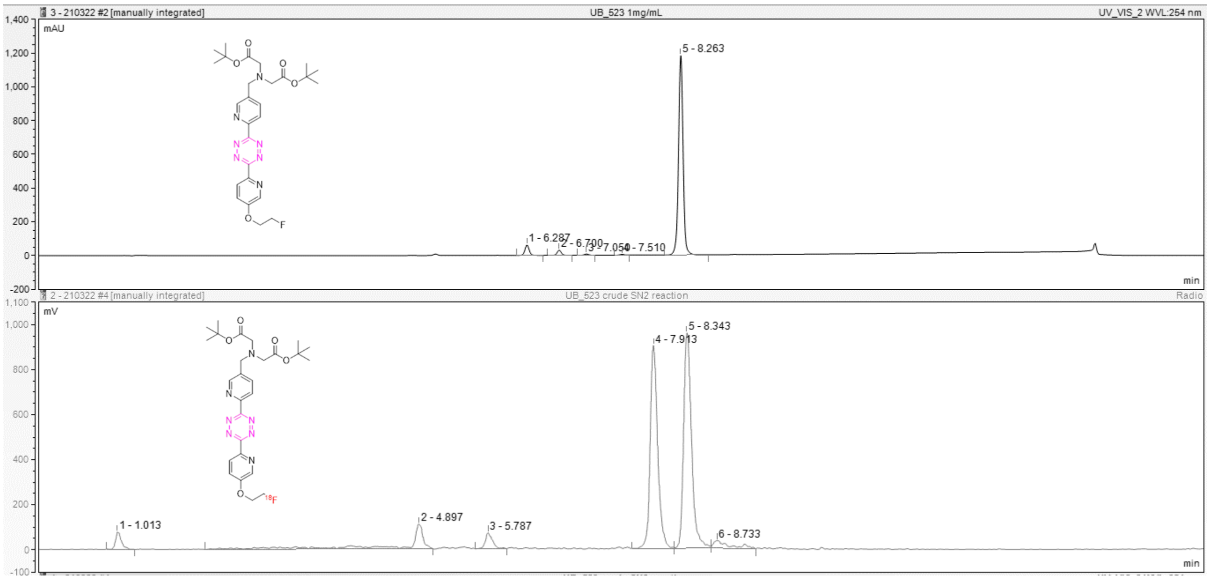

Deprotection of compound [^{18}F]**44**

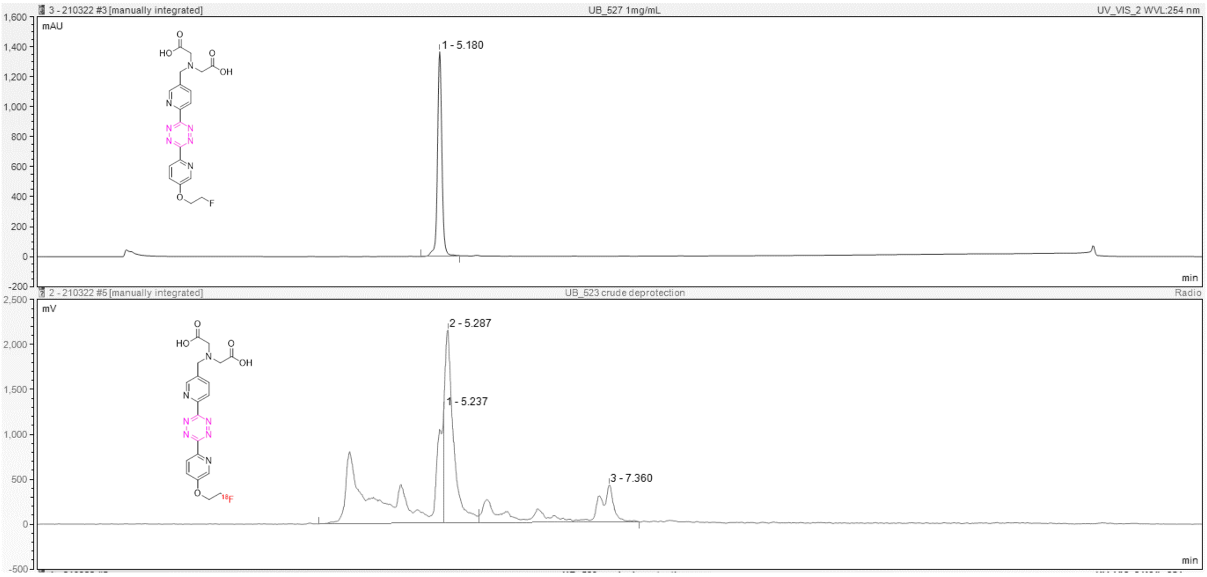

Supplement: Supplementary file 1 [file pharmaceuticals-15-00245-s001.zip › pharmaceuticals-1560505-supplementary.pdf]
